# Post-Translational Modifications of Proteins of Malaria Parasites during the Life Cycle

**DOI:** 10.3390/ijms25116145

**Published:** 2024-06-02

**Authors:** Evelin Schwarzer, Oleksii Skorokhod

**Affiliations:** 1 Department of Oncology, University of Turin, Via Santena 5 bis, 10126 Turin, Italy; evelin.schwarzer@unito.it; 2 Department of Life Sciences and Systems Biology, University of Turin, Via Accademia Albertina, 13, 10123 Turin, Italy

**Keywords:** *Plasmodium*, mosquito *Anopheles*, phosphorylation, acetylation, methylation, lipidation, lipoxidation, glycosylation, ubiquitination, glutathionylation

## Abstract

Post-translational modifications (PTMs) are essential for regulating protein functions, influencing various fundamental processes in eukaryotes. These include, but are not limited to, cell signaling, protein trafficking, the epigenetic control of gene expression, and control of the cell cycle, as well as cell proliferation, differentiation, and interactions between cells. In this review, we discuss protein PTMs that play a key role in the malaria parasite biology and its pathogenesis. Phosphorylation, acetylation, methylation, lipidation and lipoxidation, glycosylation, ubiquitination and sumoylation, nitrosylation and glutathionylation, all of which occur in malarial parasites, are reviewed. We provide information regarding the biological significance of these modifications along all phases of the complex life cycle of *Plasmodium* spp. Importantly, not only the parasite, but also the host and vector protein PTMs are often crucial for parasite growth and development. In addition to metabolic regulations, protein PTMs can result in epitopes that are able to elicit both innate and adaptive immune responses of the host or vector. We discuss some existing and prospective results from antimalarial drug discovery trials that target various PTM-related processes in the parasite or host.

## 1. Introduction

Malaria is still a very important, potentially life-threatening, infectious disease. Rather than being eliminated, slight case number growth has been registered recently [1,2]. The study of parasite metabolism, along with investigations into parasite–host and parasite–vector interactions, could propose new therapeutic strategies contributing to the eradication of malaria.

Malaria is caused by the parasite of *Plasmodium* genus, which has more than 150 described species that infect various species of vertebrates [3]. There are five parasite species that cause malaria in humans: *P. falciparum* (P.f.), *P. vivax* (P.v.), *P. ovale*, *P. malariae*, and *P. knowlesi*. Two of these species—P.f. and P.v.—are the most dangerous [4]. Rodent malaria parasite *Plasmodium berghei* (P.b.) is most studied in animal malaria models [5].

The *Plasmodium* life cycle involves a complex alternation between humans (as an example of a *Plasmodium* host) and mosquitoes. It begins when an infected female *Anopheles* mosquito bites a human, injecting sporozoites into the bloodstream. After a brief “skin malaria” period, sporozoites migrate to the liver, where they infect hepatocytes and undergo hepatic schizogony, producing merozoites. The period of schizonts maturation is different for *Plasmodium* species, for example, the dormant stage (hypnozoites) of P.v. and *P. ovale* could last for years. After liver schizogony merozoites are released into the bloodstream, they invade erythrocytes and undergo erythrocytic schizogony, leading to the release of numerous merozoites, which infect new host erythrocytes. After erythrocyte invasion, some sexually committed merozoites differentiate into male and female gametocytes, marking the start of the sexual reproduction phase. During a blood meal, the mosquito ingests circulating gametocytes. Inside the mosquito’s midgut, male and female gametocytes form the zygotes. The zygotes develop into ookinetes, which traverse the mosquito gut wall. Subsequently, ookinetes are transformed into oocysts, initiating sporogony and producing thousands of sporozoites. The sporozoites migrate to the mosquito’s salivary glands, ready to infect another human during the mosquito blood meal, completing the cycle [4].

Metabolic processes are very active and highly adaptable in *Plasmodium* spp. [6]. Post-translational modifications (PTMs) of proteins are believed to enhance the functional diversity of the proteins by the covalent attachment of chemical groups or other proteins, the cleavage of regulatory or other subunits through proteolysis, or the degradation of the proteins. Protein PTMs comprise mostly phosphorylation, glycosylation, ubiquitination, nitrosylation, methylation, acetylation, lipidation, and proteolysis. The impact may concern every facet of both normal cell biology and pathogenesis. Thus, the identification and comprehension of PTMs play a crucial role in advancing our understanding of cell biology and the invention of treatments and preventive measures for various diseases.

## 2. Protein PTMs in *Plasmodium* Parasite Growth and Development

**Figure 1:**
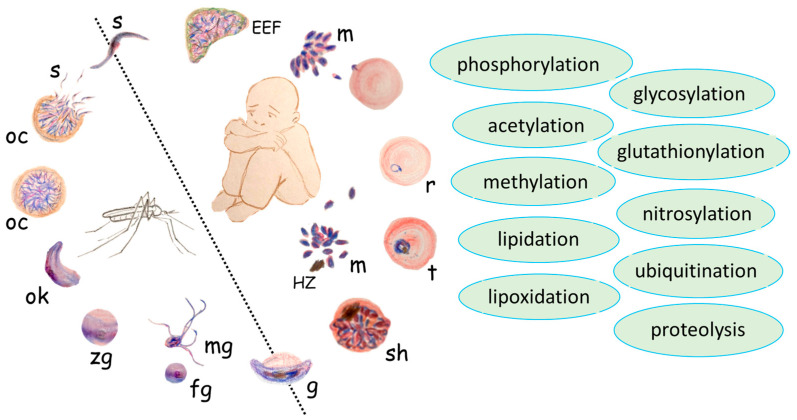
The life stages of the malaria parasite *Plasmodium* within both the host and the vector, along with protein post-translational modifications (PTMs) that accompany the parasite life cycle. The parasite stages include sporozoites (s), liver exoerythrocytic forms (EEFs), merozoites (m) (after egress from hepatocytes or erythrocytes, with malarial pigment hemozoin (HZ) released from infected erythrocytes), ring forms (r), trophozoites (t), schizonts (sh), gametocytes (g), male (micro-) or female (macro-) gametes (mg and fg, respectively), zygotes (zg), ookinetes (ok), and oocysts (oc). The life stages in the vector (mosquito) are separated by a dotted line from those in the host (human). Protein PTMs are present or implicated in all parasite stages.

A high quantity of metabolic processes drive parasite development. The PTMs of proteins, as an essential part of metabolism, play a pivotal role in parasite growth and development [7,8,9]. A fairly comprehensive review regarding PTM in *Plasmodium* species was published in 2009: known at the time as phosphorylation/dephosphorylation, acetylation, methylation, lipidation, ubiquitination, and protein cleavage/processing were considered [7]. Other important reviews regarding protein PTM in malaria parasites were published in 2015 and 2021, focusing on the functional significance of protein PTMs [8,9]. In this present review, we analyze recent data and, notably, include some “minor” PTMs that were previously overlooked, such as certain forms of alkylation and lipoxidation (e.g., 4-hydroxynonenal conjugation). We summarize the published evidence regarding the presence and, if known, the role of all post-translational modifications (PTMs) in *Plasmodium* parasites throughout their life cycles, starting from sporozoites in the host (Figure 1).

### 2.1. Sporozoites in the Host

Sporozoites are less studied *Plasmodium* forms regarding PTMs. Existing studies analyzed sporozoites from their initial oocyst differentiation, replication, egress, and migration through the hemolymph to the mosquito salivary glands. Consequent transmission to the host is crucial for malaria spread. Sporozoites are transmitted to the host by the mosquito during the blood meal and move from the skin to the liver for further hepatocyte invasion.

As in other organisms, the majority of transcription and translation initiation in *Plasmodium* is regulated by phosphorylation/dephosphorylation, a process that is well understood but still requires further study [10,11,12,13]. Kinases are generally distributed across various processes in *Plasmodium*, including cell-cycle regulation, cell proliferation and differentiation, sexual differentiation, parasite egress and invasion, and host–parasite interaction [7,14]. Kinases are poorly reported in sporozoites. Kinases essential for *Plasmodium* sexual stages (e.g., MAP-2, CDPK4, NEK-4 for male gametocytes, ookinete, or oocyst) appear to be important for sporozoite maturation in mosquitoes [15]. However, their significance was not entirely clear regarding the invasion of the mosquito’s salivary glands and sporozoite infectivity [15]. The phosphorylation of myosin A, instead, was shown to regulate the gliding motility in the salivary gland of P.b. and to be essential for *Plasmodium* transmission [16]. The P.f. transcriptome shows that the transcription of the calcium-dependent protein kinase-6 (PfCDPK-6) is significantly upregulated in the sporozoite stage [17] and is critical for the invasive sporozoite phenotype, which exploits host heparan sulfate proteoglycans (HSPGs) [18].

Generally, the steady-state kinetics of the *Plasmodium* histone-lysine N-methyltransferase PfSET7 enzyme are similar to those of previously characterized histone methyltransferase enzymes from other organisms. However, PfSET7 shows a preference for specific protein substrates, H3K4 and H3K9, and to a lesser extent H3K36, particularly towards nucleosomes with pre-existing histone H3 lysine 14 acetylation. The functional significance of H3K4, H3K9, and H3K36 methylation by PfSET7 is only partially understood. Interestingly, PfSET7 localizes to distinct cytoplasmic foci adjacent to the nucleus in erythrocyte-stage and liver-stage parasites, and throughout the cytoplasm in salivary gland sporozoites [19], which may be indicative for different activities of the enzyme at different sporozoite life stages.

Due to its importance in vaccine development, the circumsporozoite protein (CSP), one of the major surface proteins of sporozoites, and its cell distribution, shedding, and any PTM are of high interest for malariologists [20]. P.v. salivary gland sporozoites mass spectrometry-based proteomics individuated approximately 2000 proteins and their PTMs. Significant phosphorylation was found in glideosome proteins, along with transcription and translation regulators [21]. The glycosylation of CSP and thrombospondin-related adhesive protein (TRAP), initially identified in P.f. salivary gland sporozoites [22], were similarly observed in P.v. sporozoites [21]. Fucosylation (the attachment of a fucose residue to glycoproteins), and particularly O-fucosylation of CSP and TRAP by the *Plasmodium* O-fucosyltransferase (POFUT2), was shown. This is supposed to be a part of the protein folding quality control mechanism. The defects observed after genetic disruption of P.f. POFUT2 are attributable to destabilization and the incorrect trafficking of these surface proteins. Hence, the protein fucosylation was assumed to be important for *P. falciparum* transmission [23]. The functional consequences of thrombospondin type 1 repeat (TSR) domain modification by O-fucosylation vary substantially between species as concluded from the comparison of transmission efficiencies between human and rodent malaria parasites [24].

The proteolytic cleavage of the CSP was shown as functional PTM in rodent malaria models with P.b. The cleavage is necessary to convert CSP into its active form, with the exposure of specific regions of the protein that are involved in the interaction with host cell receptors. These interactions are crucial for the attachment of the sporozoites to host cells and the subsequent invasion process [18]. Studies in P.b. have shown that the eukaryotic initiation factor-2alpha (eIF2alpha) kinase (IK2 or eIK2) plays a major role in maintaining translational shutdown of CSP in “young” sporozoites. When sporozoites are injected into the mammalian host, the eIF2alpha phosphatase UIS2 removes the phosphate from eIF2alpha-P. This action abolishes the repression of translation, allowing for CSP expression, processing, and, ultimately, the active invasion of hepatocytes by the sporozoites, as well as their transformation into liver stages [25,26,27]. Similar results were also obtained for P.f., where PfeIK1 kinase through eIF2alph phosphorylation regulated the stress response to amino-acid starvation [28].

Cysteine proteinase SERA-8, a member of the serine-repeat antigen proteinases (SERAs), was identified as an important cleavage/processing enzyme for CSP in sporozoites [17,29]. In the host, the invasion of hepatocytes by sporozoites is shown to be regulated by shedding the apical membrane antigen-1 (PfAMA-1) and TRAP through the action of a serine protease [30]. PfAMA-1 and its post-translational modifications (PTMs) were additionally studied in silico, since AMA-1 is considered a promising vaccine candidate. This is due to its expression on both sporozoites and merozoites, as well as its crucial role in the invasion of hepatocytes and erythrocytes, respectively. Putative glycosylation sites in AMA-1 were found at Thr517 and Ser498. O-GlcNAc-AMA-1 may serve as a conformationally more suitable antigen for eliciting a protective immune response against malaria compared to the non-modified AMA-1 [31].

Two cysteine proteases, the autophagy-regulating protease Atg4 [32] and the ovarian tumor unit (Otu), have been identified to delipidate Atg8 (lipid modifications are largely reviewed in Section 2.3), detaching it from parasite membranes. This process has been demonstrated to be crucial for the life cycle of P.b. parasites, playing a vital role in sporozoite maturation within mosquitoes, liver invasion in the mouse host, and maturation to hepatic merozoites [33].

Ubiquitination (ubiquitylation), the binding of ubiquitin or its analogs to proteins, is another important PTM in sporozoites. Ubiquitin is a highly conserved 76 amino acid protein encoded by three quite different types of genes UbL40, UbS27a, and pUb in *Plasmodium* spp. [34]. Their specific distribution and the abundance between the *Plasmodium* spp. and parasite stages are currently under study and discussion [34]. Nevertheless, ubiquitination and de-ubiquitination is active in all Plasmodium stages. High-throughput screening identified covalent fragment inhibitors for P.f. deubiquitinase ubiquitin C-terminal hydrolase L3 (PfUCHL3) with antimalarial activity against asexual P.f. blood stages and P.b. sporozoite stages [35], demonstrating the importance of ubiquitination for sporozoite development.

Palmitoylation, a reversible and dynamic lipid-based PTM in which a fatty acid, typically palmitic acid, is attached to a cysteine residue of a protein. This modification can affect the localization, stability, and function of the protein within the cell membrane (more lipid modifications are reviewed in Section 2.3). Palmitoylation was shown to be important in sporozoites. DHHC3 enzymes from the Asp-His-His-Cys (DHHC) family of palmitoyl acyltransferases (PATs) play a critical role in the development of mosquito and liver stages of parasites. P.b. ookinetes and sporozoites deficient in DHHC3 show compromised gliding motility and display a pronounced phenotype in vivo. Ookinetes exhibit significantly reduced infectivity to their mosquito host, while sporozoites fail to infect mice. The genetic reintroduction of DHHC3 into the knockout parasite restores virulence, underscoring the significance of palmitoyl transferases as promising targets for therapeutic interventions [36].

Modified sporozoite proteins, their PTMs, and the enzymes involved are summarized in Table 1.

### 2.2. Liver Forms (from Sporozoites to Exoerythrocytic Forms, EEFs)

Liver forms of *Plasmodium*, known as exoerythrocytic forms (EEFs), directly originate from sporozoites. The process of hepatocyte invasion by sporozoites is determined by both sporozoite and host conditions. After sporozoite invasion, EEFs develop, displaying unique characteristics in growth and interaction with the host, which are also mirrored in the protein PTMs.

Phosphorylation and consequent proteolysis have been shown to play a crucial role in hepatocyte invasion by sporozoites. The kinase activity is also highly pronounced during the active multiplication of EEFs. Confirming the importance of cyclic GMP (cGMP)-dependent protein kinase (PKG) in various stages of the *Plasmodium* life cycle, the liver stage PfPKG directly phosphorylated the parasite RPT1, one of the six AAA+ ATPases (subunits) present in the proteasome 19S regulatory cap. Both, the PKG activity and proteolysis were functionally implicated in the invasion of HepG2 cell line hepatocytes by P.b. sporozoites [45].

Another protein kinase, PfPK9, was shown to be important for liver stage *Plasmodium*, as the inhibition of PfPK9 resulted in impaired parasite development (see also the Section 3) [46].

The FIKK family protein kinase PbMLFK was shown to be expressed only during the mosquito and liver stages [47], while other FIKK were also expressed in the erythrocyte stage (see the Section 2.3). PbMLFK contains two functional C-terminal PEXEL motifs (Plasmodium Export Element, a specific sequence motif associated with proteins exported into the host cell). It has an important role during the mosquito stage and plays a crucial role in the parasite’s growth within the livers of infected mice. Notably, this kinase indirectly, but intensively, interacts with host liver cells, and transcriptome data from infected host cells indicate that the absence of the kinase leads to the altered expression of 288 host cell mRNAs [47].

Mouse liver receptor EphA2 was shown to be crucial for sporozoite invasion, and the phosphorylation of EphA2 was induced by the parasite, probably in a roundabout way [48].

Although TRAP has already been mentioned in the “Sporozoites” paragraph due to its physiological importance and its modifications related to sporozoite development in mosquitoes and liver invasion, it is worth noting that TRAP phosphorylation has been detected in P.f. parasites during their hosting in HepG2 hepatocytes. The binding of PfTRAP to SH3-domain containing Src-family kinases (Lyn, Lck, and CrkL) and its phosphorylation by the Src-kinases have been experimentally shown, suggesting a role for PfTRAP in cell signaling during both sporozoite invasion and homing inside liver cells [49]. Additionally, the P.b. subtilisin-like SUB1 protease (plausibly responsible for processing MSP1, MSP6, and MSP7) is important for the egress of malaria parasites from hepatocytes [50].

The enzyme Acetyl-CoA carboxylase (ACC) is expressed in the apicoplast during both the liver and blood stages, but is activated by biotin ligase through biotinylation exclusively during the liver stages, as shown for P.b. in vivo in infected mice and in vitro in HepG2 cell line [51].

P.f. liver stage antigen-1 (LSA-1) [52], when accumulated within the parasitophorous vacuole enveloping the cluster of developing liver merozoites, has been demonstrated to undergo PTM by host transglutaminase-2 (TG2); this enzyme catalyzes the unique isopeptide ε-(γ-glutamyl) lysine cross-bridge formation between glutamine and lysine residues of LSA-1 [53]. While the exact role of LSA-1 was not fully understood at the time, these results suggest that it undergoes extensive cross-linking, possibly contributing to the protection of the parasite during its development.

Proteolysis is an important protein modification in all stages of the parasite, including the liver stage. Site-2 proteases (S2P), belonging to the M50 family of metalloproteases, have been shown to be functional in *Plasmodium* for the activation of transcription factors, even redundantly, in the liver and blood stages [54].

Numerous interesting PTMs of host proteins are known and are elicited by parasites during their liver stage. Serine/threonine kinase 35 (STK35L1), induced by P.b. in both HepG2 cells and mouse liver, has been identified as a host kinase that upregulates numerous cell cycle genes. It plays an essential role in liver-stage parasite development and was proposed as a potential drug target against drug-resistant malaria [55]. The host enzyme 5′ AMP-activated protein kinase (AMPK), a central regulator of cellular energy metabolism, undergoes modulation by *P. chabaudi*. The infection results in decreased phosphorylation levels of AMPK during the liver stages of infection in mice. Furthermore, changes are observed in the hepatic mRNA and protein expression of crucial PTM enzymes and transcription factors, connected with AMPK and associated with lipid metabolism, leading to a lipogenic state [56]. Malarial liver-stage-induced innate immunity, involved in host protection and parasite growth control, was shown to be connected with autophagy activity in mouse models [57,58]. The parasitophorous vacuole membrane (PVM) can be often marked by autophagy marker proteins such as ubiquitin, LC3, and SQSTM1/p62, along with lysosomes, during a process similar to selective autophagy [57]. The expression of rodent hepatic autophagy-related genes (ATG) was decreased by ubiquitination, which was induced by parasite CSP and connected with the parasite protection and its survival inside hepatocytes even after IFN-gamma liver stimulation [58]. PVM building and transformations during the parasite development and liver schizont formation the lipidation of LC3 is also important [59].

Independently, the modulation of host cell sumoylation by the parasite facilitates efficient development of exoerythrocytic forms of P.b. and *Toxoplasma gondii* in the liver [60].

Modified proteins, reviewed in this section, their PTMs, and the enzymes involved are summarized in Table 2.

### 2.3. Asexual Forms in the Host (Erythrocytic Stage)

#### 2.3.1. Studies of Multiply PTMs

The clinically most significant phase of malaria infection, accompanied by symptomatic manifestations, is the asexual erythrocytic stage of *Plasmodium*, which continues in the host after parasite egress from the liver. Protein PTMs during the asexual intra-erythrocytic stage were analyzed in extensive studies on the P.f. parasite and separately for P.f.-infected erythrocytes [64]. Applying the tandem mass tag labeling, MS-based proteomics, and post-translational modification (PTM)-omics, numerous proteins were shown to be extensively modified [64]. The levels of six PTMs (phosphorylation, acetylation, crotonylation, 2-hydroxyisobutyrylation, N-glycosylation, and ubiquitination) were analyzed at six clusters, which reflect protein functionality and the parasite growth period. For P.f., 1518 modification sites were identified, which were matched to 848 proteins in the 6 PTM-omics. In host erythrocytes, 5034 modification sites were matched to 1924 proteins. Phosphorylation was the predominant modification in the P.f. proteome and occurred in proteins involved in RNA transport, mismatch repair, spliceosomes, ribosomes, the phosphatidylinositol (PI) signaling system, protein processing, metabolic regulation, and drag response. In P.f., acetylated proteins were primarily found in the nucleus and ribosome, playing roles in DNA binding, protein heterodimerization, and organelle organization. Ubiquitination in P.f. regarded proteins associated with translation, RNA transport, substance metabolism, response to oxidative stress, and cellular biosynthesis. N-glycosylated proteins were mainly involved in correct cellular localization and protein binding. The dynamics of described modifications during maturation of P.f. were reported. Cluster 1 (first 8 h after merozoite invasion) included 47 modification sites associated with parasite maturation, gene transcription, nucleotide binding, RNA transport, and mRNA protective surveillance. Histone acetylation, phosphorylation, and 2-hydroxyisobutyrylation were found to promote gene activation in merozoite growth. Cluster 2 (8–16 h) showed high levels of phosphorylation due to metabolic parasite transformation and growth. The overall PTM presence in Cluster 3 proteins was at a high level in the mature stage (24, 32, and 40 h) and were predominantly involved in the interplay with the host, as well as cell adhesion, and were mainly modified by phosphorylation. The overall PTM abundance of 38 proteins in Cluster 4 (trophozoites) increased gradually from 8 to 40 h and then decreased. Included in this cluster were the components of apical complex and rhoptry, histone binding, protein processing and export, protein-DNA complex assembly, members of the purine nucleoside diphosphate metabolic process, gluconeogenesis, and glycolysis [64]. Trophozoite stage parasites are generally described metabolically most active, exhibiting transcriptional enrichment in genes related to amino acids, tRNA, ncRNA, DNA, pyruvate, glycolytic, and carbohydrate metabolic processes [6,65]. Thus, PTMs are likely instrumental in the activation of these proteins. The abundance of PTMs in Cluster 5, with 40 modification sites, increased from 8 to 32 h and were stable from 32 to 48 h. This period is the stage of generating multiple nuclei and forming progeny cells, totally ready for egress. Placed in this cluster were the components of the nucleosome, inner membrane complex, and the pellicle (peripheral cytoskeletal structure). They mainly regulated histone binding, gluconeogenesis, glycolysis, pyruvate metabolism, magnesium binding, and actin-binding processes. Up to 13 histone acetylation modifications, as several sugar metabolic enzymes with 2-hydroxyisobutyrylation modifications, were identified in Cluster 5, with a particularly high abundance at the last three time points (32, 40, and 48 h). The overall protein PTM abundance in Cluster 6 included 27 modification sites, which increased, reaching the highest level at 48 h. Grouped in this cluster were the components of nucleosome and ribosome, the regulators of DNA activities, chromatin assembly, protein dimerization activity, biogenesis, and cellular component organization. Motor complex proteins were also detected in this cluster. Histones and their variants were modified by epigenetic modifications, and other proteins were modified by phosphorylation [64]. For more detailed information regarding the individual proteins and modifications detected in this study, please see the original paper [64].

#### 2.3.2. Phosphorylation and Dephosphorylation

Concluding from the aforementioned study [64] and other research, phosphorylation and dephosphorylation emerged as major protein PTMs, with kinases and phosphatases being more commonly studied enzymes in all stages of *Plasmodium*, including the asexual stage [10,11,12,13]. *Plasmodium* kinases are involved in cell proliferation and differentiation, cell-cycle control, parasite invasion and egress, and host–parasite interaction [7]. *Plasmodium* protein phosphatases are involved mostly in the same processes as kinases. They are clustered within the four major eukaryotic protein phosphatases families: Metallophosphatases (PPP), Serine/Threonine Phosphatases (PPM), Protein Tyrosine Phosphatases (PTP), and NLI Interacting Factor-like phosphatases (NIF) based on genomic database PlasmoDB. For both kinases and phosphatases, the differences between *Plasmodium* and other eukaryotic organisms are mainly substantial [7,66]. Thus, kinases, phosphatases, and related proteins were proposed as important targets for antimalarial therapy (see the Section 3).

Experimentally, phosphoproteome was applied to show the presence of phosphorylated proteins in the different stages of the parasite. The P.b. protein phosphatome was analyzed regarding parasite proliferation and differentiation [12]. The P.f. schizont phosphoproteome was experimentally performed [11] and revealed, among others, extensive phosphatidylinositol and cAMP-protein kinase A (PKA) signaling pathways. Most key enzymes in the inositol pathway were phosphorylated. A distinct phosphorylation pattern was described in proteins involved in merozoite egress and erythrocyte invasion. Additionally, cAMP-PKA signaling was involved in a wide range of processes, such as motility, invasion, and egress [11,67]. When proteomic analysis was applied together with in vitro kinase assay, three PKA substrates associated with the glideosome motor complex (GAP45, myosin A, CDPK1) were detected [11]. In another study, P.f. cAMP-PKA was shown to be involved in host erythrocyte anion channel regulation [68]. The review paper [69] summarizes the role of AMP-dependent protein kinase in *Plasmodium falciparum* development and invites future research to amplify the spectrum of antimalarial compounds using this kinase as a target.

The *Plasmodium* serine/threonine kinase PfPK7, found in different parasite forms in the vector and in the host, was abundantly characterized [70,71,72]. It is an “atypical” kinase because PfPK7 shows homology to mitogen-activated protein kinases (MAPK) kinases (MAPKK) in the regions of the C-terminal lobe of the kinase domain and to protein kinase A enzyme in the N-terminal region [70]. A quantitative phosphoproteomic study identified 3875 phosphorylation sites targeted by PfPK7 on 1047 proteins in schizonts [72]. Additionally, PfPK7 plays a crucial role in the melatonin transduction pathway, which is connected with the ubiquitin/proteasome system [71]. Another substrate, PfPK5 phosphorylates origin recognition complex subunit 1 (PfORC1), is involved in DNA replication and var gene regulation in the P.f. asexual stage [73]. Kinase PfPK4 also plays a crucial role in the erythrocytic cycle. Under environmental stress, it phosphorylates the regulatory serine 59 of *Plasmodium* eIF2α [74]. This implies that a translational shutdown is necessary at a certain point in the parasite cycle. Notably, PK4 activity not only halts global protein synthesis during the ontogeny of daughter merozoites in schizonts, but also affects mature P.b. gametocytes [74].

Calcium-dependent protein kinases (CDPKs) are involved in calcium signaling at different forms of the parasite [37]. Calcium signaling is an important messenger for the egress of the malaria parasite from the infected erythrocyte, gametogenesis, ookinete motility in the mosquito, and sporozoite invasion of mammalian hepatocytes. CDPKs are analyzed for all parasite stages in correspondent sections in this review, including the less studied calcium/calmodulin-dependent kinases CAMK [75].

Another important kinase, PfPKG, plays a central role in blood-stage invasion and schizogony [10,76], as in liver stage [45] and gametocytes [77] (see also the Section 2.2 and Section 2.4). P.f. glycogen synthase kinase-3 (PfGSK-3) was studied for phosphorylation sites, expression and intracellular localization in the erythrocytic stages, and its selective inhibitors. PfGSK-3 is mostly found at the trophozoite stage. PfGSK-3 is transported to the erythrocyte cytoplasm where it was detected in vesicle-like structures [78]. The PfGSK-3 selective inhibitors were proposed later [79,80] (see the Section 3).

The actomyosin motor complex of the glideosome drives the gliding motility of *Plasmodium* motile forms. Glideosome Associated Protein 45 (PfGAP45) is the crucial component of this complex, as it participates in the anchoring and functioning of the complex. In P.f. merozoites, it was demonstrated that PfGAP45 is phosphorylated in response to Phospholipase C (PLC) and calcium signaling. PfGAP45 is phosphorylated by the calcium-dependent enzymes Protein Kinase B (PfPKB) and Calcium-Dependent Protein Kinase 1 (PfCDPK1) at serines S89, 103, and 149. The Phospholipase C pathway affects the phosphorylation of serines S103 and 149. Importantly, the PfGAP45 phosphorylation at these sites was shown to be differentially regulated during parasite development [81]. Another study additionally shows that GAP45 and MTIP are phosphorylated by PfCDPK1 in schizonts [82]. The 3-phosphoinositide-dependent protein kinase 1 (PfPDK1) was shown as an essential upstream activator of protein kinase A in P.f. during erythrocytic growth [83]. In all erythrocytic stages, two nucleosome assembly proteins from P.f. with histone chaperone activities, PfNAPS and PfNAPL, were described. Only PfNAPL was phosphorylated with the plausible involvement of host casein kinase II [84]. Casein kinases PfCK1 and PfCK2 are also very active and important for the asexual proliferation of parasites [85,86,87,88]. In the review, dedicated to PfCK2, this enzyme was described in relation to its involvement in, among others, chromatin dynamics, phosphorylating, NAPS, histones, and acetylation lowers binding affinity proteins (ALBAs) [86]. Generally, histone phosphorylation serves as a crucial epigenetic indicator linked to various cellular eukaryotic activities such as chromosome condensation, DNA replication, and transcriptional regulation. As indicated above, plasmodia histones undergo phosphorylation as well [86,89,90]. Next, host erythrocytic casein kinase II (CKII) was shown to phosphorylate VARC domain of PfEMP1, which is important for cerebral and placental malaria and immune recognition by the host [91]. Cyclin-dependent kinases (CDK) and other kinases are key regulators of the cell cycle. P.f. cyclin-dependent kinase-like kinases (CLK) and their regulators are important during the asexual cycle of P.f. [92]. For example, PfPK6 was isolated and characterized in the trophozoite, schizont, and segmented stages as cyclin-dependent and mitogen-activated kinase [93]. P.f. cyclin-related proteins were studied in vitro in relation to their association with histone H1 kinase activity and their ability to activate PfPK5 [94,95]. PfCRK4 is a member of an Apicomplexa-specific kinase subfamily related to cyclin-dependent kinases (CDK). This group of kinases was studied for its importance in schizogony [96], and two CDKs, Pfmrk and PfPK5, were investigated for their involvement in cell cycle control and differentiation [97]. The families of NIMA (Never In Mitosis A) and Aurora *Plasmodium* mitotic kinases were characterized as essential in the asexual and sexual stages [98], and were also proposed as drug targets for antimalarials for inhibition parasite replication and transmission blocking. MAPK homologues map-1 and map-2 were shown as functional kinases in *Plasmodium* schizogony and gametogenesis [99,100,101], and parasite PfPK6 and PfPK7 have the regions of homology to MAPK [72,93].

FIKK protein kinases are exclusively present in the apicomplexan genre (the P.f. genome contains twenty-one FIKK kinase genes). FIKKs are localized in Maurer’s cleft, as detected during parasite development in erythrocytes [14,102]. Most of them are essential for parasite growth and have been found to be involved in the trafficking of parasite proteins to the erythrocyte membrane. They phosphorylate numerous host proteins and have an impact on erythrocyte deformability [14]. For example, P.f. FIKK9.1 is a monomeric serine-threonine protein kinase, essential for intra-erythrocytic growth. It was shown that the enzyme’s substrates are both parasite proteins and host erythrocyte cytoskeleton spectrin, ankyrin, and band-3 [103]. Using a targeted gene-knockout approach, the localization and FIKK functions were studied. FIKK9.1, FIKK10.1, and FIKK10.2 were shown to be exported into host erythrocytes, and for FIKK9.1 and FIKK10.1, the export was confirmed via Maurer’s clefts. FIKK3 was associated with rhoptries and FIKK9.5 was localized in the parasite nucleus [102]. One FIKK family protein kinase of the P.b. (PbMLFK) was shown to be expressed during the mosquito and liver parasite stages (mentioned in the Section 2.2), but always with strong involvement in parasite–host interactions in the erythrocyte stage, phosphorylating both parasite and host proteins [47]. Another example, RhopH3, a protein associated with the P.f. rhoptry complex, undergoes phosphorylation at serine 804 by PfCDPK1. This phosphorylation is essential for the invasion of the host erythrocytes by P.f. [104].

The in vitro study on the splicing-related protein kinase PfSRPK1 and its involvement in mRNA splicing confirmed its localization in the nucleus and identified splicing factor PfSR1 as its substrate [105].

Protein phosphatases (PP) are very important and largely characterized in computational studies. The analysis of the PlasmoDB database for identifying and classifying all PPs of *Plasmodium* was reported [66,106]. Experimentally, the essential role of serine/threonine protein phosphatase (PfPP1) was shown for the glucose metabolism and parasite DNA synthesis, thus regulating P.f. cell division in the erythrocyte [107]. Transcriptomes indicate PfPP1 as an essential P.f. and human host non-homolog protein [108].

A calcium-regulated protein phosphatase, calcineurin, when depleted in late-stage schizonts, has a critical impact on merozoite-to-erythrocyte attachment and invasion in vivo [109]. The importance of calcineurin in other processes along all stages of P.b. was shown [109]. One potential substrate for calcineurin is HSP90 [110]. AMA-1, mentioned above in the Section 2.1, is a micronemal protein expressed in the parasite erythrocyte stage and plays important role in the invasion of the parasite into host cells. Both PfAMA-1 and PvAMA-1 were shown to induce strong humoral and cellular responses, indicating them as promising vaccine candidates. Using bioinformatic tools, it was shown that PvAMA-1 had fifty-four potential phosphorylation sites and three acylation sites, as well as one transmembrane domain on its sequence. Targeting by vaccination may affect protein function and activity [111].

#### 2.3.3. Acetylation and Methylation

Much like phosphorylation, reversible protein acetylation and methylation control numerous vital cellular processes. The acetylation of hundreds of cytoplasmic and nuclear proteins, among them protein kinases, but mostly histones, in the blood-stage parasites of P.f. were reviewed [112]. Histones are acetylated in a number of lysine residues [113], and they are studied mostly in the erythrocyte stage, but the importance of their role in all other parasite stages is gaining recognition [114]. Developmental and stage-specific *Plasmodium* genes also depend on histone methylation [114,115]. Additionally, various histone deacetylases (HDACs), including PfSIR2, regulate the silencing of some *Plasmodium* spp. virulence genes, antigenic variation, gametocyte conversion [115], and rDNA-to-rRNA transcription [116]. Although extensively studied in recent years, the biological meaning of numerous acetylation and methylation events need to be further elucidated [114,117]. Some important examples are reported below.

The var gene family comprises 60 members, all of which encode the major adhesion surface molecule PfEMP1. Only one var gene is expressed at a time, with expression switching among the 60 different members. This diversity helps the parasite evade the host immune response by continuously altering the antigens presented on the surface of infected erythrocytes. The var locus that is transcriptionally active is associated with histone modifications such as histone H3 lysine 9 acetylation (H3K9ac) and H3K4 demethylation (H3K4me2 and H3K4me3). In contrast, silenced var genes exhibit a significant presence of the heterochromatin mark H3K9me3 [8,118]. The clonal inheritance of an active var locus persists over multiple asexual cycles. During each 48 h cycle, the transcription of the active var gene reaches its peak in the early ring stage. In the later stages, it is temporarily repressed, but remains poised for transcription in the next cycle by retaining enrichment in H3K4me2 [118]. Consequently, the interaction between activating and repressive histone marks establishes a lasting signature that facilitates the propagation of the active and silenced states of var genes through cell division. Genome-wide mapping has revealed a distinct correlation between the H3K9me3 mark and silent-variant gene families, located in subtelomeric regions. Then, H3K4me3 and H3K9ac were shown as activation marks with a wide distribution in the P.f. genome, with peaked concentrations at promoter regions [118]. H3K9ac appears to be linked to the expression of mRNA throughout the erythrocytic stage. H3K4me3 is deposited in a stage-specific way and marks genes active at a single stage during the erythrocytic cycle, identical to the poised var gene [8,118].

Regulators of histone PTMs are widely presented in *Plasmodium* ssp. genomes; some are functionally characterized, others are still putative. Of all the histone acetyltransferases, PfGCN5 and PfMYST are the best characterized and are supposed to be essential for intraerythrocytic parasite forms [119,120]. The most extensively studied P.f. deacetylases HDACs include PfSIR2A, PfSIR2B, and the class III NAD+-dependent HDAC sirtuins. Interestingly, PfSIR2A and PfSIR2B work in synergy with perinuclear acetylated histones in order to silence various groups of var genes. PfSIR2A targets include H3K14ac, H3K9ac, and H4K16ac [8]. PfHDA2 is involved in the control of the var genes’ expressions and the master regulator of sexual development transcription factor PfAP2-G [115]. Numerous proteins, such as 20S proteasome beta subunit, 14-3-3 proteins, 6-phosphofructokinase, actin I, acetyl-CoA synthetase, elongation factors 1 and 2 (both alpha and beta), and enolase, were shown as acetylation substrates during the P.f. asexual stage [112]. The methylation of histones and non-histone proteins is catalyzed by histone arginine methyltransferases (PRMTs, as PfPRMT1 and PfPRMT5), histone lysine methyltransferases (HKMTs), and coactivator-associated arginine methyltransferase 1 (PfCARM1) [9,121]. PfPRMT1 was detected in the nucleus and cytoplasm of asexual parasites. It methylates R3 of histone H4, as well as non-histone proteins [122]. In another study, ten SET domain-containing HKMTs were identified, with five of them being crucial for blood-stage proliferation. PfSET10 methylates H3K4 and co-localizes during post-ring stages with the active var gene, meaning the potential involvement of PfSET10 in the active var gene remaining in a poised state during the parasite mature stage [123]. PfSET2, instead, methylates histone H3K36 and is associated with var multigene family repressive chromatin [124,125]. PfSET7, which was mentioned in the Section 2.1, was shown to be an essential parasite enzyme, not only in the transmission and liver stages, but also in the erythrocytic stage [19]. Analyzing non-histone methylated proteins, the proteomic study has shown that more than 10% of the P.f. proteins undergo lysine methylation [126]. Among these, numerous surface and secretory proteins were reported, including rhoptry neck protein 3 (RON3), rhoptry protein 14 (ROP14), trophozoite excretory protein (TEX1), rifin, 6-cysteine protein (p12), and PfEMP1, which have been identified as lysine methylated proteins. Notably, two inner membrane complex proteins (IMCs), specifically IMC 1g and 1c, exhibit extensive methylation, with 16 and 15 lysine methylation sites, respectively. Many of these lysine-methylated proteins, particularly those located on the parasite surface and involved in the gliding motility of merozoites during invasion, also undergo phosphorylation [126]. Another proteomic study focused on identifying arginine-methylated proteins in the blood stages of P.f. [121]. This study identified proteins involved in RNA binding and translation, such as ALBA1, rRNA 2′-O-methyltransferase fibrillarin (NOP1), DEAD/DEAH helicase, eukaryotic translation initiation factor 2c, and 40S ribosomal proteins S4, S5, S6, S7, S10, S15A, S19, and S21. Arginine methylation is also used by P. f. for chromatin organization, as seen in two histones and one nucleosome assembly protein (NAPL). Additionally, proteins involved in DNA replication (DNA replication licensing factors MCM4, 5, 6, and 7) and DNA repair (RAD50 and DNA excision repair protein SNF2 helicase) were methylated. Furthermore, arginine methylation of Ras-related proteins (vesicle transport regulators RAB1-B, RAB11-A, and RAB18) and AP-1 subunit gamma from the adaptor protein (AP) complex play a crucial role in intracellular protein trafficking [121]. Small nuclear ribonucleoproteins (snRNPs) are RNA-protein complexes that contain specific snRNAs, Sm core proteins, and several snRNP-specific proteins. PfSmD1 was shown to be methylated, likely by PfPRMT, which was necessary for its interaction with Tudor domain-containing proteins, facilitating the assembly of the spliceosomal core complex [127]. Additionally, P.f. has 3 histone demethylase orthologues of lysine-specific demethylases (LSD1) and Jumonji-C histone demethylases (JHDM) [128,129].

#### 2.3.4. Protein Cleavage and Processing

Protein cleavage and processing are pivotal for parasite life. For survival and development in the erythrocyte, parasites need to digest enormous quantities of host proteins, mainly hemoglobin. Additionally, parasites must also cleave and process their own proteins. *Plasmodium* spp. express a series of proteolytic enzymes, of which cysteine, serine, aspartic, and metallo-proteases are the best known and relatively well studied [7,8]. Some are shown to be essential for parasite survival, some are redundant or replaceable, and some are still putative.

Cysteine proteases activity and their regulation (for example, by the inhibitors of cysteine proteases, ICP) are important in the blood stage of human and rodent malaria [130,131,132,133], in the liver stage in the host for parasite–hepatocyte intertalk during exoerythrocytic merozoite release [133], and in *Plasmodium* sporozoite egress from oocysts [29] (see also the Section 2.5). More specifically, cysteine proteases falcipain-2 and -3 are principally involved in hemoglobin digestion [130,131,134], and falcipain-1 plays a major role in merozoite invasion [130,131]. P.f. DPAP3 activity was shown to be important for the efficient erythrocyte invasion by merozoites [135]. Note, whereas SERA-4, -5, -6 cysteine proteases are important in the erythrocytic stage, SERA-8 is pivotal for sporozoite development (see the Section 2.1) [7]. Calcium-dependent cysteine protease calpain (P.f.-calpain) has an important role in the parasite calcium modulation, membrane degradation and parasite development, mostly at the P.f. trophozoite stage [136].

Serine proteases with the representative sheddases subtilisin-1, -2, and -3 and subtilisin-like proteases are important for protein processing, the regulation of erythrocyte invasion, and parasite egress [7,137]. ROM serine proteases have been demonstrated to play a crucial role in protein processing associated with invasion, the formation of the parasitophorous vacuole, and the shedding of adhesins [7,138,139]. Biochemical analysis of cleaved substrates suggests a role of certain ROMs in all invasive stages, both in the host and in the vector [140].

Among the aspartic proteases, plasmepsins are the best studied and are the first proteases shown to initiate hemoglobin degradation and cleavage of denatured globin [130]. Further studies revealed a wide range of additional functions of plasmepsins, including protein export and PfEMP1 exposure [141], involvement in merozoite egress [142], and interaction with subtilisin-like protease 1 [137]. Histo-aspartic protease (HAP), a food vacuole protease very similar to plasmepsins, was described and structurally studied [130,143].

P.f. metalloprotease falcilysin was shown to digest hemoglobin in the acidic food vacuole in in vitro tests after its recombinant expression [144]. Aminopeptidase P from P.f. (PfAPP) was characterized and shown to be important for final hemoglobin digestion [145].

In addition to host hemoglobin-degradation for nutrition, the parasite also drastically remodels other host erythrocyte proteins, which is necessary for its own development, multiplication, and egress. P.f. signal peptide peptidase (PfSPP), which cleaves off signal peptides during the export of functional proteins in parasites, was shown to be essential for the invasion and growth of the parasite in host erythrocytes: it binds to the erythrocyte band 3 anion exchanger and promotes merozoite invasion [146]. The M18 aspartyl aminopeptidase of P.f., over the role in parasite protein processing, was shown to bind to human erythrocyte spectrin in vitro [147].

#### 2.3.5. Nitrosylation

Nitric oxide signaling and nitric oxide synthase (NOS) expression are largely involved in both host and vector response during interaction with the parasite [148,149,150,151]. Note that the majority of studies were conducted with rodent models, even though the human and mouse immune responses have different iNOS regulations [152]. The nitrosylation PTM is rarely described in malaria. S-nitrosylation in P.f. proteins was studied using the biotin-switch approach coupled to mass spectrometry. A total of 319 potential targets of S-nitrosylation were identified, distributed across various cellular pathways. Glycolysis in the parasite emerged as a significant pathway target, with glyceraldehyde-3-phosphate dehydrogenase being notably inhibited by S-nitrosylation in its active site cysteine. Furthermore, it was shown that P.f. thioredoxin 1 (PfTrx1) can be S-nitrosylated at Cys43 outside the active site [153]. These results indicate that S-nitrosylation might be considered as target for antimalarial therapy (see the Section 3).

#### 2.3.6. Glycosylation

Glycosylation started to be intensively studied in *Plasmodium* in the 80s-90s when the importance of N-linked glycoproteins for P.f. development in the erythrocyte was suggested [154,155]. Protein O- and C-glycosylation were initially highly discussed and doubted for *Plasmodium*, while well characterized for *Toxoplasma* [156,157]. Recently, the glycosylation PTMs were confirmed for *Plasmodium* (e.g., CSP glycosylation, see the Section 2.1), in particular in proteins containing TSR domains, modified by O-fucosylation and C-mannosylation [23,157]. Examples of N- and O-linked glycoproteins are MSP1 and MSP2 [158], and the importance for sporozoite and merozoite development and functionality must be emphasized.

#### 2.3.7. Glutathionylation

S-glutathionylation PTM serves mainly to protect the parasite from oxidative stress and is controlled by the specific enzymatic system. A total of 493 targets for protein S-glutathionylation were identified in P.f. Fifteen of the glutathione-modified proteins were functionally highly important: thioredoxinc, thioredoxin reductase, thioredoxin peroxidase 1, glutathione S-transferase, glutathione reductase, mitochondrial dihydrolipoamide dehydrogenase, plasmoredoxin, glutamate dehydrogenase 1, ornithine δ-aminotransferase, glyoxalase I and II, glyceraldehyde 3-phosphate dehydrogenase (GAPDH), pyruvate kinase (PK), lactate dehydrogenase, and phosphoglycerate mutase. It was shown that P.f. ornithine δ-aminotransferase, GAPDH, and PK are reversibly inhibited by S-glutathionylation. P.f. enzymes thioredoxin 1, glutaredoxin 1, and plasmoredoxin were shown to efficiently catalyze protein deglutathionylation [159].

#### 2.3.8. Lipidation

Protein modifications by lipids regulate mostly the interaction of proteins with membranes or affect the functionality of selected proteins. The forms of lipid modifications vary significantly in their occurrence, predictability, and regulatory functions. For prenylation (isoprenyl modification), the binding site is the free thiol of a cysteine side chain at or near the protein C-terminus, and myristoylation regards the addition of a 14-carbon saturated fatty acid and myristic acid, as well as to the N-terminal glycine residue. Thus, the location of these two PTMs is relatively predictable and sequence-directed. Instead, the location of (i) palmitoylation (cysteine residues in S-palmitoylation, serine, and threonine residues in O-palmitoylation), (ii) lipoperoxidation product conjugation (specific or non-specific lysine, cysteine, and histidine residues of proteins), or (iii) glycosylphosphatidylinositol anchor (GPI) binding on the carboxyl terminus of the protein cannot be predicted solely based on the primary amino acid sequence. Myristoylation predominantly occurs co-translationally in proteins with a Met-Gly motif at the N-terminus, although it can also happen post-translationally at internal glycine residues. In contrast, prenylation, palmitoylation, and the addition of GPI anchors occur exclusively post-translationally. Proteins can undergo multiple lipid modifications, with concurrent myristoylation and palmitoylation being common. Importantly, the addition of GPI anchors and palmitic acid is largely reversible, allowing for dynamic regulation of protein location and activity. Conversely, myristoylation, prenylation, and lipoperoxidation product conjugation are generally considered irreversible [8]. N-myristoyltransferase (PfNMT, known also as glycylpeptide N-tetradecanoyltransferase) was shown to be important in different phases of the erythrocyte P.f. stage, as the PfNMT inhibitor blocks the parasite development, egress, and invasion [160,161]. Numerous PfNMT substrate proteins were identified [161], e.g., GAP45, a component of the invasion motor complex, essential for erythrocyte invasion by merozoites [160]. In other studies, the differential effect of lipid modifications was shown for PfGAP45, which underwent both myristoylation and palmitoylation [8,162,163]. Another example of a P.f. functionally modified protein is the armadillo repeats-only (ARO) protein that owns both myristoylation and palmitoylation motifs [164]. It was shown to interact with PfAIP and to be important for the rhoptry-related mechanism of parasite invasion [165]. During the blood stage of *Plasmodium* spp., there are approximately a few dozen myristoylated proteins and a similar quantity of proteins anchored with GPI [8]. Two of the most important GPI-anchored proteins are PfMSP1 and PfMSP2, essential for the successful egress and invasion of human erythrocytes by merozoites [166]. They have long been known to be targeted by the host immune system [167]. Palmitoylated proteins are more numerous, with an estimated count of more than 400 putative proteins [161,163]. For example, the transporter proteins chloroquine resistance transporter PfCRT and PfMDR1 are important palmitoylated proteins located in the lipid membrane of food vacuole [168]. Note that PfCRT also undergoes phosphorylation, plausibly mediated by CDPK, as well as potentially ubiquitination, and is considered to be a promising target for therapy [163,168]. Palmitoylation and palmitoyl-transferases in *Plasmodium* spp., with their metabolic impact, were reviewed [161,169]. Of particular interest is DHHC7, which localizes to rhoptry organelles in parasites of different species, including P.f., and appears to be essential for parasite invasion [170]. In P. yoelii schizonts, DHHC2 was shown to palmitoylate CDPK1 and GAP45 [171].

Protein prenylation (lipid PTM) is particularly interesting for drug target research, as prenyltransferases (PTases) are involved in many physiological and pathological processes in various species of eukaryotes, including malaria parasites [161,172,173]. Prenylation is catalyzed by the soluble PTases farnesyltransferase (FT or FTase) and geranylgeranyltransferase (GGT). For their essential role in P.f., together with NMT, these enzymes are considered promising drug targets [161,174,175]. Global proteomic analysis of prenylated proteins in blood-stage P.f. using an alkyne-modified isoprenoid analogue revealed thirteen prenylated proteins: among them were Rab GTPases and the proteins involved in parasite membrane trafficking [172]. For example, Rab5 was associated with the food vacuole membrane, where it plays a pivotal role in the survival of the parasite [176]. The SNARE (SNAP Receptors) family protein PfYkt6p, identified in *P. falciparum*, was found to undergo both prenylation and geranylgeranylation, which regulate PfYkt6p transport [177,178]. The FYVE-containing coiled-coil protein (FCP) is prenylated, conserved across *Plasmodium* spp., and predominantly resides within the parasite’s food vacuole, playing important roles in vesicle targeting. The use of farnesyltransferase inhibitors (e.g., THQ class inhibitor BMS-386914) abolished FCP prenylation and led to its cytosolic mislocalization [9,179]. Interestingly, the activity of protein prenylation enzymes PTases is modulated, in turn, by phosphorylation [173]. Thus, P.f. PTases regulation at the genetic and epigenetic levels is finely tuned and it could be of therapeutic interest. In another study, farnesylation was investigated using purified protein farnesyltransferase from P.f. in order to identify its targeting sites [180]. Prenylation proteins specific to *Plasmodium* spp. differentiation were notably abundant during the transitions from the trophozoite to schizont and from the schizont to ring stages [180].

#### 2.3.9. Lipoxidation

Lipid oxidation products, such as malondialdehyde (MDA), acrolein, and 4-hydroxynonenal (4-HNE) are produced both enzymatically and non-enzymatically upon various oxidation processes, both in *Plasmodium* and in the host [181,182,183,184]. They frequently damage modified proteins, though parasite and host cells are able to discard or replace affected proteins through their defense mechanisms [185,186].

#### 2.3.10. Autophagy, Ubiquitination

The autophagy system in parasites also seems to play an important role in parasite development. Complex interactions based on phosphorylation, lipidation, and ubiquitination PTMs are involved [187]. Autophagy-related proteins (Atgs) are expressed during all parasite stages, mostly during the host erythrocyte and vector stages. Studies suggest that Atg8 and its post-translational modifications play a crucial role in apicoplast maintenance, heme degradation within the food vacuole, and potentially in the trafficking of proteins or organelles outside the parasite membrane. Additionally, autophagy might be involved in programmed cell death during drug treatment or serve as a selective mechanism to control parasite load [187].

Ubiquitination is an important PTM, not only in the sporozoites, as mentioned above (see the Section 2.1), but also in the erythrocyte parasite stage. The ubiquitin encoding genes UbL40, UbS27a, and pUb are expressed in concomitance with the ubiquitinylating enzymes of the endoplasmic reticulum-associated protein degradation (ERAD) enzyme system: the ubiquitin-activating E1, ubiquitin-conjugating E2, and ubiquitin E3 ligase. Ubiquitination, de-ubiquitination, and ubiquitination-similar protein tagging are actively used by the parasite, mostly in processes that regulate parasite development and cell cycle [34,188,189]. Like Nedd8 (structurally similar to ubiquitin) and all ubiquitin-like proteins (UBLs), SUMO (small ubiquitin-like modifier) is nearly identical to ubiquitin in overall structural fold, but is quite different in both amino acid sequence and function in *Plasmodium* [188,190]. Numerous ubiquitination-targeted functional proteins were identified. A mass spectrometry study identified the high level of ubiquitination of the ring-exported protein-1 (REX1), a P.f. protein located in Maurer’s clefts and essential for parasite nutrient import in the trophozoite stage [191]. Moreover, during the erythrocytic stage of *P. falciparum*, it has been found that a major subunit of RNA polymerase II (in trophozoites), two ubiquitin ligases (in trophozoites), E2 ubiquitin-conjugating enzyme (in both trophozoites and schizonts), and ApiAP2 transcription factor (in schizonts) are conjugated to ubiquitin [192]. As the ubiquitin conjugation with ubiquitin ligases and E2 ubiquitin-conjugating enzyme seem to be part of their enzymatic activity, RNA polymerase and ApiAP2 modifications are important for parasite development [192].

For de-ubiquitination, P.f. deubiquitinase ubiquitin C-terminal hydrolase L3 (PfUCHL3) was shown to be as important for the asexual *P. falciparum* blood stages [35] as for the sporozoite stage (see the Section 2.1). Nedd8 was hydrolyzed by UCH proteases in *Plasmodium* spp. [193]. Importantly, in order to illustrate interspecies metabolic differences and underscore the significance of careful protein kinase selection as drug targets, we underline here the protein kinase 9 (PK9) and ubiquitin-conjugating enzyme (Ubc13) as examples. While both are functionally significant for P.f. blood [194] and liver stages [46], the study on P.b. reveals the indispensable role of Ubc13, without necessity of phosphorylation by protein kinase throughout the parasite development in the host [195].

#### 2.3.11. Biotinylation

Biotinylation in *Plasmodium* spp. is not a frequent protein post-translational modification, as only the enzyme ACC can be modified by biotin in both the blood and liver stages of the parasite [51].

#### 2.3.12. Hemozoin-Related PTMs

Malarial pigment hemozoin (HZ) crystallization helps to protect the parasite from the reactive free heme released during hemoglobin degradation by the parasite. Couples of heme molecules are assembled to a crystal structure by hydrogen bonds. HZ is accumulated in parasite food vacuole as brownish crystals, visible by optical microscopy and quantifiable by numerous methods, including polarizing light microscopy [196], luminescence methods [197,198], and nuclear magnetic resonance [199,200,201]. The close contact of the growing or mature natural HZ crystal with unsaturated fatty acids results in lipoperoxidation. Chemically instable lipoperoxides decompose to biological active lipoperoxidation end-products, like 4-hydroxynonenal (4-HNE), which is produced from the arachidonic acid [202] of closely located lipid membranes. The 4-HNE is able to modify proteins by Michael adduction or Schiff-base formation with lysine, histidine, and cysteine residues [183,202,203,204]. This is considered lipoxidation protein PTM [205]. Though potentially dangerous for the growing parasite organism, 4-HNE formation, together with physiological levels of oxidative challenge [206,207,208,209], is equilibrated by the antioxidant defense system, permitting regular parasite growth and development [185,186,210]. However, a shift towards excessive oxidative stress could be dangerous to the parasite [211,212], a vulnerability that could be exploited by antimalarials [211,213].

During reinfection in host blood cells, when mature merozoites escape from resident erythrocyte, after almost all hemoglobin and the erythrocyte membrane were degraded, HZ is expulsed in circulation. Expelled HZ is enveloped in food vacuole (called “residual body” by some authors) with (i) attached remnant parasite molecules and (ii) rapidly acquired host plasma molecules [207,214]. This complex, often called “natural HZ”, is recognized and avidly phagocytosed by immune cells in both patient circulation and in laboratory model systems [214,215,216,217]. The beta-hematin core of HZ is not digestible by phagocytes [197,215]. Thus, the parasite product HZ remains active inside the host even after the vital parasites are cleared, exerting its action through post-translational protein modifications in the host cells. Host fibrinogen, stably bound to HZ, rapidly activates monocytes via TLR-4 and CD11b/CD18-integrin during HZ recognition and engulfment [214]. Oxidative burst, provoked by this phagocytosis, is the initial step of protein oxidation and lipid peroxidation in host immune cells [183,214]. HZ remains undigested in any phagocytosing or -derived cells: undifferentiated monocytes, granulocytes, differentiated dendritic cells (DC), and resident macrophages [215,216,218,219,220,221]. In these cells, the long-term process of lipid peroxidation persists. Lipoperoxidation end-product 4-HNE is slowly and continuously shed, provoking the adduction with functionally important proteins in immune cells [222,223,224]. This process leads to immunosuppression [218,219,225,226,227] and dyserythropoiesis [228,229] in the host.

Experimental approaches revealed HZ-elicited 4-HNE binding to actin, coronin 1A, lamin A/C, heterogeneous nuclear ribonucleoprotein H, alpha-enolase, trioseisomerase [222], protein kinase C (PKC) [230], GM-CSF-receptor [223], and cytochrome P450 CYP4F11 [224] in human primary monocytes or monocyte-derived dendritic cells. 4-HNE modifications of actin and coronin caused impaired cell motility and phagocytosis [222], modifications of PKC led to impaired phagocytosis and oxidative burst [230], modifications of GM-CSF receptor caused impaired differentiation/maturation of dendritic cells [223], and modifications of CYP4F11 monooxygenase resulted in changes in hydroxy-PUFA metabolism [224]. Abundant 4-HNE PTMs were found to be localized to the external membrane of erythrocytes in malaria patients [208,209], which likely originate from parasitized erythrocytes, frequently forming rosettes. This was concluded from experimental data obtained with co-cultured infected with non-infected erythrocytes [208].

Modified proteins of asexual stage parasites, their PTMs, and the enzymes involved are summarized in Table 3.

### 2.4. Sexual Forms in the Host (Gametocytes)

The sexual form of *Plasmodium* initiates with gametocytes, induced and developed from the asexual forms in the host erythrocyte, through mechanisms that are not yet fully understood [244]. Numerous metabolic pathways are unique to gametocytes, such as exflagellation-related events or exposure of new adhesive proteins [244,245,246]. Other processes, instead, remain similar to the asexual forms of parasites. For example, remodeling of some host protein or hemozoin formation in gametocytes are similar to asexual stage parasites [244,247,248].

Proteome analysis of separated male and female P.b. gametocytes was performed [249]. The male proteome comprised 36% (236 out of 650) male-specific proteins, while the female proteome consisted of 19% (101 out of 541) female-specific proteins. The male gametocyte has the most distinct proteome compared to other *Plasmodium* life-cycle stages, featuring numerous proteins involved in flagella-based motility and rapid genome replication. Gender-specific protein kinases and phosphatases, such as the male-specific mitogen-activated protein kinase 2 (MAP2) and the female-specific NIMA-associated kinase (NEK4), were identified [249], underpinning phosphorylation as important PTM for the regulation of vital processes in the sexual stages. Later, a transcriptome analysis of male and female *P. falciparum* gametocytes was combined with a comprehensive proteome analysis [250]. In male gametocytes, there was an enrichment of proteins involved in the formation of flagellated gametes, including those related to chromatin organization, DNA replication, and axonemal formation. Female gametocytes were enriched in proteins necessary for zygote formation and various post-fertilization functions, such as lipid, protein, and energy metabolism. Numerous proteins were identified as overexpressed in female vs. male parasites, including NIMA-related kinase 2 and 4 (NEK2 and NEK4) [250]. Others were repressed, such as MAP2, CDPK4, SRPK1, and NEK1 protein kinase [250]. Using CRISPR/Cas9-based gene editing, it was shown that PfCK2α casein kinase catalytic subunit is localized in the nucleus and cytoplasm in asexual and sexual parasites, and is essential for the development of both stages [88]. PfPKG, an essential protein kinase in all parasite stages, plays a crucial role in gametocytes as well [77]. Given its significance, targeting PfPKG could be a valuable strategy in antimalarial therapy (see the Section 3). Another kinase, P.f. glycerol kinase (PfGK), which does not perform the phosphorylation in proteins, was described as a gametocyte female-specific enzyme [250]. This enzyme was utilized in an RT-qPCR assay for distinguishing P.f. male and female gametocytes in Burkina Faso patient blood samples [251]. Cyclin-dependent kinases and their homologues were shown to be important for gametogenesis. For example, the CRK5 enzyme is critical for male gametogenesis [240,252]. Also, MAP kinases (e.g., MAP2) were shown to regulate male gametogenesis and the transmission of the malaria parasite P.b. [100] and P.f. [101]. An example of protein substrate for phosphorylation is Pfg27. The localization of this protein in the gametocyte nucleus was shown. Through binding to RNA, endogenous Pfg27 formed oligomeric complexes in developing gametocytes, which were affected by phosphorylation at Ser32 and Thr208 of Pfg27 [253].

Specific proteolytic processing seems to be necessary for regular gametocyte function, too. Gametocyte egress-important protein, Pfg377 [254] has been demonstrated to co-localize with the proteases subtilisin 2 (PfSUB2) and dipeptidyl aminopeptidase 2 (PfDPAP2) in the osmiophilic body, and is supposed to be enzymatically processed [255]. Numerous PfGEXPs (*P. falciparum* gametocyte-exported proteins) were identified, and three of them were found by mass spectrometry to undergo N-terminal processing and N-acetylation at a conserved leucine residue within the *Plasmodium* export element pentamotif in the early stages in gametocytes [256]. Recently, quantitative mass spectrometry analysis of proteins expressed in purified P.f. gametocytes upon induction of gametogenesis was reported. Among the four most upregulated proteins, plasmepsin X (PMX) was detected [257]. Similarly, plasmepsin V (PMV) is essential for gametogenesis, as gametocyte generation and transmission to mosquitoes were shown to be blocked by PMV inhibiting [258,259].

Specific gametocyte histone acetylation–deacetylation as a regulator of gene expression during human-to-mosquito transmission was described [114,115,260]. For example, deacetylase PfHDA2, important for the erythrocyte stage and mentioned in the Section 2.3, is involved in the regulation of the transcription factor PfAP2-G, the activator of gametocyte genes [115,261]. Recently, mass spectrometric analysis of histones from the early, middle, and late stages of gametocytes identified 457 unique histone peptides with 90 post-translational modifications, half of which were novel [246]. A high abundance of acetylation and methylation in H2A, H2A.Z, H2B, H2B.Z, H3, H3.3, and H4, and the ubiquitination of H3BK112 were detected [246].

The importance of palmitoyltransferase PfDHHC9 was shown in gametocytes. While its disruption did not affect the growth of blood-stage parasites, this decreased the formation of gametocytes, suggesting that the protein could be targeted to block transmission [262].

A recent review paper compared all PTMs related to gametocyte growth, with PTMs from erythrocyte-stage parasites [263]. Most of them are reported below in the Section 2.5. For example, for the early gametocytes, protein phosphatase PfPPM2 was reported to be marked by seven distinct PTMs, including the following major modifications: acetylation, methylation, nitrosylation, glutathionylation, ubiquitination, and Palmitoylation [263]. Note, PbPPM2 was also shown to participate in the regulation of *Plasmodium* sex allocation [12].

Although several protein kinases and other PTM-related enzymes were reported in the proteomic and functional studies, as mentioned above, numerous highly specific gametocyte-associated proteins are mostly surface proteins with still largely unknown functions. To our knowledge, few PTMs have been detected in these proteins so far. Thus, PTMs must be identified both for the already-described and new metabolic processes in gametocytes in order to understand their role and exploit them for drug targeting.

Modified proteins, reviewed in this section, their PTMs, and the enzymes involved are summarized in Table 4.

### 2.5. Sexual Forms in the Vector and Sporozoite Formation

Ingested by *Anopheles* mosquito, *Plasmodium* undergoes further sexual development such as mating and multiplication, finally resulting in the sporozoite form. Sporozoites in mosquito salivary glands are ready to be injected with saliva into the next host during the mosquito bite [6,263,265].

The most recent review delineates the known mechanisms engaged in the sexual stage development of P.f. in both the human host and the mosquito vector [266], and the involvement of the wide network of PTMs in this process.

Stage-specific kinases and phosphatases in both male and female gametogenesis are under sustained attention and were reviewed [12,267]. In a proteomic study, the phosphorylation regulation of glycolysis, RNA translation, protein synthesis, tubulin-associated cytoskeleton dynamics, and environmental stress response was shown during P.b. gamete formation [268]. In another project, protein phosphorylation during P.b. gametogenesis was studied in a serum-free medium in vitro, employing bi-dimensional electrophoresis (2-DE), immunoblotting (IB), and specific antibodies in order to phosphorylated serine, tyrosine, and threonine [269]. Approximately 75 proteins were phosphorylated, with 23 proteins identified through mass spectrometry. These proteins included heat shock proteins, components of the cytoskeleton, and proteins involved in DNA synthesis and signaling pathways, among others. The phosphorylation sites of six identified proteins—WD40 repeat protein MSI1, actin-1, HSP70, enolase, and two isoforms of the large subunit of ribonucleoside reductase—were further analyzed using titanium dioxide phosphopeptide enrichment and tandem mass spectrometry [269].

P.b. protein kinases at all life stages were analyzed, revealing the redundancy of 23 protein kinases for asexual development and the importance of another set of kinases in parasite sexual development and sporogony in *Anopheles stephensi* mosquitoes. Roles for SR protein kinase (SRPK) in microgamete formation, the conserved regulator of clathrin uncoating (GAK) in ookinete formation, and the probable regulator of energy metabolism (SNF1/KIN) in the development of sporozoites, were identified [38]. Protein kinase (PbMLFK), mentioned above for the liver parasite stage, was also shown to be functionally important in the transformation of oocytes in sporozoites [47]. The importance of phosphatase activity was shown for sexual forms: P.b. stage-specific depletion of calcineurin (serine-threonine specific calcium-calmodulin-activated protein phosphatase), showed its role in gamete development and fertilization, ookinete-to-oocyst, and the subsequent sporozoite-to-liver stage passage [109].

Note, three proteomic studies on P.b. oocysts were performed [270,271,272] and one in P.f. sporozoite maturation forms [271]. Proteins strongly involved in the translation process, sporozoite cytoskeleton organization, mitochondrial activity, and proteolysis, as well as in the maturation and infectivity of sporozoites, were identified. Some proteins, like CSP, with known functions, and numerous proteins with not fully known functions (e.g., putative secreted ookinete protein PSOP1 or ookinete surface proteins P25 and P28), were detected. However, just a few proteins related to PTMs were found, such as casein kinases, ATP-dependent protease, and 26S protease regulatory subunits [272].

Subtilisin proteases have been described above for their importance in the host stages of parasites. Furthermore, SUB2 was found in osmophilic bodies of gametocytes and was shown to be secreted into the mosquito midgut epithelial cells for the structural modification of the vector cell cytoskeletal network [273,274]. P.f. subtilisin-like ookinete protein SOPT has an important and conserved role (as serine protease or as pseudoprotease) in ookinete development of the midgut of *Anopheles stephensi* [273]. The autophagy-related proteases Atgs, which are expressed in all parasite stages and are involved in the programmed cell death, were reviewed for their importance in the trafficking of proteins or organelles. The review also highlighted Atgs’ significance in the sexual development of parasites in the vector [187].

Another review on gametocytes computationally compares numerous PTMs (intentionally excluding phosphorylation) between asexual erythrocyte and sexual gametocyte developmental stages in the host and in the mosquito [263]. The authors reported that between 25% and 50% of proteins showing post-translational modifications in asexual stages are expressed in gametocytes [263].

The Aurora family of kinases (ARK) plays a pivotal role in coordinating chromosome segregation and cytokinesis throughout cell division, tightly regulated in space and time by specific protein scaffolds. Investigating the involvement of ARK2 in P.b. sexual development, mass spectrometry, super-resolution microscopy, and live-cell fluorescence imaging were applied. This approach revealed potential ARK2 substrates including the Myosin-K, MISFIT, and microtubule-interacting protein EB1 [275].

Histone-associated proteins, such as heterochromatin protein 1 (PfHP1), are involved in gametocyte differentiation and erythrocyte invasion. PfHP1 is recruited by the repressive epigenetic mark H3K9me3. Its binding regulates the formation of heterochromatin. Conversely, phosphorylation of histone H3 at serine 10 (H3S10ph) impedes HP1 binding. It has been proposed that Aurora B kinase-mediated H3 phosphorylation is part of a “methyl/phos switch” mechanism that displaces HP1 and potentially other proteins from heterochromatin [266]. Additionally, a metallo-dependent protein phosphatase, PPM1, also plays a significant role in P.b. male gametocyte exflagellation: the parasite mutant without PPM1 formed morphologically normal gametocytes, produced macrogametes, and expressed the activation marker P28, but did not produce any ookinetes, and the exflagellation was completely blocked [12]. Reverse genetics studies have shown that the phosphatase PPKL (protein phosphatase with kelch-like domains) is crucial during ookinete differentiation. It plays a role in the develop of ookinete motility, pellicle morphology and integrity, and ookinete polarity [276].

Gene activation correlates with histone activation marks, such as acetylations H3K9ac, H3K14ac H3K18ac, H3K27ac, H3K56ac, H4K8ac, H4K16ac, tetra-acetylation H4ac4, methylation H4K20me, trimethylation H3K4me3, and the histone variant H2A.Z [266,277]. On the contrary, gene repression correlates with specific histone methylation and acetylation patterns, such as trimethylations H3K9me3, H3K36me3, and H4K20me3 [266,277]. The progression of gametocyte development from stage I to stage V is marked by euchromatic post-translational modifications and repressive methylation marks on histone 3. In the early gametocyte stages (I to III), modifications, including H3K9me3, H3K27me2, H3K27me3, H3K36me2, H3K37me1, H3R17me1, and H3R17me2, were detected. Furthermore, the role of arginine methylation as a crucial factor in the epigenetic regulation of gametocyte development has been proposed [266,278]. Various components of the ubiquitin machinery (E1, E2, E3) and deubiquitinases are reported for mosquito stages in genomic databases. The Skp1-Cullin1-FBXO1 protein complex, associated with ubiquitin machinery, was recently shown as a regulator required for the formation of P.b. gametes and motile forms [279]. The necessity of palmitoyl-S-acyl-transferase (DHHC1, 2 and 10) for P.b. ookinete development and malaria transmission has been demonstrated through both chemical and genetic approaches [280,281,282].

The utilization of the glutaminyl cyclase (QC)-mediated protein PTMs was demonstrated as the means by which the parasite develops its invasive strategy. This enzyme modifies N-terminal glutamine or glutamic acid residues of target proteins into cyclic pyroglutamic acid (pGlu) in both rodent and human malaria parasites. The evasion of the vector immune defense, involving this mechanism, was shown [283].

A computational approach suggested P.b. sporozoites in salivary glands influence proteolysis in the vectors [284]. In this study, RNA-sequences were used to compare the differential gene expression in the salivary glands of P.b.-infected and uninfected *Anopheles coluzzii* mosquitoes. The analysis revealed the changes in 2588 genes in the mosquitoes’ salivary glands in response to the P.b. infection, with 1578 genes showing upregulation and 1010 genes showing downregulation. The authors observed that genes associated with general metabolism, replication, immunity, transcription, proteolysis, translation, and molecular transport were among the mosquito genes most impacted by *Plasmodium*. Notably, within the differentially expressed genes in infected salivary glands, endopeptidase coding genes were the most abundant, which is of interest for post-translational modification studies [284]. In another study, in the sporozoite pre-salivary gland step, cysteine proteases were shown to be necessary for parasite-dependent proteolysis during sporozoite egress from oocysts, as P.b. egress-cysteine protease 1 (ECP1) and SERA-8 [17,29]. Another computational study analyzed multiple PTMs in published proteomics data from sexual stages, and numerous proteins from various *Plasmodium* species were described [263]. Six of the eight mitochondrial TCA enzymes were found to be modified, including aconitase modified by palmitoylation (essential during P.f. male gametocytogenesis and P.b. gametogenesis), and α-ketoglutarate dehydrogenase (KDH), essential for oocyst formation, was also modified by palmitoylation [263]. In P.f. gametocyte male organisms, some DNA binding proteins, as proliferating cell nuclear antigen 1 (PCNA1), were modified by multiple PTMs: acetylation, methylation, nitrosylation, glutathionylation, ubiquitination, and palmitoylation [263]. This protein was also shown to be important in schizont intra-erythrocyte development during nuclei replication, which deviates from a precise geometric 2n progression, with each proliferative cycle yielding a variable number of progeny [285]. Up to eight different modifications, including phosphorylation, acetylation, and glycosylation, were observed in BiP (also known as HSP70 or Grp78), enolase, and other proteins [263]. Acetylation and redox modifications, glutathionylation and nitrosylation, were found in chromatin assembly factor 1 (CAF-1) subunit C [286], implicated in depositing histones on replicated DNA [263]. Chromatin assembly-binding and DNA-binding proteins were found to undergo methylation at arginine residues, acetylation, ubiquitination, and nitrosylation. Minichromosome maintenance (MCM) DNA replication factors were found to be modified by acetylation (MCM3/4/6), arginine methylation (MCM4/5/6/7), ubiquitination (MCM2/7), and nitrosylation (MCM2/3/4/5/6/7), respectively [263]. Functionally, the MCM complex starts DNA replications, and in *Plasmodium*, it was shown (i) to be involved in early male gamete DNA replication, (ii) to be associated with, and (iii) to be phosphorylated by CDPK4 [287]. Acetylation PTMs were reported on replication factor C, DNA topoisomerase II, some subunits of DNA polymerase, DNA ligase I, and ORC subunit 1. The authors suggest that the atypical cell cycle driving male gamete formation may be regulated by the interplay between phosphorylation and acetylation [263].

The majority of the transcriptome in the female gametocyte is stored in RNA storage granules, maintained in a translationally repressed state. It is only when exposed to the mosquito environment that these transcripts are temporarily translated. It was shown that in P.b. gametocytes, RNA granules encompass various RNA-binding proteins (RBPs), such as Sm-like CITH, RNA helicase DOZI, Bruno homolog, Alba1-4, and Poly A binding proteins (PABP). Among these, DOZI and CITH are recognized for their roles in preserving mRNA stability [288]. In the above-described computational study [263], the authors discovered that PABP1 and Alba 4 contain at least six types of PTMs, including arginine and lysine methylation, acetylation, glutathionylation, palmitoylation, and nitrosylation. Additionally, Alba1-3, Musashi, DOZI, and PABP 2/3 exhibited from one to five different modifications [263]. Over the RBPs, the components of the translational machinery, as ribosomal subunits, initiation, and elongation factors (eIF and eEF), as eEF-2 and ribosomal stalk protein P0, were modified by a combination of acetylation, methylation, glycosylation, glutathionylation, ubiquitination, nitrosylation, and palmitoylation [263]. The authors encourage new studies on the roles of diverse PTMs in sexual stages for uncovering both the unique basic aspects of parasite biology and new ways for therapeutic interventions [263].

Summarizing, the PTMs described in sexual parasite forms and reported here indicate their importance for the parasite life in vector. Importantly, the interaction between the parasite and the mosquito immune system could be exploited for parasite elimination, e.g., by targeting protein PTMs for parasite control [283,289,290].

Modified proteins of parasites during mosquito stage, their PTMs, and the enzymes involved are summarized in Table 5.

## 3. Antimalarials and Protein Modifications

Due to the importance of all PTMs in parasite life, the related proteins and enzymes have been proposed as targets for antimalarials for some time [213]. Several candidates have been under study for decades, while some emerged recently and are under intense study now [9,13,213,293,294]. Here, we compiled some examples of protein PTM targeting by antimalarials.

### 3.1. Phosphorylation

Protein kinases (PKs) play key roles in the *Plasmodium* life cycle. Numerous review papers collected the data on *Plasmodium* kinases, proposed as drug targets [14,295]. Recently, bioinformatic analysis of eight *Plasmodium* species individuated 76 to 97 PKs across all *Plasmodium* spp. kinomes. They belong to the serine/threonine protein kinases from AGC group, calcium/calmodulin-dependent kinases CAMK, CMGC (named after CDK, MAPK, GSK3, and CLK families), casein kinase CK1, STE-, tyrosine kinase-like kinase TKL groups, and the *Plasmodium*-specific group FIKK [14]. All of them could be potential drug targets, but 37 protein kinases that cover the two most important species, P.f. and P.v., were favored [14]. A comparative analysis of the kinomes of P.f., P.v., and *Homo sapiens* was also conducted. This analysis described the similarities and differences between them, and discussed directions for kinase-directed drug discovery, emphasizing the importance of considering interspecies similarities within the *Plasmodium* genus. It was underlined that several *Plasmodium* kinases exhibit a high level of similarity with their human counterparts, which suggests they may not be well suited as targets for drug discovery [296]. The kinase similarity between *Plasmodium* spp. and other apicomplexan parasites, instead, is generally considered as a highly positive factor. *Plasmodium* kinases have major similarities to human kinases, and hence, poor drug targets are computationally individuated: 10 kinases from CMGC group, cyclin-dependent-like kinase 3 (CLK3), serine/arginine protein kinase 1 (SRPK1), casein kinase 2 alpha subunit (CK2a), MAPK1, and glycogen synthase kinase 3 (GSK3) [296]. Selected protein kinases (PKs) including Nek-1, CDPK1, CDPK4, PKG, and CLK3 were discussed as more promising in another review [231]. Specific gametocyte kinases for targeting were proposed [297]. A review on targeting PKGs for the development of new drugs was published [298]. The oral application of the inhibitor compound of PfPKG, ML10, cleared the P.f. parasitemia in the SCID mouse model and blocked the transmission of mature P.f. gametocytes to *Anopheles stephensi* mosquitoes [77]. Trisubstituted imidazole MMV030084 inhibited PfPKG, using tyrosine kinase-like protein 3 as a mediator, and affected *Plasmodium* sporozoite invasion into hepatocytes, merozoite egress, and male gamete exflagellation [299]. Takinib and its analog, HS220, small molecule inhibitors of PfPK9, were proposed as antimalarials. PfPK9 phosphorylates the *Plasmodium* E2 ubiquitin-conjugating enzyme PfUBC13, which mediates K63-linkage-specific polyubiquitination. Both proposed substances have been shown to be efficient against the liver stage [46]. Due to its importance in parasite metabolism throughout all stages of the parasite cycle, PfPKG has been proposed as a valid target, and PKG inhibitors have been studied for their antimalarial activity. Note, some inhibitors were active against the blood-cell stages of P.f. cultured in vitro, but showed no activity against the P.b. in mouse models [300]. P.f. kinase PfPK7 inhibitors imidazopyridazines and, recently, β-carbolines alkaloids were proposed as antimalarial substances [294,301,302,303].

Chalcones are plant-derived polyphenolic compounds from the flavonoids family. Chalcones 1,3-diaryl-2-propenones were proposed as CDK Pfmrk inhibitors, as they interrupted parasite cell cycle control and intra-erythrocyte differentiation [97,304]. PfGSK-3 kinase selective benzofuran-based or thieno [2,3-b] pyridine-based inhibitors were proposed for their selectivity and high anti-plasmodia activity [79,80]. Curcumin was shown to exert an antimalarial effect on P.b. involving the inhibition of mouse liver GSK3β and probably involving host immunomodulation [305]. Metformin, the activator of protein kinase AMPK and lipid metabolism modulator, was shown to be effective in liver protection, preventing *P. chabaudi* infection in mice [56]. Cyclin-dependent kinases generally were proposed as a drug target for numerous diseases, including malaria, and they are under continuous study [293,306]. PfCLK3, along with other important kinases such as phosphatidylinositol 4-kinase PI4KIIIβ or cGMP-dependent protein kinase, was referred to as a promising antimalarial target [306]. The involvement of CDKs in the dangerous artemisinin-induced parasite dormancy was shown. During the dormancy phase, parasites show dysregulation in numerous CDKs and related proteins. Starting the recovery phase, parasites quickly up-regulate cyclin and CDK genes (e.g., PfCRK1 and PfCRK4). Among others, efficient CDK inhibitors were proposed: roscovitine, WR636638, and olomoucine. They had distinct effects on various phases of DHA-induced dormancy, preventing parasite recovery [62]. Imidazopyridazines were reported to be inhibitors of the kinase PfCDPK1, and also to target cyclic GMP-dependent protein kinase and HSP90 to kill the parasite at different stages of P.f. intra-erythrocytic development. P.f. kinases such as PfCK2, PfPKG, and eIF2alpha kinase IK2 were reviewed as potential drug targets for the erythrocytic stages [259,307]. Host tyrosine-protein kinase Syk, which phosphorylates band 3 in infected erythrocytes, is shown to be important for parasite reinfection [308]. Syk inhibitors are shown to be effective antimalarials, interfering with the modifications on erythrocyte membranes elicited by parasite and subsequently suppressing parasite egress [309]. Additionally, Syk inhibitors synergized with artemisinin by enhancing oxidative stress in P.f.-parasitized erythrocytes [310]. Interestingly, M5717 as a specific inhibitor of *Plasmodium* eEF-2, an essential factor for protein synthesis in all parasite stages and the substrate of numerous PTMs, was shown to also be associated with kinases in the mechanism of action [311]. M5717 is undergoing clinical study as a multi-stage antimalarial [311,312,313]. PfAMA-1 and PvAMA-1, involved in serine protease action [30] and potentially glycosylated, can induce strong cellular and humoral responses, and are proposed and actively studied for vaccine development [31,111]. Transcriptomic analyses reveal that PfPP1 is essential for P.f. and is also a non-homologous protein to the human host, suggesting that it could be a viable drug target [107,108].

### 3.2. Proteolysis

The importance of cysteine, serine, aspartic, and metallo-proteases for *Plasmodium* spp. life cycle, particularly for hemoglobin digestion and host cell remodeling, was discussed above in the Section 2.3 and Section 2.5. Consequently, *Plasmodium* spp. proteases were considered optimal targets, and protease inhibitors (e.g., ICPs) were deemed to be promising antimalarials [314,315,316]. For example, cysteine proteases P.f. falcipains were reviewed as drug target [134]. Interestingly, the pro-oxidant action of dihydroartemisinin was recently connected to P.f. protease falcipain-1 damage, as this enzyme underwent lipoxidation modifications by 4-HNE [317]. Signal peptide peptidase inhibitors specific for PfSPP protease may function as potent antimalarial drugs both against blood-stage malaria [318] and liver forms [319,320]. Gametogenesis and transmission were successfully blocked by PMV inhibition [258]. Multistage antimalarials, which target the aspartic protease plasmepsins (mostly plasmepsin V, IX, and X) essential for all stages of parasite invasion, growth, and egress, have been proposed. This type of compounds is under continuing development [321,322]. Carbamoyl triazoles are serine protease inhibitors and are likely potent antimalarials [323]. Peptidomimetic nitrile inhibitors are falcipain-2 protease inhibitors: they showed high selectivity for human cathepsins, too, which matters for tumor treatment [324].

### 3.3. Acetylation, Methylation

Acetylation, methylation, and other epigenetic PTMs were reviewed as promising therapeutic targets [235]. In cases of parasite resistance to antimalarial blasticidin S and artemisinins, epigenetic PTMs are involved and could be targeted separately [235,325]. Blasticidin S resistance is connected with cytoadherence-linked asexual protein 3 (CLAG3), regulated by H3K9ac and H3K9me3 [326]. Recent studies have demonstrated that PfGCN5 plays a role in artemisinin resistance by enhancing the unfolded protein response (UPR) pathway and regulating 300–400 genes associated with stress responses [327,328]. The inhibition of P.f. histone acetyltransferases (HATs) has been extensively documented with embelin, curcumin, and anacardic acid. However, their use is fraught with nonspecific effects, including impacts relating to the disruption of chaperone expression, reactive oxygen species production, and lipoxygenase activities [235]. In contrast, CB3717 is a promising candidate, exhibiting robust selective inhibition of PfGCN5, which is significantly different from its human enzyme orthologue [119,329]. PfMYST presents another target due to its dissimilarity to its human counterpart. The thiazole derivative NU9056, known for inhibiting PfMYST catalytic activity, proves lethal for the parasite at a micromolar range [330]. Despite the limited number of described *Plasmodium* HAT inhibitors thus far, there is a need to assess new compounds designed to target HATs. These inhibitors could prove useful also for the therapy of other diseases, as HATs play important roles in various pathologies [331]. Among anti-plasmodia epidrugs, HDAC inhibitors (HDACi) are the most abundant, with different chemical structures, such as cyclic tetrapeptides, 2-aminosuberic acid derivatives, and L-cysteine derivatives. Originally designed to target human cancer cells, many of these inhibitors show significant activity against P.f., with IC50 values ranging from low nanomolar to sub-micromolar. However, most of them initially demonstrated low selectivity, although this is improving now [332,333,334,335]. Dihydroartemisinin–HDACi hybrid molecules were also proposed and evaluated [336]. Moreover, certain HKMT inhibitors have yielded promising results, exhibiting an acceptable selectivity index, and are undergoing in vivo testing [260,337].

### 3.4. Nitrosylation

Nitrosylation PTM is largely associated with stress conditions and host redox imbalance due to the production of reactive nitrogen and oxygen species (RNS/ROS), which could be damaged for parasite. It was proposed that this could be exploited for the adjuvant antimalarial therapy [338]. In mouse experimental cerebral malaria models, the effect of combined artesunate and tetramethylpyrazine treatment on host protein S-nitrosylation was studied [339]. This combination could significantly improve disease prognosis by ameliorating physiological parameters, reducing parasite, lymphocyte, and erythrocyte adhesions, increasing cerebral blood flow, and regulating endothelial, neuronal, and induced nitric oxide synthase (eNOS, nNOS, and iNOS, correspondently). The artesunate and tetramethylpyrazine were able to regulate the level of total S-nitrosothiols. An S-Nitroso-modified proteomic analysis was performed, and 917 S-nitrosylation proteins were identified in the treated and control groups. Among the differentially expressed proteins, 24 were downregulated and 21 were upregulated. Further detailed analysis of the S-nitrosylated proteins is required for therapeutic improvement [339].

### 3.5. Lipid Modifications

Lipid metabolism and lipid PTM-related enzymes and proteins were proposed as drug targets for malaria therapy [161,175,205,340]. Parasite enzymes for palmitoylation, myristoylation, and prenylation were proposed as efficient pharmaceutical targets [161,175,177,179]. An evaluation of N-myristoyltransferase as an antimalarial drug target was performed, and numerous potentially affected parasite proteins and their relative functions were defined [160,175,176,213,341]. Some examples are reported below. The mislocalization of rhoptries was shown after parasite treatment with 2-bromopalmitate (2-BP), the inhibitor of palmitoylation enzymes [163]. The promising imipramine-based N-myristoylation inhibitor, IMP-1002, was reported [342]. It blocked *P. falciparum* intraerythrocytic development, egress, and invasion [160].

The multidrug resistance protein PfMDR1 and the drug transporter PfCRT, which are located in the *P. falciparum* digestive vacuole membrane, have been shown to be modified by palmitoylation [168]. They have been proposed as potential drug targets to bypass antimalarial drug resistance [343,344].

Protein prenylation is generally very interesting for drug target research, as prenyltransferases (PTases) are generally involved in a large number of physiological and pathological processes, including malaria parasite metabolism [173,179,345,346]. For example, small molecule inhibitors of prenyl transferases were shown to elicit strong antimalarial activity by disrupting the Rab5 localization and food vacuolar integrity in numerous P.f. culturing strains [176]. Fosmidomycin, the inhibitor of 1-deoxy-D-xylulose 5-phosphate reductoisomerase (DXR), a key enzyme in the non-mevalonate pathway of isoprenoid biosynthesis for consequent protein isoprenylation, was used as an antimalarial drug in clinical studies [345]. Further research is ongoing to better understand its mechanism of action [346].

### 3.6. Alkylation, Glycosylation, Lipoxidation

Protein alkylation, more than just a target for antimalarials, is often the actual mechanism of action, or part of a more complex operation, for numerous antimalarials, including artemisinin, one of the primary antimalarial drugs [347,348,349,350]. Only a few examples of protein alkylation targeting are reported here. Derivatives of dextromethorphan targeted N-alkylation and showed antimalarial activity against the P.f. liver and blood stages, and stages I-II and V of gametocytes [351]. Glycosylation or deglycosylation of PfEMP1 and AMA1 was very important for human immune response during the development of VAR2CSA (important in placental malaria) and AMA1 vaccines, correspondently [241,242].

As was mentioned above, oxidation and lipoxidation PTMs are often damaging or deadly for parasite [183,205,212]. Among other natural endoperoxides, the natural product plakortin, an endoperoxide molecule extracted from the sponge *Plakortis*, has been shown to be an effective antimalarial agent. It induces high levels of reactive oxygen species and lipoperoxidation, leading to the formation of 4-HNE, which alkylates functional proteins identified by mass spectrometry: endoplasmatic reticulum-standing Hsp70-2 (BiP analogue), heat shock protein Hsp70-1, enolase, V-type proton ATPase catalytic subunit A, the dynein heavy chain-like protein, and the putative vacuolar protein sorting-associated protein 11 [352].

Artemisinin family antimalarials arouse numerous post-translational modifications during their antimalarial action. Although the exact mechanism of artemisinin action is multifaceted and still under discussion, the broad spectrum of its known effects has been largely described [348,353,354]. The mechanism, which has been considered for many years, can be summarized roughly: after activation by intra-parasitic heme iron, artemisinin exerts its effect by alkylating parasite proteins, ultimately leading to parasite death [347,348,354]. Artemisinin interferes with *Plasmodium* proteins involved in various metabolic pathways, including protein ubiquitination, unfolded protein response, eukaryotic translation initiation factor 2α activation, proteasome function, and phosphatidylinositol-3-kinase activity [348,353,354]. Additionally, artemisinin was reported to directly bind to sarcoendoplasmic reticulum calcium ATPase (SERCA), suppressing parasite growth [355]. Separately, artemisinin was shown to induce lipid peroxidation [212,317]. For example, P.f. protease falcipain 1 was modified by the lipoperoxidation end-product 4-HNE, which was generated by dihydroartemisinin at micromolar concentrations [317]. Similar 4-HNE modifications of human proteases cathepsins were proposed as a mechanism of artemisinin action on human tumor cells [317]. In summary, artemisinin action involves various protein PTMs, which could be mutually reinforced. Alkylation exercised by new endoperoxide drugs, inspired by artemisinin, will be exploited for their antimalarial action [356].

### 3.7. Ubiquitination and Others

Ubiquitination was proposed as a promising target for antimalarials as part of a protein degradation system [315]. As an example, ubiquitination was shown to be disturbed when mice were treated with liver-stage active antimalarial bulaquine, a derivative of 8-aminoquinoline [357]. Interestingly, the compound, 6-((7-nitrobenzo[c]1,2,5-oxadiazol-4-yl)thio)hexan-1-ol (NBDHEX), previously described for antitumor activity, was selectively active against the gametocyte P.f. stage. Covalent NBDHEX modifications, which could be considered as PTM, were shown by mass spectrometry in cysteines of gametocyte proteins: alpha tubulin 2 (Tub-α2), GAPDH, cell division cycle protein 48 (Cdc48), 14-3-3 isoform I (14-3-3I), and 60S ribosomal protein L7a (eL8). These modifications, which target different metabolic pathways, could be valuable mechanisms of NBDHEX action [358].

Note that protein PTMs could result in the formation of new epitopes able to elicit both adaptive and innate immune responses. This response can exhibit either protective or deleterious effects in the host organism. In this way, the host organism autonomously targets PTMs generated by parasites. Intervening in this process offers the potential to enhance host defense mechanisms more effectively.

Note also, HZ properties and their interactions within the parasite and the host (discussed in the Section 2.3) were proposed to exploit for antimalarial development [220]. Although the HZ formation in food vacuole by proteases is largely targeted by antimalarials, the depositions of HZ in host phagocytes and related processes must be further explored and possibly targeted.

## 4. Discussion

The wide spectrum of post-translational protein modifications identified in *Plasmodium* spp. along its life stages reflects the complexity of these organisms. In this review paper, we summarized the published data regarding protein PTMs in the parasite, highlight several important host and vector protein PTMs strictly connected with parasite development, and finally, outline therapeutic strategies aimed at targeting protein PTMs.

We reviewed here phosphorylation, methylation, acetylation, glycosylation, ubiquitination, glutathionylation, nitrosylation, lipidation (including lipoxidation), alkylation, biotinylation, and proteolysis.

The characterization of the proteins involved in PTM-related regulation was usually performed by gene analysis accompanied by proteomic and functional studies [359]. In the past, the importance of *Plasmodium* and host PTMs emerged from studies in which the proteins of the whole proteome were identified and analyzed [359,360]. Later proteomic studies were properly focused on protein PTMs [7,8]. A machine learning approach was applied to predict new phosphorylation sites in proteins of P.f. [361], whose results are the theoretic base for further experimental studies to confirm, or not, the prediction. Note that the majority of studies were performed with the most dangerous human and the rodent malaria parasites, P.f. and P.b., respectively. Recently, an increasing number of studies have reported data from the P.v. parasite due to its importance for healthcare, wide geographic distribution, and the recently improved methods for P.v. cultivation in the laboratory [21,111,149,196,265,296,362,363,364]. Further studies, however, are needed in order to deepen our understanding of protein PTMs in all *Plasmodium* species. Particularly, more studies in glycobiology are needed in order to explore protein glycosylation processes and exploit them for therapeutic purposes [365]. It is also striking that protein PTMs related to oxidative stress are rarely reported, even though oxidative stress, free-radical chain reactions, and lipid oxidation are crucial events in both parasite physiological development and induced conditions when the parasite became damaged [186,211,212]. The idea that strong oxidative events simply destroy the parasite integrity, diffusely targeting its membranes and structures, might be too crude, hence, subtler and site-directed oxidative modifications might play a role. More studies are needed to cover this field [205,212]. Apart from protein modifications, modified lipids deserve to be mentioned as a promising field for the future investigations of cellular regulation [366,367,368].

Since specific enzymes are directly involved in protein PTMs as modifiers and often as substrates, further studies are generally necessary in order to more thoroughly characterize all *Plasmodium* enzymes. This is crucial for advancing our basic knowledge and developing antimalarial therapies. The role of protein PTMs has already been established for numerous enzymes, while for others, this role must still be determined. Some promising enzymes in this regard include oxidative stress-related enzymes [369,370,371], cytochromes P450 and NADPH hemoprotein reductases [372], nucleotide biosynthesis-related [373,374], ion homeostasis-related, and energy metabolism-related enzymes [375,376,377,378,379,380].

It is important to note that many enzymes, substrates, and products involved in protein PTMs are highly specific to parasites, making them optimal therapeutic targets and ensuring host safety.

Protein PTMs can often create new epitopes or alter existing ones, leading to immune recognition and eliciting both innate and adaptive immune responses. Therefore, PTMs affect host–parasite interactions, which could be leveraged for vaccine development.

Personalized medicine is applied in various fields in order to tailor medical treatment to the individual characteristics of each patient. Although it is currently less utilized in malaria treatment, future prospects are encouraging [381], similar to the overall potential for precision medicine in malaria [382]. As with other diseases, where precision medicine also targets the active post-translational modification protein isoforms [383,384], in malaria, the focus could be on targeting protein PTMs involved mainly in parasite–host interactions.

Post-translational modifications (PTMs) are known to produce significant changes in intrinsically disordered proteins (IDPs) [385,386]. The properties of IDPs and their roles in different disorders are of high interest. IDPs are described in *Plasmodium* spp., and disordered protein regions have already been exploited for vaccines. Their further use could be suggested for antimalarial vaccine development [387,388].

Biological control tools for malaria eradication have been proposed [389,390]. Protein PTMs could play a significant role in these tools’ actions. For instance, when *Wolbachia* interferes with the *Plasmodium* developmental cycle in mosquitoes [391,392], the mechanism—though only partially understood—might also involve protein PTMs.

We additionally want to note that the *Plasmodium* PTMs and related proteins reported here are frequently found in other pathogenic organisms (e.g., *Toxoplasma gondii* or *Trypanosoma* spp.). The results of the studies discussed here for *Plasmodium* spp. can be extrapolated to the broader field of the pathophysiology and pharmacology of infectious diseases.

## 5. Conclusions

Owing to its intricate life cycle, the extensive array of post-translational protein modifications found in *Plasmodium* spp. is not unexpected. These modifications, along with the translational regulation of protein function, are abundantly present in all phases of parasite development. The synergism between different PTMs, which will amount to a copious number of combinations, must be further studied. Additionally, uncovering and understanding the regulation of all PTM-involved players, the so-called “regulators of regulators”, is crucial.

Further exploration is needed on the role of PTMs in host–parasite interactions, including the parasite’s evasion of vector and host protective responses. This includes how parasites manipulate the host’s physiological and defense processes. Among other topics, secretion processes, macrovesicle formation, and their regulation by PTMs are of high interest.

Generally, due to the involvement of protein PTMs in nearly all parasite metabolic processes and parasite–host interactions, understanding PTMs is crucial for the development of new antimalarial agents.

In summary, understanding protein PTM processes will contribute to our basic knowledge of parasite physiology, and offers promising avenues for the development of antimalarial strategies.

## Figures and Tables

**Table 1:** Modifying enzymes, modified proteins, and functions targeted by PTMs in *Plasmodium* sporozoites. NA—not mentioned or not studied by authors; *—hypothetical.

Enzyme	Modified Protein	PTM	Targeted Function	Refs.
PbCDPK1 ^§^, CDPK4, CDPK6	NA	Phosphorylation	Development and hepatocyte invasion	[37]
P.f., P.b. CDPK4 NEK-2, NEK-4 MAP-2 *	Microtubule-associated proteins	Phosphorylation	Microtubule-associated functions, motility *	[15,38]
PbCDPK1	MyoA	Phosphorylation	Gliding motility	[16,39]
PfCDPK-6	NA	Phosphorylation	Sporozoite formation	[17,37]
PfeIK1, PbIK2 (eIK2)	eIF2alpha	Phosphorylation	Stress response, translational shutdown	[25,26,27,28]
P.b. UIS2	eIF2alpha-P	Dephosphorylation	Activation of genes for invasion and in liver transformation	[26]
SERA-8	CSP	Proteolysis	Protein cleavage/processing	[17]
P.f. serine protease	PfAMA-1, TRAP	Proteolysis	Protein shedding. Hepatocyte invasion	[30]
Papain family cysteine protease	PbCSP	Proteolysis	Hepatocyte invasion	[40,41]
PbAtg4	PbAtg8	Proteolysis	Protein processing, liver merozoite development	[32,33,42]
PfSET7	P.f. H3K4, H3K9, H3K36	Methylation	NA	[19]
Transferase *	P.f., P.v. CSP, TRAP	Glycosylation	Hepatocyte invasion	[21,43]
NA	PfAMA-1	Glycosylation	Host immune recognition	[31]
PfPOFUT2	PfCSP	O-fucosylation	Protein stabilization and trafficking, folding quality control	[23,44]
PfPOFUT2	PfTRAP	O-fucosylation	Protein stabilization and trafficking, folding quality control *	[23]
PfUCHL3	NA	Deubiquitination	Development	[35]
PbDHHC3	P.b. inner membrane complex proteins	Palmitoylation	Gliding motility machinery	[36]

^§^ Abbreviations used in all tables. *Plasmodium falciparum* (Pf or P.f.); *Plasmodium berghei* (Pb or P.b.); *Plasmodium vivax* (Pv of P.v.); serine/threonine protein kinase (PK); myosin-A (MyoA); histone methylated on lysine: H(number of histone)K(number in lysine); histone-lysine N-methyltransferase (SET7); C-mannosyltransferase DPY19; thrombospondin-related adhesive protein (TRAP); O-GlcNAc transferase (OGT); myelin basic protein (MBP); mitochondria phosphate carrier protein (MCP); Asp-His-His-Cys family of palmitoyl acyltransferase (DHHC); calcium-dependent protein kinase (CDPK); calmodulin-dependent kinase (CaMK); O-fucosyltransferase (POFUT2); apical membrane antigen (AMA); eukaryotic initiation factor-2alpha (eIF2alpha) kinase (IK2 or eIK2); cysteine proteinase SERA; E2 ubiquitin-conjugating enzyme (UBC13); subtilisin-like protease (SUB); Site-2 protease form M50 family of metalloproteases (S2P); origin recognition complex subunit 1 (PfORC1); glycogen synthase kinase (GSK); cAMP-protein kinase A (PKA); calcium-dependent protein kinase B (PKB); 3-phosphoinositide-dependent protein kinase 1 (PDK1); nucleosome assembly protein with histone chaperone activities (NAPL); casein kinase (CK1); acetylation lowers binding affinity proteins (ALBAs); NIMA (Never In Mitosis A) mitotic kinase (NEK); Aurora family of kinases (ARK); microtubule-interacting protein EB1; splicing-related protein kinase (SRPK); serine/threonine protein phosphatase (PP); histone deacetylase (HDA or HDAC) SIR2; histone arginine methyl transferases (PRMTs); histone lysine methyl transferases (HKMTs); lysine-specific demethylase (LSD); Jumonji-C histone demethylase (JHDM); histone lysine demethylase form Jumonji-related family (PfJmjC); histo-aspartic protease (HAP); hemoglobin (Hb); aminopeptidase P (APP); signal peptide peptidase (SPP); glyceraldehyde-3-phosphate dehydrogenase (GAPDH); thioredoxin (Trx); thioredoxin reductase (TrxR); thioredoxin peroxidase (TPx); dihydrolipoamide dehydrogenase (LipDH); glutamate dehydrogenase (GDH); glyoxalase (Glo); ornithine δ-aminotransferase (OAT); lactate dehydrogenase (LDH); phosphoglycerate mutase (PGM); plasmoredoxin (Plrx); N-myristoyl transferase or glycylpeptide N-tetradecanoyltransferase (NMT); armadillo repeats-only protein (ARO); glutathione s-transferase (GST); glutathione reductase (GSR); chloroquine resistance transporter (CRT); farnesyl transferase (FTase, FT); geranylgeranyl transferase (GGT); autophagy-related proteins (Atgs); endoplasmic reticulum associated protein degradation enzyme system (ERAD); dipeptidyl aminopeptidase (DPAP); gametocyte-exported proteins (GEXPs); plasmepsin (PM); ribonucleotide reductase (RNR); subtilisin-like ookinete protein (SOPT); heterochromatin protein (HP); metallo-dependent protein phosphatase (PPM1); protein phosphatase with kelch-like domains (PPKL); α-ketoglutarate dehydrogenase (KDH); DNA binding protein proliferating cell nuclear antigen 1 (PCNA1); chromatin assembly factor 1 (CAF-1); minichromosome maintenance DNA replication factors (MCMs); RNA-binding protein (RBP); poly A binding proteins (PABP); cyclin-dependent kinase-like kinases (CLK); cyclin-dependent kinase (CDK); AAA+ ATPase from 19S proteasome (RPTi); rhomboid-family protease (ROM); phosphatidylinositol 3-kinase (PI3K); FYVE-containing coiled-coil protein (FCP); rhoptry protein 14 (ROP14); rhoptry neck protein 3 (RON3); trophozoite excretory protein (TEX1); inner membrane complex (IMC); rRNA 2′-O-methyltransferase fibrillarin (NOP1); DNA excision repair protein SNF2 helicase; vesicle transport regulators Ras-related proteins (RAB); adaptor protein complex protein (AP); spliceosomal Sm protein (SmD); acetyl-CoA carboxylase (ACC); circumsporozoite protein (CSP); 6-phosphofructokinase (PFK); acetyl-CoA synthetase (ACS); actin-I (ACT1); ring-exported protein-1 (REX1).

**Table 2:** Modifying enzymes, modified proteins, and functions targeted by PTMs in *Plasmodium* liver stage. NA—not mentioned or not studied by authors; *—hypothetical.

Enzyme	Modified Protein	PTM	Targeted Function	Refs.
PfPK9	PfUBC13	Phosphorylation	Parasite development	[46]
PbCDPK2	NA	Phosphorylation	Liver schizont development	[37]
PbMLFK	Proteins exported to the host	Phosphorylation	Protein export, host response	[47]
P.f. Src kinases Lyn, Lck, CrkL	PfTRAP	Phosphorylation	Cell signaling, hepatocyte invasion	[49]
PfCLK3, CDK	NA	Phosphorylation	Development	[61,62]
PfPKG	P.f. proteasome RPT1	Phosphorylation with consequent proteolysis	Proteasome processing	[45]
PbSUB1	PbMSP1, MSP6, MSP7	Proteolysis	Egress from hepatocytes	[50]
PbSERA-3	PbCSP *	Proteolysis	Egress from hepatocytes	[63]
P.b. S2P protease	P.b. transcription factors	Proteolysis	Parasite development	[54]
P.b. biotin ligase	P.b. ACC	Biotinylation	Apicoplast metabolism	[51]
PbDHHC3	P.b. inner membrane complex proteins	Palmitoylation	Liver invasion, egress	[36]

**Table 3:** Modifying enzymes, modified proteins, and functions targeted by PTMs in *Plasmodium* blood asexual stage. NA—not mentioned or not studied by authors; *—hypothetical.

Enzyme	Modified Protein	PTM	Targeted Function	Refs.
PfPK4	PfeIF2α	Phosphorylation	Environmental stress response	[74]
PfPK5	PfORC1	Phosphorylation	DNA replication, var gene regulation	[73]
Pfmrk and PfPK5	NA	Phosphorylation	Cell cycle control, differentiation	[97]
PfPK6	NA	Phosphorylation	Cell cycle control, differentiation of trophozoites/schizonts	[93]
PfPK7	1047 different P.f. proteins	Phosphorylation	Parasite development, ubiquitin/proteasome system	[70,71,72]
PfNEK-1, Aurora	NA	Phosphorylation	Development	[98,231]
PfCLK3	NA	Phosphorylation	Development	[61]
P.f., P.b. PKG	NA	Phosphorylation	Late-stage schizont development	[10,76]
P.f. cAMP-PKA, GSK-3	PfAMA1, glycogen synthase	Phosphorylation	Parasite motility, egress, and invasion	[11,67,111,232]
PfPKA, PKB	P.f. myosin A, GAP45, CDPK1	Phosphorylation	Glideosome function	[11,81]
PfCDPK1	PfGAP45	Phosphorylation	Schizont development	[82]
PfCDPK1	P.f. RhopH3	Phosphorylation	Invasion	[104]
PfCDPK1, CDPK5, CDPK7	PfMBP, MCP, NPT1, histone H2B *	Phosphorylation	Merozoite attachment, invasion	[27,233]
PfPDK1	PfPKA, PI3K	Phosphorylation	Invasion	[9,83]
Host casein kinase II *	PfNAPL, EMP1	Phosphorylation	Nucleosome assembly	[84,91]
PfCK1, PfCK2	PfNAPS, histones, ALBAs	Phosphorylation	Chromatin dynamics	[86,87,88]
PfMap-2	P.f. transcriptional regulators *	Phosphorylation	Schizogony	[99]
PfFIKK3, FIKK9.1, FIKK9.5, FIKK10.1, FIKK10.2	Host erythrocyte spectrin, ankyrin, band-3	Phosphorylation	Trafficking of parasite proteins to the host	[14,102,103]
PfSRPK1	Splicing factor PfSR1	Phosphorylation	mRNA splicing	[105]
PfCDPK *	PfCRT	Phosphorylation	Drug resistance	[168]
P.f. CaMK	NA	Phosphorylation	Development	[37]
NA	PfAtg proteins	Phosphorylation, lipidation, ubiquitination	Apicoplast maintenance, membrane trafficking of proteins	[187]
PfGCN5, PfMYST	P.f. H3K9ac, H3K4me2, H3K4me3, H3K9me3	Acetylation, methylation	Var gene regulation	[8,118,119,120]
NA	P.f. 14-3-3, 20S proteasome beta subunit, PFK, ACS, ACT1, elongation factors 1 and 2, enolase, ApiAP2, ADA2	Acetylation	Development	[9,112,234]
PfSIR2 (PfSIR2A, PfSIR2B), sirtuins, PfHDAC1, PfHDA2	P.f. H3K9ac, H3K14ac, H4K16ac	Deacetylation	Silencing of genes, antigenic variation, rDNA transcription	[8,115,116,235]
PfPRMT1, PfPRMT5	P.f. H4R3me, PfSmD1, other non-histone proteins	Methylation	Development	[122,127]
HKMTs: PfSET2, PfSET7, PfSET10	P.f. H3K4me, H3K36me	Methylation	Var gene regulation	[19,123,124,125]
NA	PfROP14, RON3, TEX1, 6-cysteine protein (p12), rifin, PfEMP1, IMC 1g, 1c	Methylation	Invasion	[126]
NA	PfNOP1, ALBA1, DEAD/DEAH helicase, 40S ribosomal proteins, eIF2, NAPL, MCM4-7, SNF2 helicase, RAD50, RAB1-B, RAB11-A, RAB18, AP-1	Methylation	RNA translation, chromatin organization, DNA replication, DNA repair, protein trafficking	[121]
PfLSD1, JHDM, PfJmjC1,2,3	P.f. histones	Demethylation	Development	[128,129,235]
PfDPY19	PfTRAP	C-mannosylation	NA	[236]
PfOGT	P.f. Hsp70 and α-tubulin	O-GlcNAc-glycosilation	Survival	[237]
P.f. falcipain-2, -3, falcilysin PfAPP	Host Hb, P.f. proteins	Proteolysis	Intraerythrocytic development, Hb digestion	[130,131,134,144,145]
PfPMs, HAP	Host Hb, PfEMP1	Proteolysis	Protein degradation, export and exposure, merozoite egress	[130,141,142,143]
PfSPP, M18 aspartyl aminopeptidase	P.f. signal peptides	Proteolysis	Parasite invasion and growth	[146,147]
P.f. falcipain-1, DPAP3	P.f. proteins	Proteolysis	invasion	[131,135]
PfSERA-4, -5, -6	Parasite and host proteins	Proteolysis	Egress form erythrocyte	[7,63]
PfSUB2 *	PfAMA-1, MSP1, MSP7, TRAMP/PTTRAMP	Proteolysis	Invasion	[238,239]
PfPP1	NA	Proteolysis	Glucose metabolism, DNA synthesis for segmentation	[107,108,240]
P.f. calcineurin	PfHSP90 *	Dephosphorylation, proteolysis	Late-stage schizonts growth. Invasion	[109,110]
Pf-calpain, subtilisin-1, -2, -3,	NA	Proteolysis	Calcium modulation, trophozoite development, invasion, egress	[7,136,137]
P.f. ROMs	PfTRAP, CTRP, MTRAP, PFF0800c, EBA-175, BAEBL, JESEBL, MAEBL, AMA1, Rh1, Rh2a, Rh2b, Rh4	Proteolysis	Invasion, egress	[138,139,140]
P.f. S-nitrosylase *	319 potential P.f. targets, GAPDH, PfTrx1	S-nitrosylation	Carbohydrate metabolism, parasite growth	[153]
NA	PfEMP1, AMA1, MSP1, MSP2	N-linked glycosylation	Development, invasion	[155,158,241,242]
NA	P.f. TrxR, Trx, Tpx1, GSR, GST, Plrx, mitochondrial LipDH, GDH1, Glo1/2, OAT, LDH, GAPDH, PK, PGM	S-glutathionylation	Protein functional inhibition	[159]
P.f. glutaredoxin 1, thioredoxin 1, plasmoredoxin	Same as above	Deglutathionylation	Protein activation	[159]
PfNMT	PfGAP45, ARO, others	Myristoylation	Development, egress, invasion	[8,160,164,175]
NA	PfGAP45, PfCRT, PfMDP1, ARO, MTIP, Alveolin, others	Palmitoylation	Development, egress, invasion	[162,163,164,168]
P. yoelii and P.f. DHHC2, DHHC7	CDPK1, GAP45	Palmitoylation	Rhoptry-related parasite invasion	[169,170,171]
PfFT, GGT	PfRabs, PfYkt6p, FCP, HSP40, others	Prenylation	Food vacuole functionality, others	[9,172,176,177,178,179,243]
P.f. ERAD enzymes: E1, E2, E3	NA	Ubiquitination	Cell-cycle machinery	[34,188,189]
P.f., P.b. Ubc13	NA	Ubiquitination	Development	[194,195]
NA	major subunit of RNA polymerase II, ApiAP2	Ubiquitination	Development	[192]
NA	PfREX1	Ubiquitination	Nutrient import in trophozoites	[191]
PfUCHL3	NA	Deubiquitination	Parasite development	[35,193]

**Table 4:** Modifying enzymes, modified proteins, and functions targeted by PTMs in *Plasmodium* gametocytes in the host. Some information relates to both gametocytes in the host (before mosquito ingestion) and in the mosquito after ingestion, when authors report it together. NA—not mentioned or not studied by authors; *—hypothetical.

Enzyme	Modified Protein	PTM	Targeted Function	Refs.
P.f. and P.b. CDPK4, NEK-2, NEK-4, MAP-2 *	P.f. and P.b. microtubule-associated proteins	Phosphorylation	Male exflagellation, motility *	[15,38,100,101]
PfPKG	NA	Phosphorylation	Rounding shape, exflagellation	[77,264]
PfPKA	NA	Phosphorylation	Deformability	[83]
NA	Pfg27	Phosphorylation	RNA oligomerization	[253]
PfCK2α	P.f. transcription factors, nuclear proteins *	Phosphorylation	Development	[88]
P.b. calcineurin	NA	Dephosphorylation, proteolysis	Male gametogenesis, fertilization	[109]
PfSUB2, PfDPAP2	Pfg377 *	Proteolysis	Osmiophilic body function	[255]
PfGSK-3	P.f. glycogen synthase	Proteolysis	Maturation	[232]
PfSUB2	NA	Proteolysis	Egress from erythrocyte	[255]
P.f. plasmepsins PMX, PMV	NA	Proteolysis	Gametogenesis	[257,258]
NA	PfGEXPs	N-acetylation	Early-stages development	[256]
P.f. acetyltransferases, methyltransferases	P.f. H2A, H2A.Z, H2B, H2B.Z, H3, H3.3, H4	Acetylation, methylation	Development	[246]
PfHDA2, PfSIR2	NA	Deacetylation	Conversion by PfAP2-G	[115,116,261]
NA	P.f. H3BK112	Ubiquitination	Development	[246]
PfDHHC9	NA	Palmitoylation	Development	[262]
NA	P.f. protein phosphatase PPM2	Acetylation, methylation, glutathionylation, nitrosylation palmitoylation, ubiquitination	Regulation of sex allocation	[12]

**Table 5:** Modifying enzymes, modified proteins, and functions targeted by PTMs in *Plasmodium* mosquito stages. NA—not mentioned or not studied by authors; *—hypothetical.

Enzyme	Modified Protein	PTM	Targeted Function	Refs.
PbCDPK1, CDPK2, CRK5	NA	Phosphorylation	Development of the male gametes	[240,252,291]
PbARK2	P.b. EB1, MISFIT, Myosin-K	Phosphorylation	Gametocyte spindle dynamics in chromosome segregation	[275]
P.f. Aurora B *	P.f. H3S10ph	Phosphorylation	PfHP1-related gametocyte differentiation	[266]
NA	P.b. HSP70, WD40 repeat protein msi1, enolase, actin-1, RNR, others	Phosphorylation	Gametocyte cytoskeleton, HSPs, DNA synthesis, signaling	[263,269]
PbSRPK	NA	Phosphorylation	Microgamete formation	[38]
PfCDPK1, CDPK2, CDPK3, CDPK4, CDPK6	NA	Phosphorylation	Ookinete infectivity and development	[37]
PbPPM1	NA	Dephosphorylation	Exflagellation	[12]
P.b. calcineurin	NA	Dephosphorylation, proteolysis	Gamete development, fertilization, ookinete-to-oocyst	[109]
PbSERA-3	NA	Proteolysis	Male gametocyte egress	[63]
NA	P.f. H3K9ac, H3K14ac H3K18ac, H3K27ac, H3K56ac, H4K8ac, H4K16ac, H4ac4	Acetylation	Gametocytes gene activation	[266,277]
NA	P.f. H2A.Z, H3K4me3, H3K9me3, H3K27me2, H3K36me3, H3K37me, H3R17mec, H4K20me	Methylation	Gametocyte differentiation	[266,277,278]
PfDRY19	PfTRAP	C-mannosylation	Gametocyte egress and exflagellation	[292]
P.b. Skp1-Cullin1-FBXO1 complex in ubiquitin machinery	NA	Ubiquitination	Gametocyte development	[279]
NA	PfPCNA1	Acetylation, methylation, glutathionylation, nitrosylation palmitoylation, ubiquitination	Male gametocyte development	[263]
NA	PfCAF-1 subunit C	Glutathionylation, nitrosylation, acetylation	Male gametocyte depositing histones on replicated DNA	[263,286]
CDPK4, others	P.f. MCM2-7	Phosphorylation, methylation, acetylation, nitrosylation, ubiquitination	Male gametocyte development	[263]
NA	P.f. DNA polymerase, replication factor C, DNA ligase I, DNA topoisomerase II, ORC subunit 1	Phosphorylation, acetylation	Male gametocyte development	[263]
NA	P.b. RBP: Alba 4, PABP1	Acetylation, methylation, glutathionylation, nitrosylation, palmitoylation	Female gametocyte development	[263]
NA	P.b. RBP: DOZI, Musashi, Alba1-3, PABP 2/3, CITH; translational proteins	Numerous PTMs	Female gametocyte development	[263]
NA	P.f., P.b. aconitase, KDH	Palmitoylation	Gametocytes, oocysts mitochondrial TCA cycle	[263]
PbPPKL	NA	Dephosphorylation	Ookinete differentiation, motility	[276]
PfSUB, SOPT serine proteases *	NA	Proteolysis	Ookinete development	[273]
P.b. DHHC1, 2, 10	NA	Palmitoylation	Ookinete development	[280,281,282]
PbMLFK, CK	NA	Phosphorylation	Sporozoite growth in oocyst	[47,272]
P. b. ATP-dependent protease	NA	Proteolysis	Sporozoite growth in oocyst	[272]
PbECP1, SERA-5	PbCSP processing *	Proteolysis	Egress from oocyst	[29,63]
ROMs	P. f. adhesins	Proteolysis	Invasion, egresses	[140]
CDPK4 NEK-2, NEK-4 MAP-2 *	P.f., P.b. microtubule-associated proteins	Phosphorylation	Motility (sporozoites, ookinetes, oocysts) *	[15,38]
P.b., P.v. CDPK1	P.b., P.v. MyoA, P.v. GAP40, GAP45, GAPM2, IMC proteins,	Phosphorylation	Motility (sporozoites, ookinetes)	[16,21,39]
NA	P.f., P.v. CSP, TRAP	Glycosylation	Invasion of salivary gland	[21,43]
DHHC3	P.b. proteins form the inner membrane complex	Palmitoylation	Sporozoite gliding motility	[36]

## Data Availability

The data presented in this study are available on request from the corresponding author.

## References

[1] World Malaria Report 2023, Updated 30 November 2023. https://www.who.int/teams/global-malaria-programme/reports/world-malaria-report-2023.

[2] Poespoprodjo J.R., Douglas N.M., Ansong D., Kho S., Anstey N.M. (2023). Malaria. Lancet.

[3] Malaria Report by CDC (The Centers for Disease Control and Prevention). Updated in 2024. https://www.cdc.gov/dpdx/malaria/index.html.

[4] Sato S. (2021). Plasmodium—A brief introduction to the parasites causing human malaria and their basic biology. J. Physiol. Anthropol..

[5] Pattaradilokrat S., Wu J., Xu F., Su X.Z. (2022). The origins, isolation, and biological characterization of rodent malaria parasites. Parasitol. Int..

[6] Srivastava A., Philip N., Hughes K.R., Georgiou K., MacRae J.I., Barrett M.P., Creek D.J., McConville M.J., Waters A.P. (2016). Stage-Specific Changes in Plasmodium Metabolism Required for Differentiation and Adaptation to Different Host and Vector Environments. PLoS Pathog..

[7] Chung D.W., Ponts N., Cervantes S., Le Roch K.G. (2009). Post-translational modifications in Plasmodium: More than you think!. Mol. Biochem. Parasitol..

[8] Doerig C., Rayner J.C., Scherf A., Tobin A.B. (2015). Post-translational protein modifications in malaria parasites. Nat. Rev. Microbiol..

[9] Rashidi S., Tuteja R., Mansouri R., Ali-Hassanzadeh M., Shafiei R., Ghani E., Karimazar M., Nguewa P., Manzano-Román R. (2021). The main post-translational modifications and related regulatory pathways in the malaria parasite *Plasmodium falciparum*: An update. J. Proteom..

[10] Taylor H.M., McRobert L., Grainger M., Sicard A., Dluzewski A.R., Hopp C.S., Holder A.A., Baker D.A. (2010). The malaria parasite cyclic GMP-dependent protein kinase plays a central role in blood-stage schizogony. Eukaryot. Cell.

[11] Lasonder E., Green J.L., Camarda G., Talabani H., Holder A.A., Langsley G., Alano P. (2012). The *Plasmodium falciparum* schizont phosphoproteome reveals extensive phosphatidylinositol and cAMP-protein kinase A signaling. J. Proteome Res..

[12] Guttery D.S., Poulin B., Ramaprasad A., Wall R.J., Ferguson D.J., Brady D., Patzewitz E.M., Whipple S., Straschil U., Wright M.H. (2014). Genome-wide functional analysis of Plasmodium protein phosphatases reveals key regulators of parasite development and differentiation. Cell Host Microbe.

[13] Ong H.W., Adderley J., Tobin A.B., Drewry D.H., Doerig C. (2023). Parasite and host kinases as targets for antimalarials. Expert Opin. Ther. Targets.

[14] Borba J.V.V.B., Silva A.C.E., do Nascimento M.N., Ferreira L.T., Rimoldi A., Starling L., Ramos P.I.P., Costa F.T.M., Andrade C.H. (2022). Update and elucidation of Plasmodium kinomes: Prioritization of kinases as potential drug targets for malaria. Comput. Struct. Biotechnol. J..

[15] Doerig C., Tobin A.B. (2010). Parasite protein kinases: At home and abroad. Cell Host Microbe.

[16] Ripp J., Smyrnakou X., Neuhoff M.T., Hentzschel F., Frischknecht F. (2022). Phosphorylation of myosin A regulates gliding motility and is essential for Plasmodium transmission. EMBO Rep..

[17] Le Roch K.G., Zhou Y., Blair P.L., Grainger M., Moch J.K., Haynes J.D., De La Vega P., Holder A.A., Batalov S., Carucci D.J. (2003). Discovery of gene function by expression profiling of the malaria parasite life cycle. Science.

[18] Coppi A., Tewari R., Bishop J.R., Bennett B.L., Lawrence R., Esko J.D., Billker O., Sinnis P. (2007). Heparan sulfate proteoglycans provide a signal to Plasmodium sporozoites to stop migrating and productively invade host cells. Cell Host Microbe.

[19] Chen P.B., Ding S., Zanghì G., Soulard V., DiMaggio P.A., Fuchter M.J., Mecheri S., Mazier D., Scherf A., Malmquist N.A. (2016). *Plasmodium falciparum* PfSET7: Enzymatic characterization and cellular localization of a novel protein methyltransferase in sporozoite, liver and erythrocytic stage parasites. Sci. Rep..

[20] Doud M.B., Koksal A.C., Mi L.Z., Song G., Lu C., Springer T.A. (2012). Unexpected fold in the circumsporozoite protein target of malaria vaccines. Proc. Natl. Acad. Sci. USA.

[21] Swearingen K.E., Lindner S.E., Flannery E.L., Vaughan A.M., Morrison R.D., Patrapuvich R., Koepfli C., Muller I., Jex A., Moritz R.L. (2017). Proteogenomic analysis of the total and surface-exposed proteomes of *Plasmodium vivax* salivary gland sporozoites. PLoS Negl. Trop. Dis..

[22] Swearingen K.E., Lindner S.E., Shi L., Shears M.J., Harupa A., Hopp C.S., Vaughan A.M., Springer T.A., Moritz R.L., Kappe S.H. (2016). Interrogating the Plasmodium Sporozoite Surface: Identification of Surface-Exposed Proteins and Demonstration of Glycosylation on CSP and TRAP by Mass Spectrometry-Based Proteomics. PLoS Pathog..

[23] Lopaticki S., Yang A.S.P., John A., Scott N.E., Lingford J.P., O’Neill M.T., Erickson S.M., McKenzie N.C., Jennison C., Whitehead L.W. (2017). Protein O-fucosylation in *Plasmodium falciparum* ensures efficient infection of mosquito and vertebrate hosts. Nat. Commun..

[24] Sanz S., Aquilini E., Tweedell R.E., Verma G., Hamerly T., Hritzo B., Tripathi A., Machado M., Churcher T.S., Rodrigues J.A. (2019). Protein O-Fucosyltransferase 2 Is Not Essential for *Plasmodium berghei* Development. Front. Cell. Infect. Microbiol..

[25] Zhang M., Fennell C., Ranford-Cartwright L., Sakthivel R., Gueirard P., Meister S., Caspi A., Doerig C., Nussenzweig R.S., Tuteja R. (2010). The Plasmodium eukaryotic initiation factor-2alpha kinase IK2 controls the latency of sporozoites in the mosquito salivary glands. J. Exp. Med..

[26] Zhang M., Mishra S., Sakthivel R., Fontoura B.M., Nussenzweig V. (2016). UIS2: A Unique Phosphatase Required for the Development of Plasmodium Liver Stages. PLoS Pathog..

[27] Turque O., Tsao T., Li T., Zhang M. (2016). Translational Repression in Malaria Sporozoites. Microb. Cell.

[28] Fennell C., Babbitt S., Russo I., Wilkes J., Ranford-Cartwright L., Goldberg D.E., Doerig C. (2009). PfeIK1, a eukaryotic initiation factor 2alpha kinase of the human malaria parasite *Plasmodium falciparum*, regulates stress-response to amino-acid starvation. Malar. J..

[29] Aly A.S., Matuschewski K. (2005). A malarial cysteine protease is necessary for Plasmodium sporozoite egress from oocysts. J. Exp. Med..

[30] Silvie O., Franetich J.F., Rénia L., Mazier D. (2004). Malaria sporozoite: Migrating for a living. Trends Mol. Med..

[31] Uddin N., Hoessli D.C., Butt A., Kaleem A., Iqbal Z., Afzal I., Muhammad H., Zamani Z., Shakoori A.R. (2012). O-GlcNAc modification of the anti-malarial vaccine candidate PfAMA1: In silico-defined structural changes and potential to generate a better vaccine. Mol. Biol. Rep..

[32] Maruyama T., Noda N. (2018). Autophagy-regulating protease Atg4: Structure, function, regulation and inhibition. J. Antibiot..

[33] Mishra A., Varshney A., Mishra S. (2023). Regulation of Atg8 membrane deconjugation by cysteine proteases in the malaria parasite *Plasmodium berghei*. Cell. Mol. Life Sci..

[34] Dalhuisen T., Plenderleith L.J., Ursani I., Philip N., Hahn B.H., Sharp P.M. (2023). Unusually Divergent Ubiquitin Genes and Proteins in Plasmodium Species. Genome Biol. Evol..

[35] Imhoff R.D., Rosenthal M.R., Ashraf K., Bhanot P., Ng C.L., Flaherty D.P. (2023). Identification of covalent fragment inhibitors for *Plasmodium falciparum* UCHL3 with anti-malarial efficacy. Bioorg. Med. Chem. Lett..

[36] Hopp C.S., Balaban A.E., Bushell E.S., Billker O., Rayner J.C., Sinnis P. (2016). Palmitoyl transferases have critical roles in the development of mosquito and liver stages of Plasmodium. Cell. Microbiol..

[37] Ghartey-Kwansah G., Yin Q., Li Z., Gumpper K., Sun Y., Yang R., Wang D., Jones O., Zhou X., Wang L. (2020). Calcium-dependent Protein Kinases in Malaria Parasite Development and Infection. Cell Transplant..

[38] Tewari R., Straschil U., Bateman A., Böhme U., Cherevach I., Gong P., Pain A., Billker O. (2010). The systematic functional analysis of Plasmodium protein kinases identifies essential regulators of mosquito transmission. Cell Host Microbe.

[39] Sebastian S., Brochet M., Collins M.O., Schwach F., Jones M.L., Goulding D., Rayner J.C., Choudhary J.S., Billker O. (2012). A Plasmodium calcium-dependent protein kinase controls zygote development and transmission by translationally activating repressed mRNAs. Cell Host Microbe.

[40] Coppi A., Pinzon-Ortiz C., Hutter C., Sinnis P. (2005). The Plasmodium circumsporozoite protein is proteolytically processed during cell invasion. J. Exp. Med..

[41] Geens R., Stanisich J., Beyens O., D’Hondt S., Thiberge J.M., Ryckebosch A., De Groot A., Magez S., Vertommen D., Amino R. (2024). Biophysical characterization of the *Plasmodium falciparum* circumsporozoite protein’s N-terminal domain. Protein Sci..

[42] Voss C., Ehrenman K., Mlambo G., Mishra S., Kumar K.A., Sacci J.B., Sinnis P., Coppens I. (2016). Overexpression of *Plasmodium berghei* ATG8 by Liver Forms Leads to Cumulative Defects in Organelle Dynamics and to Generation of Noninfectious Merozoites. mBio.

[43] Goerdeler F., Seeberger P.H., Moscovitz O. (2021). Unveiling the Sugary Secrets of Plasmodium Parasites. Front. Microbiol..

[44] Srivastava P.N., Paul P., Mishra S. (2024). Protein O-Fucosyltransferase Is Required for the Efficient Invasion of Hepatocytes by *Plasmodium berghei* Sporozoites. ACS Infect. Dis..

[45] Govindasamy K., Khan R., Snyder M., Lou H.J., Du P., Kudyba H.M., Muralidharan V., Turk B.E., Bhanot P. (2018). *Plasmodium falciparum* Cyclic GMP-Dependent Protein Kinase Interacts with a Subunit of the Parasite Proteasome. Infect. Immunol..

[46] Raphemot R., Eubanks A.L., Toro-Moreno M., Geiger R.A., Hughes P.F., Lu K.Y., Haystead T.A.J., Derbyshire E.R. (2019). Plasmodium PK9 Inhibitors Promote Growth of Liver-Stage Parasites. Cell. Chem. Biol..

[47] Jaijyan D.K., Verma P.K., Singh A.P. (2016). A novel FIKK kinase regulates the development of mosquito and liver stages of the malaria. Sci. Rep..

[48] Kaushansky A., Douglass A.N., Arang N., Vigdorovich V., Dambrauskas N., Kain H.S., Austin L.S., Sather D.N., Kappe S.H. (2015). Malaria parasites target the hepatocyte receptor EphA2 for successful host infection. Science.

[49] Akhouri R.R., Sharma A., Malhotra P., Sharma A. (2008). Role of *Plasmodium falciparum* thrombospondin-related anonymous protein in host-cell interactions. Malar. J..

[50] Tawk L., Lacroix C., Gueirard P., Kent R., Gorgette O., Thiberge S., Mercereau-Puijalon O., Ménard R., Barale J.C. (2013). A key role for Plasmodium subtilisin-like SUB1 protease in egress of malaria parasites from host hepatocytes. J. Biol. Chem..

[51] Dellibovi-Ragheb T.A., Jhun H., Goodman C.D., Walters M.S., Ragheb D.R.T., Matthews K.A., Rajaram K., Mishra S., McFadden G.I., Sinnis P. (2018). Host biotin is required for liver stage development in malaria parasites. Proc. Natl. Acad. Sci. USA.

[52] Mikolajczak S.A., Sacci J.B., De La Vega P., Camargo N., VanBuskirk K., Krzych U., Cao J., Jacobs-Lorena M., Cowman A.F., Kappe S.H. (2011). Disruption of the *Plasmodium falciparum* liver-stage antigen-1 locus causes a differentiation defect in late liver-stage parasites. Cell. Microbiol..

[53] Nicoll W.S., Sacci J.B., Rodolfo C., Di Giacomo G., Piacentini M., Holland Z.J., Doerig C., Hollingdale M.R., Lanar D.E. (2011). *Plasmodium falciparum* liver stage antigen-1 is cross-linked by tissue transglutaminase. Malar. J..

[54] Koussis K., Goulielmaki E., Chalari A., Withers-Martinez C., Siden-Kiamos I., Matuschewski K., Loukeris T.G. (2017). Targeted Deletion of a Plasmodium Site-2 Protease Impairs Life Cycle Progression in the Mammalian Host. PLoS ONE.

[55] Sharma P.K., Kalia I., Kaushik V., Brünnert D., Quadiri A., Kashif M., Chahar K.R., Agrawal A., Singh A.P., Goyal P. (2021). STK35L1 regulates host cell cycle-related genes and is essential for Plasmodium infection during the liver stage of malaria. Exp. Cell Res..

[56] Kluck G.E.G., Wendt C.H.C., Imperio G.E.D., Araujo M.F.C., Atella T.C., da Rocha I., Miranda K.R., Atella G.C. (2019). Plasmodium Infection Induces Dyslipidemia and a Hepatic Lipogenic State in the Host through the Inhibition of the AMPK-ACC Pathway. Sci. Rep..

[57] Prado M., Eickel N., De Niz M., Heitmann A., Agop-Nersesian C., Wacker R., Schmuckli-Maurer J., Caldelari R., Janse C.J., Khan S.M. (2015). Long-term live imaging reveals cytosolic immune responses of host hepatocytes against Plasmodium infection and parasite escape mechanisms. Autophagy.

[58] Zheng H., Lu X., Li K., Zhu F., Zhao C., Liu T., Ding Y., Fu Y., Zhang K., Zhou T. (2022). ATG Ubiquitination Is Required for Circumsporozoite Protein to Subvert Host Innate Immunity Against Rodent Malaria Liver Stage. Front. Immunol..

[59] Agop-Nersesian C., De Niz M., Niklaus L., Prado M., Eickel N., Heussler V.T. (2017). Shedding of host autophagic proteins from the parasitophorous vacuolar membrane of *Plasmodium berghei*. Sci. Rep..

[60] Maruthi M., Singh D., Reddy S.R., Mastan B.S., Mishra S., Kumar K.A. (2017). Modulation of host cell SUMOylation facilitates efficient development of *Plasmodium berghei* and Toxoplasma gondii. Cell. Microbiol..

[61] Martín Moyano P., Němec V., Paruch K. (2020). Cdc-Like Kinases (CLKs): Biology, Chemical Probes, and Therapeutic Potential. Int. J. Mol. Sci..

[62] Gray K.A., Gresty K.J., Chen N., Zhang V., Gutteridge C.E., Peatey C.L., Chavchich M., Waters N.C., Cheng Q. (2016). Correlation between Cyclin Dependent Kinases and Artemisinin-Induced Dormancy in *Plasmodium falciparum* In Vitro. PLoS ONE.

[63] Arisue N., Palacpac N.M.Q., Tougan T., Horii T. (2020). Characteristic features of the SERA multigene family in the malaria parasite. Parasit. Vectors.

[64] Wang J., Jiang N., Sang X., Yang N., Feng Y., Chen R., Wang X., Chen Q. (2021). Protein Modification Characteristics of the Malaria Parasite *Plasmodium falciparum* and the Infected Erythrocytes. Mol. Cell. Proteom..

[65] Painter H.J., Chung N.C., Sebastian A., Albert I., Storey J.D., Llinás M. (2018). Genome-wide real-time in vivo transcriptional dynamics during *Plasmodium falciparum* blood-stage development. Nat. Commun..

[66] Wilkes J.M., Doerig C. (2008). The protein-phosphatome of the human malaria parasite *Plasmodium falciparum*. BMC Genom..

[67] Wilde M.L., Triglia T., Marapana D., Thompson J.K., Kouzmitchev A.A., Bullen H.E., Gilson P.R., Cowman A.F., Tonkin C.J. (2019). Protein Kinase A Is Essential for Invasion of *Plasmodium falciparum* into Human Erythrocytes. mBio.

[68] Merckx A., Nivez M.P., Bouyer G., Alano P., Langsley G., Deitsch K., Thomas S., Doerig C., Egée S. (2008). *Plasmodium falciparum* regulatory subunit of cAMP-dependent PKA and anion channel conductance. PLoS Pathog..

[69] Wurtz N., Chapus C., Desplans J., Parzy D. (2011). cAMP-dependent protein kinase from *Plasmodium falciparum*: An update. Parasitology.

[70] Dorin D., Semblat J.P., Poullet P., Alano P., Goldring J.P., Whittle C., Patterson S., Chakrabarti D., Doerig C. (2005). PfPK7, an atypical MEK-related protein kinase, reflects the absence of classical three-component MAPK pathways in the human malaria parasite *Plasmodium falciparum*. Mol. Microbiol..

[71] Koyama F.C., Ribeiro R.Y., Garcia J.L., Azevedo M.F., Chakrabarti D., Garcia C.R. (2012). Ubiquitin proteasome system and the atypical kinase PfPK7 are involved in melatonin signaling in *Plasmodium falciparum*. J. Pineal. Res..

[72] Pease B.N., Huttlin E.L., Jedrychowski M.P., Dorin-Semblat D., Sebastiani D., Segarra D.T., Roberts B.F., Chakrabarti R., Doerig C., Gygi S.P. (2018). Characterization of *Plasmodium falciparum* Atypical Kinase PfPK7- Dependent Phosphoproteome. J. Proteome Res..

[73] Deshmukh A.S., Agarwal M., Mehra P., Gupta A., Gupta N., Doerig C.D., Dhar S.K. (2015). Regulation of *Plasmodium falciparum* Origin Recognition Complex subunit 1 (PfORC1) function through phosphorylation mediated by CDK-like kinase PK5. Mol. Microbiol..

[74] Zhang M., Mishra S., Sakthivel R., Rojas M., Ranjan R., Sullivan W.J., Fontoura B.M., Ménard R., Dever T.E., Nussenzweig V. (2012). PK4, a eukaryotic initiation factor 2α(eIF2α) kinase, is essential for the development of the erythrocytic cycle of Plasmodium. Proc. Natl. Acad. Sci. USA.

[75] Wernimont A.K., Artz J.D., Finerty P., Lin Y.H., Amani M., Allali-Hassani A., Senisterra G., Vedadi M., Tempel W., Mackenzie F. (2010). Structures of apicomplexan calcium-dependent protein kinases reveal mechanism of activation by calcium. Nat. Struct. Mol. Biol..

[76] Hopp C.S., Bowyer P.W., Baker D.A. (2012). The role of cGMP signalling in regulating life cycle progression of Plasmodium. Microbes Infect..

[77] Baker D.A., Stewart L.B., Large J.M., Bowyer P.W., Ansell K.H., Jiménez-Díaz M.B., El Bakkouri M., Birchall K., Dechering K.J., Bouloc N.S. (2017). A potent series targeting the malarial cGMP-dependent protein kinase clears infection and blocks transmission. Nat. Commun..

[78] Droucheau E., Primot A., Thomas V., Mattei D., Knockaert M., Richardson C., Sallicandro P., Alano P., Jafarshad A., Baratte B. (2004). *Plasmodium falciparum* glycogen synthase kinase-3: Molecular model, expression, intracellular localisation and selective inhibitors. Biochim. Biophys. Acta.

[79] Masch A., Nasereddin A., Alder A., Bird M.J., Schweda S.I., Preu L., Doerig C., Dzikowski R., Gilberger T.W., Kunick C. (2019). Structure-activity relationships in a series of antiplasmodial thieno [2,3-b]pyridines. Malar. J..

[80] Moolman C., van der Sluis R., Beteck R.M., Legoabe L.J. (2021). Exploration of benzofuran-based compounds as potent and selective *Plasmodium falciparum* glycogen synthase kinase-3 (PfGSK-3) inhibitors. Bioorg. Chem..

[81] Thomas D.C., Ahmed A., Gilberger T.W., Sharma P. (2012). Regulation of *Plasmodium falciparum* glideosome associated protein 45 (PfGAP45) phosphorylation. PLoS ONE.

[82] Green J.L., Rees-Channer R.R., Howell S.A., Martin S.R., Knuepfer E., Taylor H.M., Grainger M., Holder A.A. (2008). The motor complex of *Plasmodium falciparum*: Phosphorylation by a calcium-dependent protein kinase. J. Biol. Chem..

[83] Hitz E., Wiedemar N., Passecker A., Graça B.A.S., Scheurer C., Wittlin S., Brancucci N.M.B., Vakonakis I., Mäser P., Voss T.S. (2021). The 3-phosphoinositide-dependent protein kinase 1 is an essential upstream activator of protein kinase A in malaria parasites. PLoS Biol..

[84] Chandra B.R., Olivieri A., Silvestrini F., Alano P., Sharma A. (2005). Biochemical characterization of the two nucleosome assembly proteins from *Plasmodium falciparum*. Mol. Biochem. Parasitol..

[85] Barik S., Taylor R.E., Chakrabarti D. (1997). Identification, cloning, and mutational analysis of the casein kinase 1 cDNA of the malaria parasite, *Plasmodium falciparum*. Stage-specific expression of the gene. J. Biol. Chem..

[86] Dastidar E.G., Dayer G., Holland Z.M., Dorin-Semblat D., Claes A., Chêne A., Sharma A., Hamelin R., Moniatte M., Lopez-Rubio J.J. (2012). Involvement of *Plasmodium falciparum* protein kinase CK2 in the chromatin assembly pathway. BMC Biol..

[87] Dehury B., Behera S.K., Mahapatra N. (2017). Structural dynamics of Casein Kinase I (CKI) from malarial parasite *Plasmodium falciparum* (Isolate 3D7): Insights from theoretical modelling and molecular simulations. J. Mol. Graph. Model..

[88] Hitz E., Grüninger O., Passecker A., Wyss M., Scheurer C., Wittlin S., Beck H.P., Brancucci N.M.B., Voss T.S. (2021). The catalytic subunit of *Plasmodium falciparum* casein kinase 2 is essential for gametocytogenesis. Commun. Biol..

[89] Treeck M., Sanders J.L., Elias J.E., Boothroyd J.C. (2011). The phosphoproteomes of *Plasmodium falciparum* and Toxoplasma gondii reveal unusual adaptations within and beyond the parasites’ boundaries. Cell Host Microbe.

[90] Watzlowik M.T., Das S., Meissner M., Längst G. (2021). Peculiarities of *Plasmodium falciparum* Gene Regulation and Chromatin Structure. Int. J. Mol. Sci..

[91] Hora R., Bridges D.J., Craig A., Sharma A. (2009). Erythrocytic casein kinase II regulates cytoadherence of *Plasmodium falciparum*-infected red blood cells. J. Biol. Chem..

[92] Doerig C., Endicott J., Chakrabarti D. (2002). Cyclin-dependent kinase homologues of *Plasmodium falciparum*. Int. J. Parasitol..

[93] Bracchi-Ricard V., Barik S., Delvecchio C., Doerig C., Chakrabarti R., Chakrabarti D. (2000). PfPK6, a novel cyclin-dependent kinase/mitogen-activated protein kinase-related protein kinase from *Plasmodium falciparum*. Biochem. J..

[94] Le Roch K., Sestier C., Dorin D., Waters N., Kappes B., Chakrabarti D., Meijer L., Doerig C. (2000). Activation of a *Plasmodium falciparum* cdc2-related kinase by heterologous p25 and cyclin H. Functional characterization of a *P. falciparum* cyclin homologue. J. Biol. Chem..

[95] Merckx A., Le Roch K., Nivez M.P., Dorin D., Alano P., Gutierrez G.J., Nebreda A.R., Goldring D., Whittle C., Patterson S. (2003). Identification and initial characterization of three novel cyclin-related proteins of the human malaria parasite *Plasmodium falciparum*. J. Biol. Chem..

[96] Machado M., Klaus S., Klaschka D., Guizetti J., Ganter M. (2023). *Plasmodium falciparum* CRK4 links early mitotic events to the onset of S-phase during schizogony. mBio.

[97] Geyer J.A., Keenan S.M., Woodard C.L., Thompson P.A., Gerena L., Nichols D.A., Gutteridge C.E., Waters N.C. (2009). Selective inhibition of Pfmrk, a *Plasmodium falciparum* CDK.; by antimalarial 1,3-diaryl-2-propenones. Bioorg. Med. Chem. Lett..

[98] Carvalho T.G., Doerig C., Reininger L. (2013). Nima- and Aurora-related kinases of malaria parasites. Biochim. Biophys. Acta.

[99] Dorin-Semblat D., Quashie N., Halbert J., Sicard A., Doerig C., Peat E., Ranford-Cartwright L., Doerig C. (2007). Functional characterization of both MAP kinases of the human malaria parasite *Plasmodium falciparum* by reverse genetics. Mol. Microbiol..

[100] Rangarajan R., Bei A.K., Jethwaney D., Maldonado P., Dorin D., Sultan A.A., Doerig C. (2005). A mitogen-activated protein kinase regulates male gametogenesis and transmission of the malaria parasite *Plasmodium berghei*. EMBO Rep..

[101] Hitz E., Balestra A.C., Brochet M., Voss T.S. (2020). PfMAP-2 is essential for male gametogenesis in the malaria parasite *Plasmodium falciparum*. Sci. Rep..

[102] Siddiqui G., Proellochs N.I., Cooke B.M. (2020). Identification of essential exported *Plasmodium falciparum* protein kinases in malaria-infected red blood cells. Br. J. Haematol..

[103] Anil Kumar D., Shrivastava D., Sahasrabuddhe A.A., Habib S., Trivedi V. (2021). *Plasmodium falciparum* FIKK9.1 is a monomeric serine-threonine protein kinase with features to exploit as a drug target. Chem. Biol. Drug Des..

[104] Ekka R., Gupta A., Bhatnagar S., Malhotra P., Sharma P. (2020). Phosphorylation of Rhoptry Protein RhopH3 Is Critical for Host Cell Invasion by the Malaria Parasite. mBio.

[105] Dixit A., Singh P.K., Sharma G.P., Malhotra P., Sharma P. (2010). PfSRPK1, a novel splicing-related kinase from *Plasmodium falciparum*. J. Biol. Chem..

[106] Pandey R., Mohmmed A., Pierrot C., Khalife J., Malhotra P., Gupta D. (2014). Genome wide in silico analysis of *Plasmodium falciparum* phosphatome. BMC Genom..

[107] Kumar R., Adams B., Oldenburg A., Musiyenko A., Barik S. (2002). Characterisation and expression of a PP1 serine/threonine protein phosphatase (PfPP1) from the malaria parasite, *Plasmodium falciparum*: Demonstration of its essential role using RNA interference. Malar. J..

[108] Ali F., Wali H., Jan S., Zia A., Aslam M., Ahmad I., Afridi S.G., Shams S., Khan A. (2021). Analysing the essential proteins set of *Plasmodium falciparum* PF3D7 for novel drug targets identification against malaria. Malar. J..

[109] Philip N., Waters A.P. (2015). Conditional Degradation of Plasmodium Calcineurin Reveals Functions in Parasite Colonization of both Host and Vector. Cell Host Microbe.

[110] Kumar R., Musiyenko A., Barik S. (2005). *Plasmodium falciparum* calcineurin and its association with heat shock protein 90: Mechanisms for the antimalarial activity of cyclosporin A and synergism with geldanamycin. Mol. Biochem. Parasitol..

[111] Jahangiri F., Jalallou N., Ebrahimi M. (2019). Analysis of Apical Membrane Antigen (AMA)-1 characteristics using bioinformatics tools in order to vaccine design against *Plasmodium vivax*. Infect. Genet. Evol..

[112] Miao J., Lawrence M., Jeffers V., Zhao F., Parker D., Ge Y., Sullivan W.J., Cui L. (2013). Extensive lysine acetylation occurs in evolutionarily conserved metabolic pathways and parasite-specific functions during *Plasmodium falciparum* intraerythrocytic development. Mol. Microbiol..

[113] Trelle M.B., Salcedo-Amaya A.M., Cohen A.M., Stunnenberg H.G., Jensen O.N. (2009). Global histone analysis by mass spectrometry reveals a high content of acetylated lysine residues in the malaria parasite *Plasmodium falciparum*. J. Proteome Res..

[114] Connacher J., von Grüning H., Birkholtz L. (2022). Histone Modification Landscapes as a Roadmap for Malaria Parasite Development. Front. Cell Dev. Biol..

[115] Coleman B.I., Skillman K.M., Jiang R.H.Y., Childs L.M., Altenhofen L.M., Ganter M., Leung Y., Goldowitz I., Kafsack B.F.C., Marti M. (2014). A *Plasmodium falciparum* histone deacetylase regulates antigenic variation and gametocyte conversion. Cell Host Microbe.

[116] Mancio-Silva L., Lopez-Rubio J.J., Claes A., Scherf A. (2013). Sir2a regulates rDNA transcription and multiplication rate in the human malaria parasite *Plasmodium falciparum*. Nat. Commun..

[117] Kanyal A., Rawat M., Gurung P., Choubey D., Anamika K., Karmodiya K. (2018). Genome-wide survey and phylogenetic analysis of histone acetyltransferases and histone deacetylases of *Plasmodium falciparum*. FEBS J..

[118] Lopez-Rubio J.J., Mancio-Silva L., Scherf A. (2009). Genome-wide analysis of heterochromatin associates clonally variant gene regulation with perinuclear repressive centers in malaria parasites. Cell Host Microbe.

[119] Miao J., Wang C., Lucky A.B., Liang X., Min H., Adapa S.R., Jiang R., Kim K., Cui L. (2021). A unique GCN5 histone acetyltransferase complex controls erythrocyte invasion and virulence in the malaria parasite *Plasmodium falciparum*. PLoS Pathog..

[120] Shekhar S., Bhowmick K., Dhar S.K. (2022). Role of PfMYST in DNA replication in *Plasmodium falciparum*. Exp. Parasitol..

[121] Zeeshan M., Kaur I., Joy J., Saini E., Paul G., Kaushik A., Dabral S., Mohmmed A., Gupta D., Malhotra P. (2017). Proteomic Identification and Analysis of Arginine-Methylated Proteins of *Plasmodium falciparum* at Asexual Blood Stages. J. Proteome Res..

[122] Fan Q., Miao J., Cui L., Cui L. (2009). Characterization of PRMT1 from *Plasmodium falciparum*. Biochem. J..

[123] Volz J.C., Bártfai R., Petter M., Langer C., Josling G.A., Tsuboi T., Schwach F., Baum J., Rayner J.C., Stunnenberg H.G. (2012). PfSET10, a *Plasmodium falciparum* methyltransferase, maintains the active var gene in a poised state during parasite division. Cell Host Microbe.

[124] Jiang L., Mu J., Zhang Q., Ni T., Srinivasan P., Rayavara K., Yang W., Turner L., Lavstsen T., Theander T.G. (2013). PfSETvs methylation of histone H3K36 represses virulence genes in *Plasmodium falciparum*. Nature.

[125] Cao X., Wen Y., Li Y., Ma X., Jing Q., Jiang L., Wei G. (2023). PfSET2 Is Involved in Genome Organization of Var Gene Family in *Plasmodium falciparum*. Microbiol. Spectr..

[126] Kaur I., Zeeshan M., Saini E., Kaushik A., Mohmmed A., Gupta D., Malhotra P. (2016). Widespread occurrence of lysine methylation in *Plasmodium falciparum* proteins at asexual blood stages. Sci. Rep..

[127] Hossain M., Sharma S., Korde R., Kanodia S., Chugh M., Rawat K., Malhotra P. (2013). Organization of *Plasmodium falciparum* spliceosomal core complex and role of arginine methylation in its assembly. Malar. J..

[128] Cui L., Fan Q., Cui L., Miao J. (2008). Histone lysine methyltransferases and demethylases in *Plasmodium falciparum*. Int. J. Parasitol..

[129] Matthews K.A., Senagbe K.M., Nötzel C., Gonzales C.A., Tong X., Rijo-Ferreira F., Bhanu N.V., Miguel-Blanco C., Lafuente-Monasterio M.J., Garcia B.A. (2020). Disruption of the *Plasmodium falciparum* Life Cycle through Transcriptional Reprogramming by Inhibitors of Jumonji Demethylases. ACS Infect. Dis..

[130] Rosenthal P.J. (2004). Cysteine proteases of malaria parasites. Int. J. Parasitol..

[131] Rosenthal P.J. (2020). Falcipain cysteine proteases of malaria parasites: An update. Biochim. Biophys. Acta Proteins Proteom..

[132] Pandey K.C., Dixit R. (2012). Structure-function of falcipains: Malarial cysteine proteases. J. Trop. Med..

[133] Pei Y., Miller J.L., Lindner S.E., Vaughan A.M., Torii M., Kappe S.H.I. (2013). Plasmodium yoelii inhibitor of cysteine proteases is exported to exomembrane structures and interacts with yoelipain-2 during asexual blood-stage development. Cell. Microbiol..

[134] Patra J., Rana D., Arora S., Pal M., Mahindroo N. (2023). Falcipains: Biochemistry, target validation and structure-activity relationship studies of inhibitors as antimalarials. Eur. J. Med. Chem..

[135] Lehmann C., Tan M.S.Y., de Vries L.E., Russo I., Sanchez M.I., Goldberg D.E., Deu E. (2018). *Plasmodium falciparum* dipeptidyl aminopeptidase 3 activity is important for efficient erythrocyte invasion by the malaria parasite. PLoS Pathog..

[136] Gomes M.M., Budu A., Ventura P.D., Bagnaresi P., Cotrin S.S., Cunha R.L., Carmona A.K., Juliano L., Gazarini M.L. (2015). Specific calpain activity evaluation in Plasmodium parasites. Anal. Biochem..

[137] Mukherjee S., Nasamu A.S., Rubiano K.C., Goldberg D.E. (2023). Activation of the *Plasmodium* Egress Effector Subtilisin-Like Protease 1 Is Mediated by Plasmepsin X Destruction of the Prodomain. mBio.

[138] Lemberg M.K., Freeman M. (2007). Functional and evolutionary implications of enhanced genomic analysis of rhomboid intramembrane proteases. Genome Res..

[139] Vera I.M., Beatty W.L., Sinnis P., Kim K. (2011). Plasmodium protease ROM1 is important for proper formation of the parasitophorous vacuole. PLoS Pathog..

[140] Baker R.P., Wijetilaka R., Urban S. (2006). Two Plasmodium rhomboid proteases preferentially cleave different adhesins implicated in all invasive stages of malaria. PLoS Pathog..

[141] Sleebs B.E., Lopaticki S., Marapana D.S., O’Neill M.T., Rajasekaran P., Gazdik M., Günther S., Whitehead L.W., Lowes K.N., Barfod L. (2014). Inhibition of Plasmepsin V activity demonstrates its essential role in protein export, PfEMP1 display, and survival of malaria parasites. PLoS Biol..

[142] Nasamu A.S., Polino A.J., Istvan E.S., Goldberg D.E. (2020). Malaria parasite plasmepsins: More than just plain old degradative pepsins. J. Biol. Chem..

[143] Bhaumik P., Xiao H., Parr C.L., Kiso Y., Gustchina A., Yada R.Y., Wlodawer A. (2009). Crystal structures of the histo-aspartic protease (HAP) from *Plasmodium falciparum*. J. Mol. Biol..

[144] Murata C.E., Goldberg D.E. (2003). *Plasmodium falciparum* falcilysin: A metalloprotease with dual specificity. J. Biol. Chem..

[145] Drinkwater N., Sivaraman K.K., Bamert R.S., Rut W., Mohamed K., Vinh N.B., Scammells P.J., Drag M., McGowan S. (2016). Structure and substrate fingerprint of aminopeptidase P from *Plasmodium falciparum*. Biochem. J..

[146] Li X., Chen H., Oh S.S., Chishti A.H. (2008). A Presenilin-like protease associated with *Plasmodium falciparum* micronemes is involved in erythrocyte invasion. Mol. Biochem. Parasitol..

[147] Lauterbach S.B., Coetzer T.L. (2008). The M18 aspartyl aminopeptidase of *Plasmodium falciparum* binds to human erythrocyte spectrin in vitro. Malar. J..

[148] Akman-Anderson L., Olivier M., Luckhart S. (2007). Induction of nitric oxide synthase and activation of signaling proteins in Anopheles mosquitoes by the malaria pigment, hemozoin. Infect. Immunol..

[149] Kumar A., Singh K.P., Bali P., Anwar S., Kaul A., Singh O.P., Gupta B.K., Kumari N., Noor Alam M., Raziuddin M. (2018). iNOS polymorphism modulates iNOS/NO expression via impaired antioxidant and ROS content in *P. vivax* and *P. falciparum* infection. Redox Biol..

[150] Torgler R., Bongfen S.E., Romero J.C., Tardivel A., Thome M., Corradin G. (2008). Sporozoite-mediated hepatocyte wounding limits Plasmodium parasite development via MyD88-mediated NF-kappa B activation and inducible NO synthase expression. J. Immunol..

[151] Kordes M., Ormond L., Rausch S., Matuschewski K., Hafalla J.C.R. (2022). TLR9 signalling inhibits Plasmodium liver infection by macrophage activation. Eur. J. Immunol..

[152] Skorokhod O.A., Schwarzer E., Ceretto M., Arese P. (2007). Malarial pigment haemozoin, IFN-gamma, TNF-alpha, IL-1beta and LPS do not stimulate expression of inducible nitric oxide synthase and production of nitric oxide in immuno-purified human monocytes. Malar. J..

[153] Wang L., Delahunty C., Prieto J.H., Rahlfs S., Jortzik E., Yates J.R., Becker K. (2014). Protein S-nitrosylation in *Plasmodium falciparum*. Antioxid. Redox Signal..

[154] Kimura E.A., Couto A.S., Peres V.J., Casal O.L., Katzin A.M. (1996). N-linked glycoproteins are related to schizogony of the intraerythrocytic stage in *Plasmodium falciparum*. J. Biol. Chem..

[155] Gowda D.C., Miller L.H. (2024). Glycosylation in malaria parasites: What do we know?. Trends. Parasitol..

[156] Dieckmann-Schuppert A., Bause E., Schwarz R.T. (1994). Glycosylation reactions in *Plasmodium falciparum*, Toxoplasma gondii, and Trypanosoma brucei brucei probed by the use of synthetic peptides. Biochim. Biophys. Acta.

[157] Bandini G., Albuquerque-Wendt A., Hegermann J., Samuelson J., Routier F.H. (2019). Protein *O*- and *C*-Glycosylation pathways in *Toxoplasma gondii* and *Plasmodium falciparum*. Parasitology.

[158] Tajik S., Sadeghi S., Iravani A., Khalili M., Arjmand M., Din N.U., Vahabi F., Feiz-Haddad H., Lame-Rad B., Naddaf S.R. (2019). Characterization of Glycoproteins of Native 19kDa C-Terminal Merozoite Surface Protein-1 from Native Antigen of *Plasmodium falciparum*. J. Arthropod. Borne Dis..

[159] Kehr S., Jortzik E., Delahunty C., Yates J.R., Rahlfs S., Becker K. (2011). Protein S-glutathionylation in malaria parasites. Antioxid. Redox Signal..

[160] Schlott A.C., Knuepfer E., Green J.L., Hobson P., Borg A.J., Morales-Sanfrutos J., Perrin A.J., Maclachlan C., Collinson L.M., Snijders A.P. (2021). Inhibition of protein N-myristoylation blocks *Plasmodium falciparum* intraerythrocytic development, egress and invasion. PLoS Biol..

[161] Counihan N.A., Chernih H.C., de Koning-Ward T.F. (2022). Post-translational lipid modifications in Plasmodium parasites. Curr. Opin. Microbiol..

[162] Rees-Channer R.R., Martin S.R., Green J.L., Bowyer P.W., Grainger M., Molloy J.E., Holder A.A. (2006). Dual acylation of the 45 kDa gliding-associated protein (GAP45) in *Plasmodium falciparum* merozoites. Mol. Biochem. Parasitol..

[163] Jones M.L., Collins M.O., Goulding D., Choudhary J.S., Rayner J.C. (2012). Analysis of protein palmitoylation reveals a pervasive role in Plasmodium development and pathogenesis. Cell Host Microbe.

[164] Cabrera A., Herrmann S., Warszta D., Santos J.M., John Peter A.T., Kono M., Debrouver S., Jacobs T., Spielmann T., Ungermann C. (2012). Dissection of minimal sequence requirements for rhoptry membrane targeting in the malaria parasite. Traffic.

[165] Geiger M., Brown C., Wichers J.S., Strauss J., Lill A., Thuenauer R., Liffner B., Wilcke L., Lemcke S., Heincke D. (2020). Structural Insights Into PfARO and Characterization of its Interaction With PfAIP. J. Mol. Biol..

[166] Das S., Hertrich N., Perrin A.J., Withers-Martinez C., Collins C.R., Jones M.L., Watermeyer J.M., Fobes E.T., Martin S.R., Saibil H.R. (2015). Processing of *Plasmodium falciparum* Merozoite Surface Protein MSP1 Activates a Spectrin-Binding Function Enabling Parasite Egress from RBCs. Cell Host Microbe.

[167] Egan A.F., Morris J., Barnish G., Allen S., Greenwood B.M., Kaslow D.C., Holder A.A., Riley E.M. (1996). Clinical immunity to *Plasmodium falciparum* malaria is associated with serum antibodies to the 19-kDa C-terminal fragment of the merozoite surface antigen, PfMSP-1. J. Infect. Dis..

[168] Sanchez C.P., Moliner Cubel S., Nyboer B., Jankowska-Döllken M., Schaeffer-Reiss C., Ayoub D., Planelles G., Lanzer M. (2019). Phosphomimetic substitution at Ser-33 of the chloroquine resistance transporter PfCRT reconstitutes drug responses in *Plasmodium falciparum*. J. Biol. Chem..

[169] Hodson N., Invergo B., Rayner J.C., Choudhary J.S. (2015). Palmitoylation and palmitoyl-transferases in Plasmodium parasites. Biochem. Soc. Trans..

[170] Frénal K., Tay C.L., Mueller C., Bushell E.S., Jia Y., Graindorge A., Billker O., Rayner J.C., Soldati-Favre D. (2013). Global analysis of apicomplexan protein S-acyl transferases reveals an enzyme essential for invasion. Traffic.

[171] Qian P., Wang X., Zhong C.Q., Wang J., Cai M., Nguitragool W., Li J., Cui H., Yuan J. (2022). Inner membrane complex proteomics reveals a palmitoylation regulation critical for intraerythrocytic development of malaria parasite. eLife.

[172] Suazo K.F., Schaber C., Palsuledesai C.C., Odom John A.R., Distefano M.D. (2016). Global proteomic analysis of prenylated proteins in *Plasmodium falciparum* using an alkyne-modified isoprenoid analogue. Sci. Rep..

[173] Jung D., Bachmann H.S. (2023). Regulation of protein prenylation. Biomed. Pharmacother..

[174] Chakrabarti D., Azam T., DelVecchio C., Qiu L., Park Y.I., Allen C.M. (1998). Protein prenyl transferase activities of *Plasmodium falciparum*. Mol. Biochem. Parasitol..

[175] Wright M.H., Clough B., Rackham M.D., Rangachari K., Brannigan J.A., Grainger M., Moss D.K., Bottrill A.R., Heal W.P., Broncel M. (2014). Validation of N-myristoyltransferase as an antimalarial drug target using an integrated chemical biology approach. Nat. Chem..

[176] Howe R., Kelly M., Jimah J., Hodge D., Odom A.R. (2013). Isoprenoid biosynthesis inhibition disrupts Rab5 localization and food vacuolar integrity in *Plasmodium falciparum*. Eukaryot. Cell.

[177] Ayong L., DaSilva T., Mauser J., Allen C.M., Chakrabarti D. (2011). Evidence for prenylation-dependent targeting of a Ykt6 SNARE in *Plasmodium falciparum*. Mol. Biochem. Parasitol..

[178] Mayer D.C.G. (2021). Protein Sorting in *Plasmodium falciparum*. Life.

[179] Gisselberg J.E., Zhang L., Elias J.E., Yeh E. (2017). The Prenylated Proteome of *Plasmodium falciparum* Reveals Pathogen-specific Prenylation Activity and Drug Mechanism-of-action. Mol. Cell. Proteom..

[180] Chakrabarti D., Da Silva T., Barger J., Paquette S., Patel H., Patterson S., Allen C.M. (2002). Protein farnesyltransferase and protein prenylation in *Plasmodium falciparum*. J. Biol. Chem..

[181] Rzepczyk C.M., Saul A.J., Ferrante A. (1984). Polyamine oxidase-mediated intraerythrocytic killing of *Plasmodium falciparum*: Evidence against the role of reactive oxygen metabolites. Infect. Immunol..

[182] Pabón A., Carmona J., Burgos L.C., Blair S. (2003). Oxidative stress in patients with non-complicated malaria. Clin. Biochem..

[183] Schwarzer E., Arese P., Skorokhod O.A. (2015). Role of the lipoperoxidation product 4-hydroxynonenal in the pathogenesis of severe malaria anemia and malaria immunodepression. Oxid. Med. Cell. Longev..

[184] Mueangson O., Mahittikorn A., Anabire N.G., Mala W., Kotepui M. (2023). Increased Blood Concentrations of Malondialdehyde in Plasmodium Infection: A Systematic Review and Meta-Analysis. Antioxidants.

[185] Muller S. (2004). Redox and antioxidant systems of the malaria parasite *Plasmodium falciparum*. Mol. Microbiol..

[186] Bozdech Z., Ginsburg H. (2004). Antioxidant defense in *Plasmodium falciparum*—Data mining of the transcriptome. Malar. J..

[187] Hain A.U., Bosch J. (2013). Autophagy in Plasmodium, a multifunctional pathway?. Comput. Struct. Biotechnol. J..

[188] Ponder E.L., Bogyo M. (2007). Ubiquitin-like modifiers and their deconjugating enzymes in medically important parasitic protozoa. Eukaryot. Cell.

[189] Chung D.W., Ponts N., Prudhomme J., Rodrigues E.M., Le Roch K.G. (2012). Characterization of the ubiquitylating components of the human malaria parasite’s protein degradation pathway. PLoS ONE.

[190] Sumam de Oliveira D., Kronenberger T., Palmisano G., Wrenger C., de Souza E.E. (2021). Targeting SUMOylation in Plasmodium as a Potential Target for Malaria Therapy. Front. Cell. Infect. Microbiol..

[191] Mata-Cantero L., Azkargorta M., Aillet F., Xolalpa W., LaFuente M.J., Elortza F., Carvalho A.S., Martin-Plaza J., Matthiesen R., Rodriguez M.S. (2016). New insights into host-parasite ubiquitin proteome dynamics in *P. falciparum* infected red blood cells using a TUBEs-MS approach. J. Proteom..

[192] Ponts N., Saraf A., Chung D.W., Harris A., Prudhomme J., Washburn M.P., Florens L., Le Roch K.G. (2011). Unraveling the ubiquitome of the human malaria parasite. J. Biol. Chem..

[193] Karpiyevich M., Adjalley S., Mol M., Ascher D.B., Mason B., van der Heden van Noort G.J., Laman H., Ovaa H., Lee M.C.S., Artavanis-Tsakonas K. (2019). Nedd8 hydrolysis by UCH proteases in Plasmodium parasites. PLoS Pathog..

[194] Philip N., Haystead T.A. (2007). Characterization of a UBC13 kinase in *Plasmodium falciparum*. Proc. Natl. Acad. Sci. USA.

[195] Narwal S.K., Nayak B., Mehra P., Mishra S. (2022). Protein kinase 9 is not required for completion of the *Plasmodium berghei* life cycle. Microbiol. Res..

[196] Pisciotta J.M., Scholl P.F., Shuman J.L., Shualev V., Sullivan D.J. (2017). Quantitative characterization of hemozoin in *Plasmodium berghei* and vivax. Int. J. Parasitol. Drugs Drug Resist..

[197] Schwarzer E., Turrini F., Arese P. (1994). A luminescence method for the quantitative determination of phagocytosis of erythrocytes, of malaria-parasitized erythrocytes and of malarial pigment. Br. J. Haematol..

[198] Deroost K., Lays N., Noppen S., Martens E., Opdenakker G., Van den Steen P.E. (2012). Improved methods for haemozoin quantification in tissues yield organ-and parasite-specific information in malaria-infected mice. Malar. J..

[199] Thamarath S.S., Xiong A., Lin P.H., Preiser P.R., Han J. (2019). Enhancing the sensitivity of micro magnetic resonance relaxometry detection of low parasitemia *Plasmodium falciparum* in human blood. Sci. Rep..

[200] Di Gregorio E., Ferrauto G., Schwarzer E., Gianolio E., Valente E., Ulliers D., Aime S., Skorokhod O. (2020). Relaxometric studies of erythrocyte suspensions infected by *Plasmodium falciparum*: A tool for staging infection and testing anti-malarial drugs. Magn. Reason. Med..

[201] Karl S., Gutiérrez L., House M.J., Davis T.M., St Pierre T.G. (2011). Nuclear magnetic resonance: A tool for malaria diagnosis?. Am. J. Trop. Med. Hyg..

[202] Poli G., Schaur R.J., Siems W.G., Leonarduzzi G. (2008). 4-Hydroxynonenal: A membrane lipid oxidation product of medicinal interest. Med. Res. Rev..

[203] Uchida K., Stadtman E.R. (1992). Modification of histidine residues in proteins by reaction with 4-hydroxynonenal. Proc. Natl. Acad. Sci. USA.

[204] Schaur R.J. (2003). Basic aspects of the biochemical reactivity of 4-hydroxynonenal. Mol. Asp. Med..

[205] Viedma-Poyatos Á., González-Jiménez P., Langlois O., Company-Marín I., Spickett C.M., Pérez-Sala D. (2021). Protein Lipoxidation: Basic Concepts and Emerging Roles. Antioxidants.

[206] Buffinton G.D., Hunt N.H., Cowden W.B., Clark I.A. (1988). Detection of short-chain carbonyl products of lipid peroxidation from malaria-parasite (Plasmodium vinckei)-infected red blood cells exposed to oxidative stress. Biochem. J..

[207] Olivier M., Van Den Ham K., Shio M.T., Kassa F.A., Fougeray S. (2014). Malarial pigment hemozoin and the innate inflammatory response. Front. Immunol..

[208] Uyoga S., Skorokhod O.A., Opiyo M., Orori E.N., Williams T.N., Arese P., Schwarzer E. (2012). Transfer of 4-hydroxynonenal from parasitized to non-parasitized erythrocytes in rosettes. Proposed role in severe malaria anemia. Br. J. Haematol..

[209] Aguilar R., Marrocco T., Skorokhod O.A., Barbosa A., Nhabomba A., Manaca M.N., Guinovart C., Quintó L., Arese P., Alonso P.L. (2014). Blood oxidative stress markers and *Plasmodium falciparum* malaria in non-immune African children. Br. J. Haematol..

[210] Zhang G., Skorokhod O.A., Khoo S.K., Aguilar R., Wiertsema S., Nhabomba A.J., Marrocco T., McNamara-Smith M., Manaca M.N., Barbosa A. (2014). Plasma advanced oxidative protein products are associated with anti-oxidative stress pathway genes and malaria in a longitudinal cohort. Malar. J..

[211] Vennerstrom J.L., Eaton J.W. (1988). Oxidants, oxidant drugs, and malaria. J. Med. Chem..

[212] Wagner M.P., Chitnis C.E. (2023). Lipid peroxidation and its repair in malaria parasites. Trends. Parasitol..

[213] Armstrong J.F., Campo B., Alexander S.P.H., Arendse L.B., Cheng X., Davenport A.P., Faccenda E., Fidock D.A., Godinez-Macias K.P., Harding S.D. (2023). Advances in malaria pharmacology and the online guide to MALARIA PHARMACOLOGY: IUPHAR review 38. Br. J. Pharmacol..

[214] Barrera V., Skorokhod O.A., Baci D., Gremo G., Arese P., Schwarzer E. (2011). Host fibrinogen stably bound to hemozoin rapidly activates monocytes via TLR-4 and CD11b/CD18-integrin: A new paradigm of hemozoin action. Blood.

[215] Schwarzer E., Turrini F., Ulliers D., Giribaldi G., Ginsburg H., Arese P. (1992). Impairment of macrophage functions after ingestion of *Plasmodium falciparum*-infected erythrocytes or isolated malarial pigment. J. Exp. Med..

[216] Metzger W.G., Mordmüller B.G., Kremsner P.G. (1995). Malaria pigment in leucocytes. Trans. R. Soc. Trop. Med. Hyg..

[217] Gallo V., Skorokhod O.A., Schwarzer E., Arese P. (2012). Simultaneous determination of phagocytosis of *Plasmodium falciparum*-parasitized and non-parasitized red blood cells by flow cytometry. Malar. J..

[218] Skorokhod O.A., Alessio M., Mordmüller B., Arese P., Schwarzer E. (2004). Hemozoin (malarial pigment) inhibits differentiation and maturation of human monocyte-derived dendritic cells: A peroxisome proliferator-activated receptor-gamma-mediated effect. J. Immunol..

[219] Urban B.C., Todryk S. (2006). Malaria pigment paralyzes dendritic cells. J. Biol..

[220] Hänscheid T., Egan T.J., Grobusch M.P. (2007). Haemozoin: From melatonin pigment to drug target, diagnostic tool, and immune modulator. Lancet Infect. Dis..

[221] Schwarzer E., Skorokhod O.A., Barrera V., Arese P. (2008). Hemozoin and the human monocyte—A brief review of their interactions. Parassitologia.

[222] Skorokhod O.A., Barrera V., Heller R., Carta F., Turrini F., Arese P., Schwarzer E. (2014). Malarial pigment hemozoin impairs chemotactic motility and transendothelial migration of monocytes via 4-hydroxynonenal. Free Radic. Biol. Med..

[223] Skorokhod O., Barrera V., Mandili G., Costanza F., Valente E., Ulliers D., Schwarzer E. (2021). Malaria Pigment Hemozoin Impairs GM-CSF Receptor Expression and Function by 4-Hydroxynonenal. Antioxidants.

[224] Skorokhod O., Triglione V., Barrera V., Di Nardo G., Valente E., Ulliers D., Schwarzer E., Gilardi G. (2023). Posttranslational Modification of Human Cytochrome CYP4F11 by 4-Hydroxynonenal Impairs ω-Hydroxylation in Malaria Pigment Hemozoin-Fed Monocytes: The Role in Malaria Immunosuppression. Int. J. Mol. Sci..

[225] Cambos M., Bazinet S., Abed E., Sanchez-Dardon J., Bernard C., Moreau R., Olivier M., Scorza T. (2010). The IL-12p70/IL-10 interplay is differentially regulated by free heme and hemozoin in murine bone-marrow-derived macrophages. Int. J. Parasitol..

[226] Bujila I., Schwarzer E., Skorokhod O., Weidner J.M., Troye-Blomberg M., Östlund Farrants A.K. (2016). Malaria-derived hemozoin exerts early modulatory effects on the phenotype and maturation of human dendritic cells. Cell. Microbiol..

[227] Lasaviciute G., Tariq K., Sugathan A., Quin J.E., Bujila I., Skorokhod O., Troye-Blomberg M., Sverremark-Ekstrom E., Farrants A.-K.O. (2024). Malaria-derived hemozoin alters chromatin remodelling and skews dendritic cell responses to subsequent bacterial infections. bioRxiv.

[228] Skorokhod O.A., Caione L., Marrocco T., Migliardi G., Barrera V., Arese P., Piacibello W., Schwarzer E. (2010). Inhibition of erythropoiesis in malaria anemia: Role of hemozoin and hemozoin-generated 4-hydroxynonenal. Blood.

[229] Dumarchey A., Lavazec C., Verdier F. (2022). Erythropoiesis and Malaria, a Multifaceted Interplay. Int. J. Mol. Sci..

[230] Schwarzer E., Müller O., Arese P., Siems W.G., Grune T. (1996). Increased levels of 4-hydroxynonenal in human monocytes fed with malarial pigment hemozoin. A possible clue for hemozoin toxicity. FEBS Lett..

[231] Mahmud F., Lee P.C., Abdul Wahab H., Mustaffa K.M.F., Leow C.H., Azhar R., Lai N.S. (2020). *Plasmodium falciparum* protein kinase as a potential therapeutic target for antimalarial drugs development. Trop. Biomed..

[232] Alder A., Wilcke L., Pietsch E., von Thien H., Pazicky S., Löw C., Mesen-Ramirez P., Bachmann A., Burda P.C., Kunick C. (2022). Functional inactivation of *Plasmodium falciparum* glycogen synthase kinase GSK3 modulates erythrocyte invasion and blocks gametocyte maturation. J. Biol. Chem..

[233] Blomqvist K., Helmel M., Wang C., Absalon S., Labunska T., Rudlaff R.M., Adapa S., Jiang R., Steen H., Dvorin J.D. (2020). Influence of *Plasmodium falciparum* Calcium-Dependent Protein Kinase 5 (PfCDPK5) on the Late Schizont Stage Phosphoproteome. mSphere.

[234] Jeninga M.D., Quinn J.E., Petter M. (2019). ApiAP2 Transcription Factors in Apicomplexan Parasites. Pathogens.

[235] Reyser T., Paloque L., Augereau J.M., Di Stefano L., Benoit-Vical F. (2024). Epigenetic regulation as a therapeutic target in the malaria parasite *Plasmodium falciparum*. Malar. J..

[236] López-Gutiérrez B., Cova M., Izquierdo L.A. (2019). *Plasmodium falciparum* C-mannosyltransferase is dispensable for parasite asexual blood stage development. Parasitology.

[237] Kupferschmid M., Aquino-Gil M.O., Shams-Eldin H., Schmidt J., Yamakawa N., Krzewinski F., Schwarz R.T., Lefebvre T. (2017). Identification of O-GlcNAcylated proteins in *Plasmodium falciparum*. Malar. J..

[238] Howell S.A., Well I., Fleck S.L., Kettleborough C., Collins C.R., Blackman M.J. (2003). A single malaria merozoite serine protease mediates shedding of multiple surface proteins by juxtamembrane cleavage. J. Biol. Chem..

[239] Collins C.R., Hackett F., Howell S.A., Snijders A.P., Russell M.R., Collinson L.M., Blackman M.J. (2020). The malaria parasite sheddase SUB2 governs host red blood cell membrane sealing at invasion. eLife.

[240] Kumar S., Gargaro O.R., Kappe S.H.I. (2022). *Plasmodium falciparum* CRK5 Is Critical for Male Gametogenesis and Infection of the Mosquito. mBio.

[241] Renn J.P., Doritchamou J.Y.A., Tentokam B.C.N., Morrison R.D., Cowles M.V., Burkhardt M., Ma R., Mahamar A., Attaher O., Diarra B.S. (2021). Allelic variants of full-length VAR2CSA.; the placental malaria vaccine candidate, differ in antigenicity and receptor binding affinity. Commun. Biol..

[242] Kennedy M.C., Wang J., Zhang Y., Miles A.P., Chitsaz F., Saul A., Long C.A., Miller L.H., Stowers A.W. (2002). In vitro studies with recombinant *Plasmodium falciparum* apical membrane antigen 1 (AMA1): Production and activity of an AMA1 vaccine and generation of a multiallelic response. Infect. Immunol..

[243] Mathews E.S., Jezewski A.J., Odom John A.R. (2021). Protein Prenylation and Hsp40 in Thermotolerance of *Plasmodium falciparum* Malaria Parasites. mBio.

[244] Josling G.A., Williamson K.C., Llinás M. (2018). Regulation of Sexual Commitment and Gametocytogenesis in Malaria Parasites. Annu. Rev. Microbiol..

[245] Tibúrcio M., Silvestrini F., Bertuccini L., Sander A.F., Turner L., Lavstsen T., Alano P. (2013). Early gametocytes of the malaria parasite *Plasmodium falciparum* specifically remodel the adhesive properties of infected erythrocyte surface. Cell. Microbiol..

[246] Shrestha S., Lucky A.B., Brashear A.M., Li X., Cui L., Miao J. (2022). Distinct Histone Post-translational Modifications during *Plasmodium falciparum* Gametocyte Development. J. Proteome Res..

[247] Jamjoom G.A. (2017). Evidence for a role of hemozoin in metabolism and gametocytogenesis. MalariaWorld J..

[248] Orjih A.U. (2012). Hemozoin accumulation in Garnham bodies of *Plasmodium falciparum* gametocytes. Parasitol. Res..

[249] Khan S.M., Franke-Fayard B., Mair G.R., Lasonder E., Janse C.J., Mann M., Waters A.P. (2005). Proteome analysis of separated male and female gametocytes reveals novel sex-specific Plasmodium biology. Cell.

[250] Lasonder E., Rijpma S.R., van Schaijk B.C., Hoeijmakers W.A., Kensche P.R., Gresnigt M.S., Italiaander A., Vos M.W., Woestenenk R., Bousema T. (2016). Integrated transcriptomic and proteomic analyses of *P. falciparum* gametocytes: Molecular insight into sex-specific processes and translational repression. Nucleic Acids Res..

[251] Santolamazza F., Avellino P., Siciliano G., Yao F.A., Lombardo F., Ouédraogo J.B., Modiano D., Alano P., Mangano V.D. (2017). Detection of *Plasmodium falciparum* male and female gametocytes and determination of parasite sex ratio in human endemic populations by novel, cheap and robust RTqPCR assays. Malar. J..

[252] Balestra A.C., Zeeshan M., Rea E., Pasquarello C., Brusini L., Mourier T., Subudhi A.K., Klages N., Arboit P., Pandey R. (2020). A divergent cyclin/cyclin-dependent kinase complex controls the atypical replication of a malaria parasite during gametogony and transmission. eLife.

[253] Camarda G., Bertuccini L., Singh S.K., Salzano A.M., Lanfrancotti A., Olivieri A., Scaloni A., Sharma A., Alano P. (2010). Regulated oligomerisation and molecular interactions of the early gametocyte protein Pfg27 in *Plasmodium falciparum* sexual differentiation. Int. J. Parasitol..

[254] Sannella A.R., Olivieri A., Bertuccini L., Ferrè F., Severini C., Pace T., Alano P. (2012). Specific tagging of the egress-related osmiophilic bodies in the gametocytes of *Plasmodium falciparum*. Malar. J..

[255] Suárez-Cortés P., Sharma V., Bertuccini L., Costa G., Bannerman N.L., Sannella A.R., Williamson K., Klemba M., Levashina E.A., Lasonder E. (2016). Comparative Proteomics and Functional Analysis Reveal a Role of *Plasmodium falciparum* Osmiophilic Bodies in Malaria Parasite Transmission. Mol. Cell. Proteom..

[256] Silvestrini F., Lasonder E., Olivieri A., Camarda G., van Schaijk B., Sanchez M., Younis Younis S., Sauerwein R., Alano P. (2010). Protein export marks the early phase of gametocytogenesis of the human malaria parasite *Plasmodium falciparum*. Mol. Cell. Proteom..

[257] Grasso F., Fratini F., Albanese T.G., Mochi S., Ciardo M., Pace T., Ponzi M., Pizzi E., Olivieri A. (2022). Identification and preliminary characterization of *Plasmodium falciparum* proteins secreted upon gamete formation. Sci. Rep..

[258] Jennison C., Lucantoni L., O’Neill M.T., McConville R., Erickson S.M., Cowman A.F., Sleebs B.E., Avery V.M., Boddey J.A. (2019). Inhibition of Plasmepsin V Activity Blocks *Plasmodium falciparum* Gametocytogenesis and Transmission to Mosquitoes. Cell Rep..

[259] Abugri J., Ayariga J., Sunwiale S.S., Wezena C.A., Gyamfi J.A., Adu-Frimpong M., Agongo G., Dongdem J.T., Abugri D., Dinko B. (2022). Targeting the *Plasmodium falciparum* proteome and organelles for potential antimalarial drug candidates. Heliyon.

[260] Ngwa C.J., Kiesow M.J., Orchard L.M., Farrukh A., Llinás M., Pradel G. (2019). The G9a Histone Methyltransferase Inhibitor BIX-01294 Modulates Gene Expression during *Plasmodium falciparum* Gametocyte Development and Transmission. Int. J. Mol. Sci..

[261] Josling G.A., Russell T.J., Venezia J., Orchard L., van Biljon R., Painter H.J., Llinás M. (2020). Dissecting the role of PfAP2-G in malaria gametocytogenesis. Nat. Commun..

[262] Tay C.L., Jones M.L., Hodson N., Theron M., Choudhary J.S., Rayner J.C. (2016). Study of *Plasmodium falciparum* DHHC palmitoyl transferases identifies a role for PfDHHC9 in gametocytogenesis. Cell. Microbiol..

[263] Johnson N., Philip N. (2021). Beyond phosphorylation: Putative roles of post-translational modifications in Plasmodium sexual stages. Mol. Biochem. Parasitol..

[264] Wang P.P., Jiang X., Zhu L., Zhou D., Hong M., He L., Chen L., Yao S., Zhao Y., Chen G. (2022). A G-Protein-Coupled Receptor Modulates Gametogenesis via PKG-Mediated Signaling Cascade in *Plasmodium berghei*. Microbiol. Spectr..

[265] Ruberto A.A., Bourke C., Vantaux A., Maher S.P., Jex A., Witkowski B., Snounou G., Mueller I. (2022). Single-cell RNA sequencing of *Plasmodium vivax* sporozoites reveals stage- and species-specific transcriptomic signatures. PLoS Negl. Trop. Dis..

[266] Ouologuem D.T., Dara A., Kone A., Ouattara A., Djimde A.A. (2023). *Plasmodium falciparum* Development from Gametocyte to Oocyst: Insight from Functional Studies. Microorganisms.

[267] Invergo B.M., Brochet M., Yu L., Choudhary J., Beltrao P., Billker O. (2017). Sub-minute Phosphoregulation of Cell Cycle Systems during Plasmodium Gamete Formation. Cell Rep..

[268] Garcia C.H.S., Depoix D., Queiroz R.M.L., Souza J.M.F., Fontes W., de Sousa M.V., Santos M.D.M., Carvalho P.C., Grellier P., Charneau S. (2018). Dynamic molecular events associated to *Plasmodium berghei* gametogenesis through proteomic approach. J. Proteom..

[269] Alonso-Morales A., González-López L., Cázares-Raga F.E., Cortés-Martínez L., Torres-Monzón J.A., Gallegos-Pérez J.L., Rodríguez M.H., James A.A., Hernández-Hernández Fde L. (2015). Protein phosphorylation during *Plasmodium berghei* gametogenesis. Exp. Parasitol..

[270] Hall N., Karras M., Raine J.D., Carlton J.M., Kooij T.W., Berriman M., Florens L., Janssen C.S., Pain A., Christophides G.K. (2005). A comprehensive survey of the Plasmodium life cycle by genomic, transcriptomic, and proteomic analyses. Science.

[271] Lasonder E., Janse C.J., van Gemert G.J., Mair G.R., Vermunt A.M., Douradinha B.G., van Noort V., Huynen M.A., Luty A.J., Kroeze H. (2008). Proteomic profiling of Plasmodium sporozoite maturation identifies new proteins essential for parasite development and infectivity. PLoS Pathog..

[272] Preira C.M.F., Pizzi E., Fratini F., Grasso F., Boccolini D., Mochi S., Favia G., Piselli E., Damiani C., Siden-Kiamos I. (2024). A time point proteomic analysis reveals protein dynamics of Plasmodium oocysts. Mol. Cell. Proteom..

[273] Armistead J.S., Jennison C., O’Neill M.T., Lopaticki S., Liehl P., Hanson K.K., Annoura T., Rajasekaran P., Erickson S.M., Tonkin C.J. (2018). *Plasmodium falciparum* subtilisin-like ookinete protein SOPT plays an important and conserved role during ookinete infection of the Anopheles stephensi midgut. Mol. Microbiol..

[274] Han Y.S., Thompson J., Kafatos F.C., Barillas-Mury C. (2000). Molecular interactions between Anopheles stephensi midgut cells and *Plasmodium berghei*: The time bomb theory of ookinete invasion of mosquitoes. EMBO J..

[275] Zeeshan M., Rea E., Abel S., Vukušić K., Markus R., Brady D., Eze A., Rashpa R., Balestra A.C., Bottrill A.R. (2023). Plasmodium ARK2 and EB1 drive unconventional spindle dynamics, during chromosome segregation in sexual transmission stages. Nat. Commun..

[276] Guttery D.S., Poulin B., Ferguson D.J., Szoor B., Wickstead B., Carroll P.L., Ramakrishnan C., Brady D., Patzewitz E.M., Straschil U. (2012). A unique protein phosphatase with kelch-like domains (PPKL) in Plasmodium modulates ookinete differentiation, motility and invasion. PLoS Pathog..

[277] Gupta A.P., Chin W.H., Zhu L., Mok S., Luah Y.H., Lim E.H., Bozdech Z. (2013). Dynamic epigenetic regulation of gene expression during the life cycle of malaria parasite *Plasmodium falciparum*. PLoS Pathog..

[278] von Gruning H., Coradin M., Mendoza M.R., Reader J., Sidoli S., Garcia B.A., Birkholtz L.M. (2022). A Dynamic and Combinatorial Histone Code Drives Malaria Parasite Asexual and Sexual Development. Mol. Cell. Proteom..

[279] Rashpa R., Klages N., Schvartz D., Pasquarello C., Brochet M. (2023). The Skp1-Cullin1-FBXO1 complex is a pleiotropic regulator required for the formation of gametes and motile forms in *Plasmodium berghei*. Nat. Commun..

[280] Wetzel J., Herrmann S., Swapna L.S., Prusty D., John Peter A.T., Kono M., Saini S., Nellimarla S., Wong T.W., Wilcke L. (2015). The role of palmitoylation for protein recruitment to the inner membrane complex of the malaria parasite. J. Biol. Chem..

[281] Santos J.M., Duarte N., Kehrer J., Ramesar J., Avramut M.C., Koster A.J., Dessens J.T., Frischknecht F., Chevalley-Maurel S., Janse C.J. (2016). Maternally supplied S-acyl-transferase is required for crystalloid organelle formation and transmission of the malaria parasite. Proc. Natl. Acad. Sci. USA.

[282] Wang X., Qian P., Cui H., Yao L., Yuan J. (2020). A protein palmitoylation cascade regulates microtubule cytoskeleton integrity in Plasmodium. EMBO J..

[283] Kolli S.K., Molina-Cruz A., Araki T., Geurten F.J.A., Ramesar J., Chevalley-Maurel S., Kroeze H.J., Bezemer S., de Korne C., Withers R. (2022). Malaria parasite evades mosquito immunity by glutaminyl cyclase-mediated posttranslational protein modification. Proc. Natl. Acad. Sci. USA.

[284] Pinheiro-Silva R., Borges L., Coelho L.P., Cabezas-Cruz A., Valdés J.J., do Rosário V., de la Fuente J., Domingos A. (2015). Gene expression changes in the salivary glands of Anopheles coluzzii elicited by *Plasmodium berghei* infection. Parasit. Vectors.

[285] Klaus S., Binder P., Kim J., Machado M., Funaya C., Schaaf V., Klaschka D., Kudulyte A., Cyrklaff M., Laketa V. (2022). Asynchronous nuclear cycles in multinucleated *Plasmodium falciparum* facilitate rapid proliferation. Sci. Adv..

[286] Kaushik M., Nehra A., Gill S.S., Gill R. (2020). Unraveling CAF-1 family in *Plasmodium falciparum*: Comparative genome-wide identification and phylogenetic analysis among eukaryotes, expression profiling and protein-protein interaction studies. 3 Biotech.

[287] Fang H., Klages N., Baechler B., Hillner E., Yu L., Pardo M., Choudhary J., Brochet M. (2017). Multiple short windows of calcium-dependent protein kinase 4 activity coordinate distinct cell cycle events during *Plasmodium* gametogenesis. eLife.

[288] Mair G.R., Lasonder E., Garver L.S., Franke-Fayard B.M., Carret C.K., Wiegant J.C., Dirks R.W., Dimopoulos G., Janse C.J., Waters A.P. (2010). Universal features of post-transcriptional gene regulation are critical for Plasmodium zygote development. PLoS Pathog..

[289] Singh M., Suryanshu, Kanika, Singh G., Dubey A., Chaitanya R.K. (2021). Plasmodium’s journey through the, A.nopheles mosquito: A comprehensive review. Biochimie.

[290] Klug D., Blandin S.A. (2023). Activation of complement-like antiparasitic responses in Anopheles mosquitoes. Curr. Opin. Microbiol..

[291] Bansal A., Molina-Cruz A., Brzostowski J., Mu J., Miller L.H. (2017). *Plasmodium falciparum* Calcium-Dependent Protein Kinase 2 Is Critical for Male Gametocyte Exflagellation but Not Essential for Asexual Proliferation. mBio.

[292] Lopaticki S., McConville R., John A., Geoghegan N., Mohamed S.D., Verzier L., Steel R.W.J., Evelyn C., O’Neill M.T., Soler N.M. (2022). Tryptophan C-mannosylation is critical for *Plasmodium falciparum* transmission. Nat. Commun..

[293] Yadav D.K., Kumar S., Teli M.K., Yadav R., Chaudhary S. (2019). Molecular Targets for Malarial Chemotherapy: A Review. Curr. Top. Med. Chem..

[294] Alves F.M., Bellei J.C.B., Barbosa C.S., Duarte C.L., Fonseca A.L.D., Pinto A.C.S., Raimundo F.O., Carpinter B.A., Lemos A.S.O., Coimbra E.S. (2022). Rational-Based Discovery of Novel β-Carboline Derivatives as Potential Antimalarials: From In Silico Identification of Novel Targets to Inhibition of Experimental Cerebral Malaria. Pathogens.

[295] Mogwera K.S.P., Chibale K., Arendse L.B. (2023). Developing kinase inhibitors for malaria: An opportunity or liability?. Trends. Parasitol..

[296] Adderley J., Doerig C. (2022). Comparative analysis of the kinomes of *Plasmodium falciparum*, *Plasmodium vivax* and their host Homo sapiens. BMC Genom..

[297] Munro B.A., McMorran B.J. (2022). Antimalarial Drug Strategies to Target Plasmodium Gametocytes. Parasitologia.

[298] Baker D.A., Matralis A.N., Osborne S.A., Large J.M., Penzo M. (2020). Targeting the Malaria Parasite cGMP-Dependent Protein Kinase to Develop New Drugs. Front. Microbiol..

[299] Vanaerschot M., Murithi J.M., Pasaje C.F.A., Ghidelli-Disse S., Dwomoh L., Bird M., Spottiswoode N., Mittal N., Arendse L.B., Owen E.S. (2020). Inhibition of Resistance-Refractory *P. falciparum* Kinase PKG Delivers Prophylactic, Blood Stage, and Transmission-Blocking Antiplasmodial Activity. Cell. Chem. Biol..

[300] Diaz C.A., Allocco J., Powles M.A., Yeung L., Donald R.G., Anderson J.W., Liberator P.A. (2006). Characterization of *Plasmodium falciparum* cGMP-dependent protein kinase (PfPKG): Antiparasitic activity of a PKG inhibitor. Mol. Biochem. Parasitol..

[301] Sahu N.K., Kohli D.V. (2012). Structural insight for imidazopyridazines as malarial kinase PfPK7 inhibitors using QSAR techniques. Med. Chem..

[302] Klein M., Dinér P., Dorin-Semblat D., Doerig C., Grøtli M. (2009). Synthesis of 3-(1,2,3-triazol-1-yl)- and 3-(1,2,3-triazol-4-yl)-substituted pyrazolo [3,4-d]pyrimidin-4-amines via click chemistry: Potential inhibitors of the *Plasmodium falciparum* PfPK7 protein kinase. Org. Biomol. Chem..

[303] Bouloc N., Large J.M., Smiljanic E., Whalley D., Ansell K.H., Edlin C.D., Bryans J.S. (2008). Synthesis and in vitro evaluation of imidazopyridazines as novel inhibitors of the malarial kinase PfPK7. Bioorg. Med. Chem. Lett..

[304] Sinha S., Batovska D.I., Medhi B., Radotra B.D., Bhalla A., Markova N., Sehgal R. (2019). In vitro anti-malarial efficacy of chalcones: Cytotoxicity profile, mechanism of action and their effect on erythrocytes. Malar. J..

[305] Ali A.H., Sudi S., Basir R., Embi N., Sidek H.M. (2017). The Antimalarial Effect of Curcumin Is Mediated by the Inhibition of Glycogen Synthase Kinase-3β. J. Med. Food.

[306] Arendse L.B., Wyllie S., Chibale K., Gilbert I.H. (2021). *Plasmodium* Kinases as Potential Drug Targets for Malaria: Challenges and Opportunities. ACS Infect. Dis..

[307] Mustière R., Vanelle P., Primas N. (2020). Plasmodial Kinase Inhibitors Targeting Malaria: Recent Developments. Molecules.

[308] Pantaleo A., Ferru E., Pau M.C., Khadjavi A., Mandili G., Mattè A., Spano A., De Franceschi L., Pippia P., Turrini F. (2016). Band 3 Erythrocyte Membrane Protein Acts as Redox Stress Sensor Leading to Its Phosphorylation by p (72) Syk. Oxid. Med. Cell. Longev..

[309] Pantaleo A., Kesely K.R., Pau M.C., Tsamesidis I., Schwarzer E., Skorokhod O.A., Chien H.D., Ponzi M., Bertuccini L., Low P.S. (2017). Syk inhibitors interfere with erythrocyte membrane modification during P falciparum growth and suppress parasite egress. Blood.

[310] Tsamesidis I., Reybier K., Marchetti G., Pau M.C., Virdis P., Fozza C., Nepveu F., Low P.S., Turrini F.M., Pantaleo A. (2020). Syk Kinase Inhibitors Synergize with Artemisinins by Enhancing Oxidative Stress in *Plasmodium falciparum*-Parasitized Erythrocytes. Antioxidants.

[311] Baragaña B., Hallyburton I., Lee M.C., Norcross N.R., Grimaldi R., Otto T.D., Proto W.R., Blagborough A.M., Meister S., Wirjanata G. (2015). A novel multiple-stage antimalarial agent that inhibits protein synthesis. Nature.

[312] Khandelwal A., Arez F., Alves P.M., Badolo L., Brito C., Fischli C., Fontinha D., Oeuvray C., Prudêncio M., Rottmann M. (2022). Translation of liver stage activity of M5717, a Plasmodium elongation factor 2 inhibitor: From bench to bedside. Malar. J..

[313] McCarthy J.S., Yalkinoglu Ö., Odedra A., Webster R., Oeuvray C., Tappert A., Bezuidenhout D., Giddins M.J., Dhingra S.K., Fidock D.A. (2021). Safety, pharmacokinetics, and antimalarial activity of the novel plasmodium eukaryotic translation elongation factor 2 inhibitor M5717: A first-in-human, randomised, placebo-controlled, double-blind, single ascending dose study and volunteer infection study. Lancet Infect. Dis..

[314] Deu E. (2017). Proteases as antimalarial targets: Strategies for genetic, chemical, and therapeutic validation. FEBS J..

[315] Ng C.L., Fidock D.A., Bogyo M. (2017). Protein Degradation Systems as Antimalarial Therapeutic Targets. Trends. Parasitol..

[316] Madhav H., Patel T.S., Rizvi Z., Reddy G.S., Rahman A., Rahman M.A., Ahmedi S., Fatima S., Saxena K., Manzoor N. (2023). Development of diphenylmethylpiperazine hybrids of chloroquinoline and triazolopyrimidine using Petasis reaction as new cysteine proteases inhibitors for malaria therapeutics. Eur. J. Med. Chem..

[317] Skorokhod O., Valente E., Mandili G., Ulliers D., Schwarzer E. (2023). Micromolar Dihydroartemisinin Concentrations Elicit Lipoperoxidation in *Plasmodium falciparum*-Infected Erythrocytes. Antioxidants.

[318] Li X., Chen H., Bahamontes-Rosa N., Kun J.F., Traore B., Crompton P.D., Chishti A.H. (2009). *Plasmodium falciparum* signal peptide peptidase is a promising drug target against blood stage malaria. Biochem. Biophys. Res. Commun..

[319] Parvanova I., Epiphanio S., Fauq A., Golde T.E., Prudêncio M., Mota M.M. (2009). A Small Molecule Inhibitor of Signal Peptide Peptidase Inhibits Plasmodium Development in the Liver and Decreases Malaria Severity. PLoS ONE.

[320] Harbut M.B., Patel B.A., Yeung B.K., McNamara C.W., Bright A.T., Ballard J., Supek F., Golde T.E., Winzeler E.A., Diagana T.T. (2012). Targeting the ERAD pathway via inhibition of signal peptide peptidase for antiparasitic therapeutic design. Proc. Natl. Acad. Sci. USA.

[321] Pino P., Caldelari R., Mukherjee B., Vahokoski J., Klages N., Maco B., Collins C.R., Blackman M.J., Kursula I., Heussler V. (2017). A multistage antimalarial targets the plasmepsins IX and X essential for invasion and egress. Science.

[322] Lisauskaitė M., Nixon G.L., Woodley C.M., Berry N.G., Coninckx A., Qie L.C., Leung S.C., Taramelli D., Basilico N., Parapini S. (2023). Design, synthesis and modelling of photoreactive chemical probes for investigating target engagement of plasmepsin IX and X in *Plasmodium falciparum*. RSC Chem. Biol..

[323] McConville M., Fernández J., Angulo-Barturen Í., Bahamontes-Rosa N., Ballell-Pages L., Castañeda P., de Cózar C., Crespo B., Guijarro L., Jiménez-Díaz M.B. (2015). Carbamoyl Triazoles, Known Serine Protease Inhibitors, Are a Potent New Class of Antimalarials. J. Med. Chem..

[324] Nizi E., Sferrazza A., Fabbrini D., Nardi V., Andreini M., Graziani R., Gennari N., Bresciani A., Paonessa G., Harper S. (2018). Peptidomimetic nitrile inhibitors of malarial protease falcipain-2 with high selectivity against human cathepsins. Bioorg. Med. Chem. Lett..

[325] Sharma P., Wollenberg K., Sellers M., Zainabadi K., Galinsky K., Moss E., Nguitragool W., Neafsey D., Desai S.A. (2013). An epigenetic antimalarial resistance mechanism involving parasite genes linked to nutrient uptake. J. Biol. Chem..

[326] Mira-Martínez S., Rovira-Graells N., Crowley V.M., Altenhofen L.M., Llinás M., Cortés A. (2013). Epigenetic switches in clag3 genes mediate blasticidin S resistance in malaria parasites. Cell. Microbiol..

[327] Rawat M., Kanyal A., Sahasrabudhe A., Vembar S.S., Lopez-Rubio J.J., Karmodiya K. (2021). Histone acetyltransferase PfGCN5 regulates stress responsive and artemisinin resistance related genes in *Plasmodium falciparum*. Sci. Rep..

[328] Lucky A.B., Wang C., Shakri A.R., Kalamuddin M., Chim-Ong A., Li X., Miao J. (2023). *Plasmodium falciparum* GCN5 plays a key role in regulating artemisinin resistance-related stress responses. Antimicrob. Agents Chemother..

[329] Kumar A., Bhowmick K., Vikramdeo K.S., Mondal N., Subbarao N., Dhar S.K. (2017). Designing novel inhibitors against histone acetyltransferase (HAT: GCN5) of *Plasmodium falciparum*. Eur. J. Med. Chem..

[330] Sen U., Nayak A., Khurana J., Sharma D., Gupta A. (2020). Inhibition of PfMYST Histone Acetyltransferase Activity Blocks *Plasmodium falciparum* Growth and Survival. Antimicrob. Agents Chemother..

[331] Sabnis R.W. (2021). Novel Histone Acetyltransferase (HAT) Inhibitors for Treating Diseases. ACS Med. Chem. Lett..

[332] Andrews K.T., Tran T.N., Lucke A.J., Kahnberg P., Le G.T., Boyle G.M., Gardiner D.L., Skinner-Adams T.S., Fairlie D.P. (2008). Potent antimalarial activity of histone deacetylase inhibitor analogues. Antimicrob. Agents Chemother..

[333] Andrews K.T., Gupta A.P., Tran T.N., Fairlie D.P., Gobert G.N., Bozdech Z. (2012). Comparative gene expression profiling of *P. falciparum* malaria parasites exposed to three different histone deacetylase inhibitors. PLoS ONE.

[334] Jublot D., Cavaillès P., Kamche S., Francisco D., Fontinha D., Prudêncio M., Guichou J.-F., Labesse G., Sereno D., Loeuillet C. (2022). A Histone Deacetylase (HDAC) Inhibitor with Pleiotropic In Vitro Anti-Toxoplasma and Anti-Plasmodium Activities Controls Acute and Chronic Toxoplasma Infection in Mice. Int. J. Mol. Sci..

[335] Mohapatra T.K., Nayak R.R., Ganeshpurkar A., Tiwari P., Kumar D. (2024). Opportunities and Difficulties in the Repurposing of HDAC Inhibitors as Antiparasitic Agents. Drugs Drug Candidates.

[336] von Bredow L., Schäfer T.M., Hogenkamp J., Tretbar M., Stopper D., Kraft F.B., Schliehe-Diecks J., Schöler A., Borkhardt A., Bhatia S. (2022). Synthesis, Antiplasmodial, and Antileukemia Activity of Dihydroartemisinin–HDAC Inhibitor Hybrids as Multitarget Drugs. Pharmaceuticals.

[337] Malmquist N.A., Sundriyal S., Caron J., Chen P., Witkowski B., Menard D., Suwanarusk R., Renia L., Nosten F., Jiménez-Díaz M.B. (2015). Histone methyltransferase inhibitors are orally bioavailable, fast-acting molecules with activity against different species causing malaria in humans. Antimicrob. Agents Chemother..

[338] Gomes A.R.Q., Cunha N., Varela E.L.P., Brígido H.P.C., Vale V.V., Dolabela M.F., De Carvalho E.P., Percário S. (2022). Oxidative Stress in Malaria: Potential Benefits of Antioxidant Therapy. Int. J. Mol. Sci..

[339] Zheng Z., Liu H., Wang X., Zhang Y., Qu S., Yang Y., Deng S., Chen L., Zhu X., Li Y. (2021). Artesunate and Tetramethylpyrazine Exert Effects on Experimental Cerebral Malaria in a Mechanism of Protein S-Nitrosylation. ACS Infect. Dis..

[340] Ben Mamoun C., Prigge S.T., Vial H. (2010). Targeting the Lipid Metabolic Pathways for the Treatment of Malaria. Drug Dev. Res..

[341] Schlott A.C., Holder A.A., Tate E.W. (2018). N-Myristoylation as a Drug Target in Malaria: Exploring the Role of N-Myristoyltransferase Substrates in the Inhibitor Mode of Action. ACS Infect. Dis..

[342] Schlott A.C., Mayclin S., Reers A.R., Coburn-Flynn O., Bell A.S., Green J., Knuepfer E., Charter D., Bonnert R., Campo B. (2019). Structure-Guided Identification of Resistance Breaking Antimalarial N-Myristoyltransferase Inhibitors. Cell. Chem. Biol..

[343] Kim J., Tan Y.Z., Wicht K.J., Erramilli S.K., Dhingra S.K., Okombo J., Vendome J., Hagenah L.M., Giacometti S.I., Warren A.L. (2019). Structure and drug resistance of the *Plasmodium falciparum* transporter PfCRT. Nature.

[344] Wurtz N., Fall B., Pascual A., Fall M., Baret E., Camara C., Nakoulima A., Diatta B., Fall K.B., Mbaye P.S. (2014). Role of Pfmdr1 in in vitro *Plasmodium falciparum* susceptibility to chloroquine, quinine, monodesethylamodiaquine, mefloquine, lumefantrine, and dihydroartemisinin. Antimicrob. Agents Chemother..

[345] Knak T., Abdullaziz M.A., Höfmann S., Alves Avelar L.A., Klein S., Martin M., Fischer M., Tanaka N., Kurz T. (2022). Over 40 Years of Fosmidomycin Drug Research: A Comprehensive Review and Future Opportunities. Pharmaceuticals.

[346] Bofill Verdaguer I., Sussmann R.A.C., Santiago V.F., Palmisano G., Moura G.C., Mesquita J.T., Yamaguchi L.F., Kato M.J., Katzin A.M., Crispim M. (2022). Isoprenoid alcohols utilization by malaria parasites. Front. Chem..

[347] Meshnick S.R. (1998). Artemisinin antimalarials: Mechanisms of action and resistance. Med. Trop. (Mars).

[348] Tilley L., Straimer J., Gnädig N.F., Ralph S.A., Fidock D.A. (2016). Artemisinin Action and Resistance in *Plasmodium falciparum*. Trends. Parasitol..

[349] Jourdan J., Walz A., Matile H., Schmidt A., Wu J., Wang X., Dong Y., Vennerstrom J.L., Schmidt R.S., Wittlin S. (2019). Stochastic Protein Alkylation by Antimalarial Peroxides. ACS Infect. Dis..

[350] Embo-Ibouanga A.W., Nguyen M., Paloque L., Coustets M., Joly J.P., Augereau J.M., Vanthuyne N., Bikanga R., Coquin N., Robert A. (2024). Hybrid Peptide-Alkoxyamine Drugs: A Strategy for the Development of a New Family of Antiplasmodial Drugs. Molecules.

[351] Keita A., Franetich J.F., Carraz M., Valentin L., Bordessoules M., Baron L., Bigeard P., Dupuy F., Geay V., Tefit M. (2022). Potent Antiplasmodial Derivatives of Dextromethorphan Reveal the Ent-Morphinan Pharmacophore of Tazopsine-Type Alkaloids. Pharmaceutics.

[352] Skorokhod O.A., Davalos-Schafler D., Gallo V., Valente E., Ulliers D., Notarpietro A., Mandili G., Novelli F., Persico M., Taglialatela-Scafati O. (2015). Oxidative stress-mediated antimalarial activity of plakortin, a natural endoperoxide from the tropical sponge Plakortis simplex. Free Radic. Biol. Med..

[353] Ismail H.M., Barton V., Phanchana M., Charoensutthivarakul S., Wong M.H., Hemingway J., Biagini G.A., O’Neill P.M., Ward S.A. (2016). Artemisinin activity-based probes identify multiple molecular targets within the asexual stage of the malaria parasites *Plasmodium falciparum* 3D7. Proc. Natl. Acad. Sci. USA.

[354] Talman A.M., Clain J., Duval R., Ménard R., Ariey F. (2019). Artemisinin Bioactivity and Resistance in Malaria Parasites. Trends. Parasitol..

[355] Eckstein-Ludwig U., Webb R.J., Van Goethem I.D., East J.M., Lee A.G., Kimura M., O’Neill P.M., Bray P.G., Ward S.A., Krishna S. (2003). Artemisinins target the SERCA of *Plasmodium falciparum*. Nature.

[356] Woodley C.M., Amado P.S.M., Cristiano M.L.S., O’Neill P.M. (2021). Artemisinin inspired synthetic endoperoxide drug candidates: Design, synthesis, and mechanism of action studies. Med. Res. Rev..

[357] Noel S., Sharma S., Shankar R., Rath S.K. (2008). Identification of differentially expressed genes after acute exposure to bulaquine (CDRI 80/53) in mice liver. Basic Clin. Pharmacol. Toxicol..

[358] Siciliano G., Di Paolo V., Rotili D., Migale R., Pedini F., Casella M., Camerini S., Dalzoppo D., Henderson R., Huijs T. (2022). The Nitrobenzoxadiazole Derivative NBDHEX Behaves as *Plasmodium falciparum* Gametocyte Selective Inhibitor with Malaria Parasite Transmission Blocking Activity. Pharmaceuticals.

[359] Florens L., Washburn M.P., Raine J.D., Anthony R.M., Grainger M., Haynes J.D., Moch J.K., Muster N., Sacci J.B., Tabb D.L. (2002). A proteomic view of the *Plasmodium falciparum* life cycle. Nature.

[360] Johnson J.R., Florens L., Carucci D.J., Yates J.R. (2004). Proteomics in malaria. J. Proteome Res..

[361] Gupta P., Venkadesan S., Mohanty D. (2022). Pf-Phospho: A machine learning-based phosphorylation sites prediction tool for Plasmodium proteins. Brief. Bioinform..

[362] Gunalan K., Rowley E.H., Miller L.H. (2020). A Way Forward for Culturing *Plasmodium vivax*. Trends. Parasitol..

[363] Menkin-Smith L., Winders W.T. (2024). Plasmodium vivax Malaria. StatPearls [Internet].

[364] Molina-Franky J., Reyes C., Picón Jaimes Y.A., Kalkum M., Patarroyo M.A. (2022). The Black Box of Cellular and Molecular Events of *Plasmodium vivax* Merozoite Invasion into Reticulocytes. Int. J. Mol. Sci..

[365] Divya M., Prabhu S.R., Satyamoorthy K., Saadi A.V. (2023). Therapeutics through glycobiology: An approach for targeted elimination of malaria. Biologia.

[366] Tran P.N., Brown S.H., Rug M., Ridgway M.C., Mitchell T.W., Maier A.G. (2016). Changes in lipid composition during sexual development of the malaria parasite *Plasmodium falciparum*. Malar. J..

[367] Tokumasu F., Hayakawa E.H., Fukumoto J., Tokuoka S.M., Miyazaki S. (2021). Creative interior design by *Plasmodium falciparum*: Lipid metabolism and the parasite’s secret chamber. Parasitol. Int..

[368] Spickett C.M. (2020). Formation of Oxidatively Modified Lipids as the Basis for a Cellular Epilipidome. Front. Endocrinol..

[369] Adigun R.A., Malan F.P., Balogun M.O., October N. (2022). Rational Optimization of Dihydropyrimidinone-Quinoline Hybrids as *Plasmodium falciparum* Glutathione Reductase Inhibitors. ChemMedChem.

[370] Shafi S., Gupta S., Jain R., Shoaib R., Munjal A., Maurya P., Kumar P., Kalam Najmi A., Singh S. (2023). Tackling the emerging Artemisinin-resistant malaria parasite by modulation of defensive oxido-reductive mechanism via nitrofurantoin repurposing. Biochem. Pharmacol..

[371] Berneburg I., Peddibhotla S., Heimsch K.C., Haeussler K., Maloney P., Gosalia P., Preuss J., Rahbari M., Skorokhod O., Valente E. (2022). An optimized dihydrodibenzothiazepine lead compound (SBI-0797750) as a potent and selective inhibitor of *Plasmodium falciparum* and *P. vivax* glucose 6-phosphate dehydrogenase 6-phosphogluconolactonase. Antimicrob. Agents Chemother..

[372] Skorokhod O., Vostokova E., Gilardi G. (2024). The role of P450 enzymes in malaria and other vector-borne infectious diseases. Biofactors.

[373] Phillips M.A., Rathod P.K. (2010). Plasmodium dihydroorotate dehydrogenase: A promising target for novel anti-malarial chemotherapy. Infect. Disord. Drug Targets.

[374] Gehlot P., Vyas V.K. (2023). Recent advances on patents of *Plasmodium falciparum* dihydroorotate dehydrogenase (*Pf*DHODH) inhibitors as antimalarial agents. Expert Opin. Ther. Pat..

[375] Lehane A.M., Ridgway M.C., Baker E., Kirk K. (2014). Diverse chemotypes disrupt ion homeostasis in the Malaria parasite. Mol. Microbiol..

[376] Santos B.M.D., Przyborski J.M., Garcia C.R.S. (2023). Changes in K^+^ Concentration as a Signaling Mechanism in the Apicomplexa Parasites Plasmodium and Toxoplasma. Int. J. Mol. Sci..

[377] Scarpelli P.H., Pecenin M.F., Garcia C.R.S. (2021). Intracellular Ca^2+^ Signaling in Protozoan Parasites: An Overview with a Focus on Mitochondria. Int. J. Mol. Sci..

[378] Kayamba F., Faya M., Pooe O.J., Kushwaha B., Kushwaha N.D., Obakachi V.A., Nyamori V.O., Karpoormath R. (2021). Lactate dehydrogenase and malate dehydrogenase: Potential antiparasitic targets for drug development studies. Bioorg. Med. Chem..

[379] Zhong W., Li K., Cai Q., Guo J., Yuan M., Wong Y.H., Walkinshaw M.D., Fothergill-Gilmore L.A., Michels P.A.M., Dedon P.C. (2020). Pyruvate kinase from *Plasmodium falciparum*: Structural and kinetic insights into the allosteric mechanism. Biochem. Biophys. Res. Commun..

[380] Dillenberger M., Werner A.-D., Velten A.-S., Rahlfs S., Becker K., Fritz-Wolf K. (2023). Structural Analysis of *Plasmodium falciparum* Hexokinase Provides Novel Information about Catalysis Due to a Plasmodium-Specific Insertion. Int. J. Mol. Sci..

[381] Veiga M.I., Peng W.K. (2020). Rapid phenotyping towards personalized malaria medicine. Malar. J..

[382] Williams D., Le Roch K.G., Ginsburg G.S., Willard H.F., Tsalik E.L., Woods C.W. (2019). Chapter 14-Genomics and precision medicine for malaria: A dream come true?. Genomic and Precision Medicine.

[383] Meng F., Liang Z., Zhao K., Luo C. (2021). Drug design targeting active posttranslational modification protein isoforms. Med. Res. Rev..

[384] Zhai L.H., Chen K.F., Hao B.B., Tan M.J. (2022). Proteomic characterization of post-translational modifications in drug discovery. Acta Pharmacol. Sin..

[385] Bah A., Forman-Kay J.D. (2016). Modulation of Intrinsically Disordered Protein Function by Post-translational Modifications. J. Biol. Chem..

[386] Chen L., Kashina A. (2021). Post-translational Modifications of the Protein Termini. Front. Cell Dev. Biol..

[387] Zhang W., Zhang J., MacRaild C.A., Norton R.S., Anders R.F., Zhang X. (2019). Modulation of the aggregation of an amyloidogenic sequence by flanking-disordered region in the intrinsically disordered antigen merozoite surface protein 2. Eur. Biophys. J..

[388] Ameri M., Nezafat N., Eskandari S. (2022). The potential of intrinsically disordered regions in vaccine development. Expert Rev. Vaccines.

[389] Niang E.H.A., Bassene H., Fenollar F., Mediannikov O. (2018). Biological Control of Mosquito-Borne Diseases: The Potential of Wolbachia-Based Interventions in an IVM Framework. J. Trop. Med..

[390] Mushtaq I., Sarwar M.S., Chaudhry A., Shah S.A.H., Ahmad M.M. (2024). Updates on traditional methods for combating malaria and emerging Wolbachia-based interventions. Front. Cell. Infect. Microbiol..

[391] Yu S., Wang J., Luo X., Zheng H., Wang L., Yang X., Wang Y. (2022). Transmission-Blocking Strategies Against Malaria Parasites During Their Mosquito Stages. Front. Cell. Infect. Microbiol..

[392] Vandana V., Dong S., Sheth T., Sun Q., Wen H., Maldonado A., Xi Z., Dimopoulos G. (2024). Wolbachia infection-responsive immune genes suppress *Plasmodium falciparum* infection in Anopheles stephensi. PLoS Pathog..

